# Controlled mutation in the replication of synthetic oligomers†

**DOI:** 10.1039/d0sc06770a

**Published:** 2021-02-05

**Authors:** Diego Núñez-Villanueva, Christopher A. Hunter

**Affiliations:** Yusuf Hamied Department of Chemistry, University of Cambridge Lensfield Road Cambridge CB2 1EW UK herchelsmith.orgchem@ch.cam.ac.uk

## Abstract

Replication of sequence information with mutation is the molecular basis for the evolution of functional biopolymers. Covalent template-directed synthesis has been used to replicate sequence information in synthetic oligomers, and the covalent base-pairs used in these systems provide an opportunity to manipulate the outcome of the information transfer process through the use of traceless linkers. Two new types of covalent base-pair have been used to introduce mutation in the replication of an oligotriazole, where information is encoded as the sequence of benzoic acid and phenol monomer units. When a benzoic acid–benzoic acid base-pairing system was used, a direct copy of a benzoic acid homo-oligomer template was obtained. When a phenol–benzoic acid base-pairing system was used, a reciprocal copy, the phenol homo-oligomer, was obtained. The two base-pairing systems are isosteric, so they can be used interchangeably, allowing direct and reciprocal copying to take place simultaneously on the same template strand. As a result, it was possible to introduce mutations in the replication process by spiking the monomer used for direct copying with the monomer used for reciprocal copying. The mutation rate is determined precisely by the relative proportions of the two monomers. The ability to introduce mutation at a controlled rate is a key step in the development of synthetic systems capable of evolution, which requires replication with variation.

## Abstract 2

The use of two different covalent base-pairs introduces sequence mutations at a controlled rate in the covalent template-directed synthesis of oligotriazoles, a step towards evolvable synthetic polymers.

## Graphical abstract

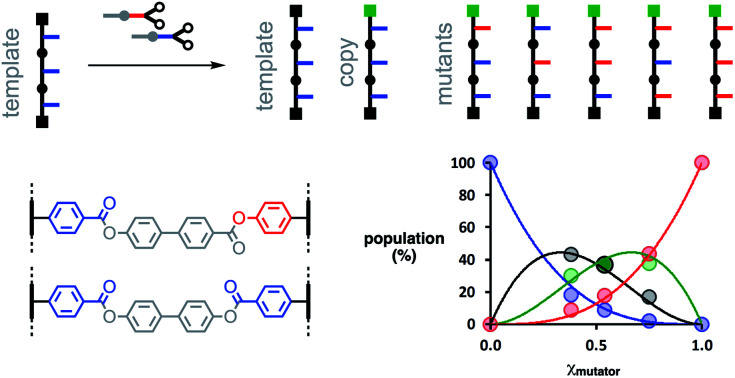

## Introduction

Sequence information transfer is the basis for the evolution of living systems.^1^ At the molecular level, information is transmitted from a parent to a daughter nucleic acid oligomer through a replication step, which is mediated by polymerase enzymes. A certain degree of mutation is required in this replication process for evolution to take place. Thus, the new oligomer sequences differ somewhat from the parent templates ensuring sequence variation and allowing evolutionary selection. Success in evolution requires a precise control of the mutation rate, which must be high enough to create a subtle variation in the sequences of the daughter strand population, but small enough to avoid the loss of the information encoded in the parent template.^2^ The principles governing molecular evolution, *i.e.* replication and mutation of sequence information, have been harnessed for the development of evolutionary processes to search chemical space.^3–6^ Directed evolution methods represent a powerful strategy for tailoring the function of biomolecules for therapeutic or manufacturing applications.^7–10^ However, current methods rely on nucleic acid replication, so the accessible chemical space is therefore limited to nucleic acids or proteins.^11–13^

**Fig. 1:**
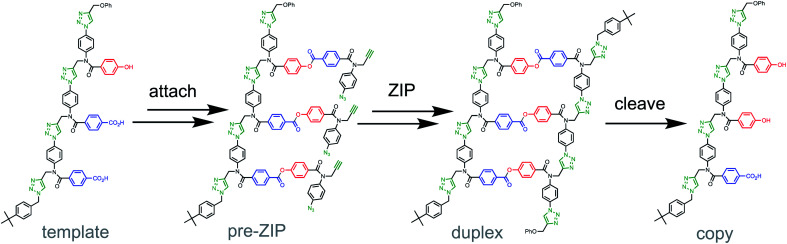
Sequence information transfer using covalent template-directed synthesis. In the attach step, complementary monomers are loaded onto the template using phenol–benzoic acid ester base-pairing chemistry. In the ZIP step, intramolecular CuAAC reactions lead to oligomerization of monomers on the template followed by capping of the chain ends. In the cleave step, hydrolysis of the ester bonds connecting the new oligomer to the template regenerates the template and releases the complementary copy strand.

Our aim is to extend molecular evolution to fully synthetic systems in order to access new non-natural oligomers with tailored properties defined by the sequence of the monomer building blocks. For that purpose, it is crucial to develop template-directed synthetic methods for the replication of information encoded in synthetic oligomers.^14–21^ We have recently demonstrated how covalent template-directed synthesis can be used to transfer of sequence information from a parent template to a sequence-complementary copy (Fig. 1).^22,23^

The first step in Fig. 1 is attachment of the monomer building blocks to the template using covalent base-pairing based on the formation of an ester between a phenol (P) and a benzoic acid (A).^22^ Copper catalyzed azide alkyne cycloaddition (CuAAC) is used in the ZIP step to give the covalent duplex,^24,25^ and cleavage of the ester base-pairs regenerates the template along with the complementary copy. If such synthetic replication systems are eventually to be implemented in evolution experiments, the other key requirement is a method for introduction of variation through mutation. Here we describe a strategy for controlled mutation of the sequence information contained in a population of copy strands in covalent template-directed replication.

## Approach

**Fig. 2:**
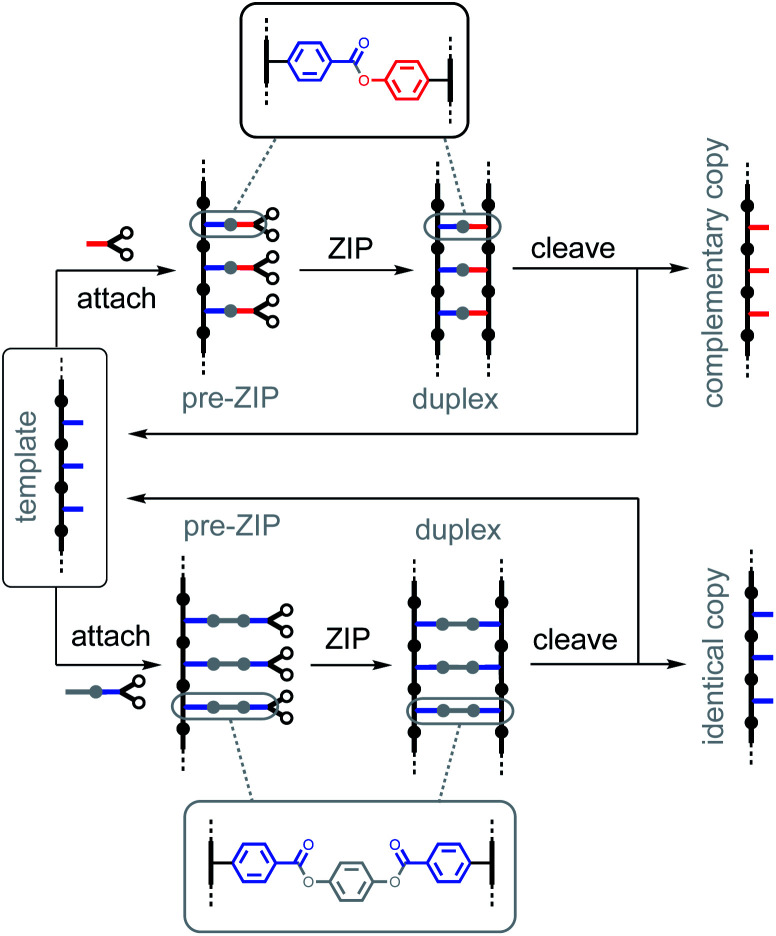
The information transferred from the template to the daughter strand can be programmed by using different covalent base-pairing systems. Top channel: base pairs formed by the direct attachment of phenol and benzoic acid give rise to a copy strand which is complementary to the template. Bottom channel: base-pairs formed by connecting two benzoic acids with a linker give rise to an identical copy of the template strand.

An interesting feature of covalent base-pairing is that the information that is transferred in the replication process can be adjusted by changing the chemical structure of the base-pair. For example, Fig. 2 shows how a traceless linker was used to make an identical copy rather than a complementary copy of the sequence information in a template strand.^23^ Direct coupling between phenol and benzoic acid units leads to reciprocal replication of chemical information, analogous to nucleic acid replication (Fig. 2, top channel).^22^ However, if a linker is used to connect two benzoic acid units in a symmetric base-pair, then the replication process yields an identical copy of the template strand (Fig. 2, bottom channel).^23^ Here we apply this traceless linker concept to develop a new set of covalent base-pairs, which can be used to introduce mutations into a covalent template-directed replication process and to tune the mutation rate in a predictable manner.

**Fig. 3:**
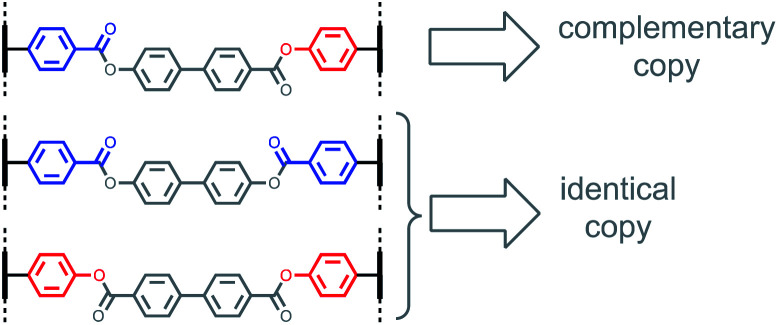
Isosteric base-pairs can be used interchangeably to control how sequence information is transferred in covalent template-directed replication of phenol–benzoic acid oligomers. Unsymmetric base-pairs gives rise to reciprocal replication (sequence-complementary copies), and symmetric base-pairs give rise to direct replication (identical copies).

Fig. 3 shows base-pairing systems that could be used for formation of either identical or complementary sequence copies of a template strand. There is one key difference from the base-pairing systems shown in Fig. 2, which is that the three base-pairs in Fig. 3 are all isosteric. These three base-pairs can therefore be accommodated interchangeably within the same product duplex and used simultaneously in the same replication process. By attaching monomer units to a template using mixtures of symmetric and unsymmetric base-pairs, it should be possible to obtain a mixture of reciprocal and direct copying of sequence information in the replication process, thereby introducing mutation.

**Fig. 4:**
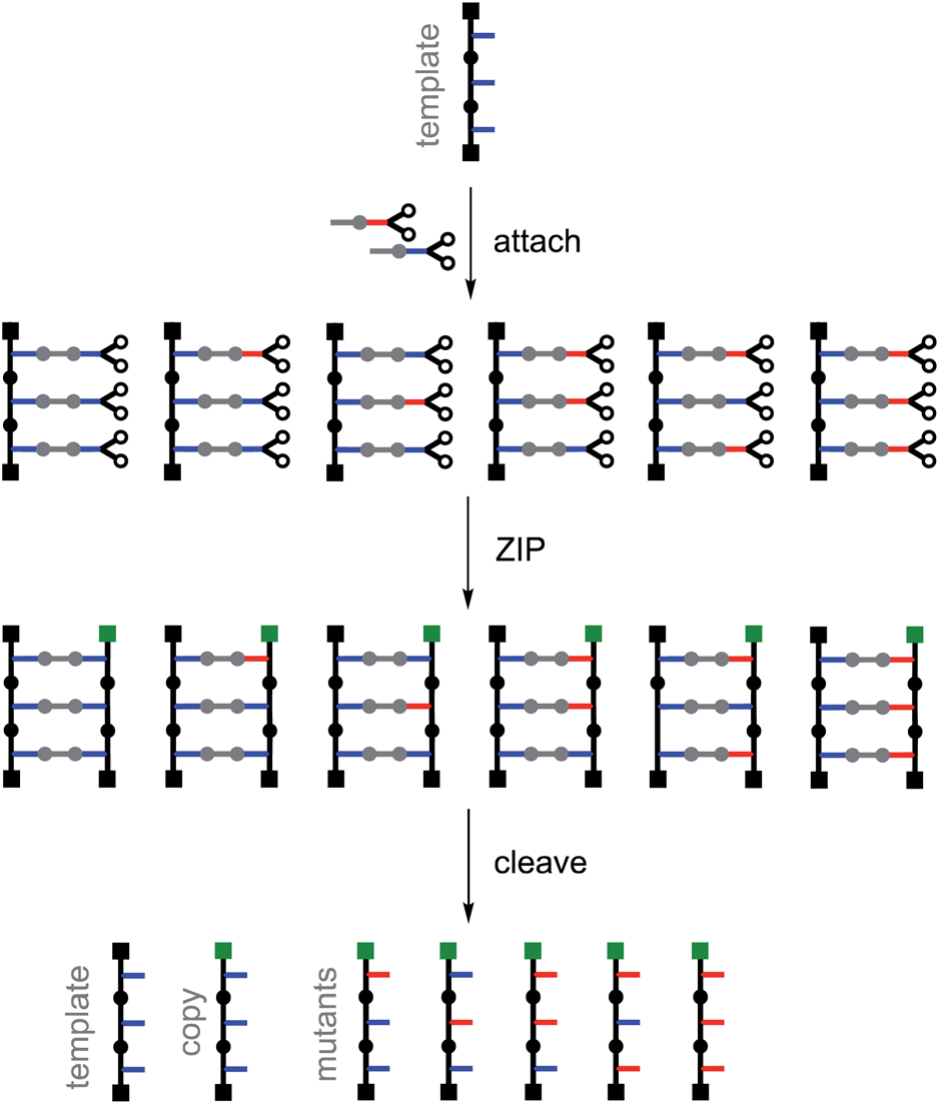
Replication with mutation. A mixture of two different monomers is used in the attach step to generate a mixture of symmetric and unsymmetric base-pairs. After the ZIP and cleave steps, the template is regenerated along with a direct copy and all possible mutant sequences. The products of the replication process can be distinguished from the template by different end capping groups (green) used in the ZIP step.

Fig. 4 illustrates the principle for replication of a homo-oligomer. If a mixture of two different monomers is used in the attach step, all possible stochastic combinations of the symmetric and the unsymmetric base-pairs will be obtained in the pre-ZIP intermediate. After the ZIP and cleave steps, the template is regenerated along with an identical copy and all possible mutant sequences. The mutation rate can be controlled simply by changing the relative amounts of the two monomers used in the attach step. The experiments described below demonstrate realization of the error-prone replication process illustrated in Fig. 4 by using the isosteric base-pairing system shown in Fig. 3.

## Results and discussion

**Scheme 1:**
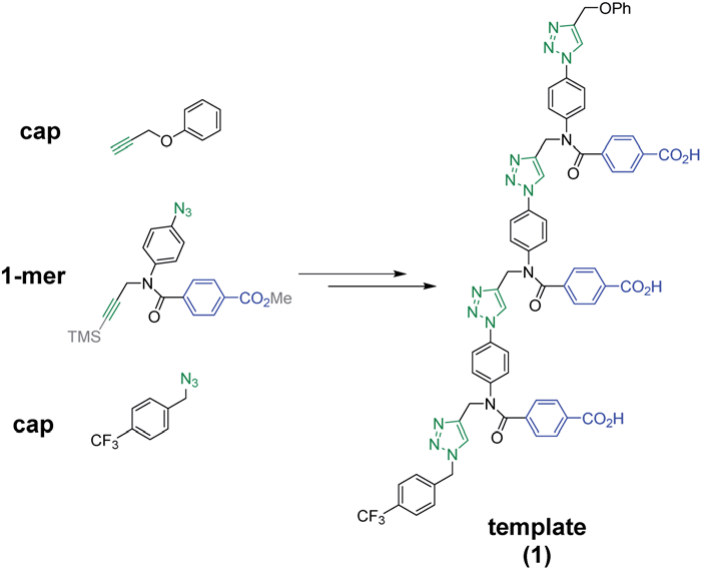
Structure of template **1**.

Scheme 1 shows the structure of the homosequence template (**1**) used in this study. The 3-mer template was obtained as described previously from the corresponding TMS-protected 1-mer by CuAAC capping with phenyl propargyl ether, followed by sequential deprotection and CuAAC coupling with more monomer. The terminal alkyne was then capped with 4-trifluoromethylbenzyl azide, and the methyl esters were hydrolysed to reveal the carboxylic acids required for base-pair formation.^23^

**Scheme 2:**
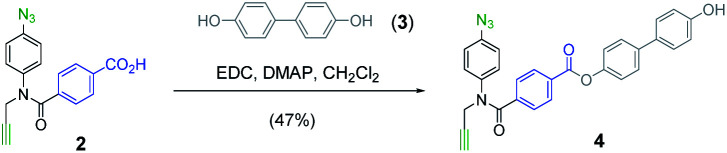
Synthesis of **4**.

**Scheme 3:**
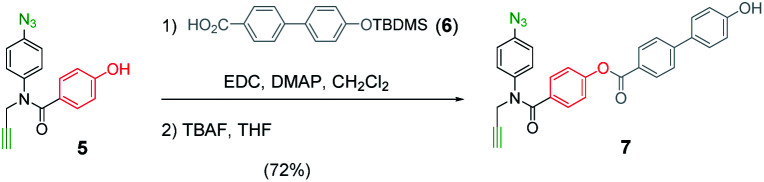
Synthesis of **7**.

The mutation protocol shown in Fig. 4 requires two different monomers to be attached simultaneously to the template. Scheme 2 shows the synthesis of monomer **4** bearing a 4,4′-biphenol linker, which encodes for the symmetric benzoic acid–benzoic acid base-pair. Ester coupling of benzoic acid **2** with excess 4,4′-biphenol yielded **4** in moderate yield.^22^Scheme 3 shows the synthesis of monomer **7** bearing a 4-4′-hydroxy-4-biphenylcarboxylic acid linker, which encodes for the unsymmetric phenol–benzoic acid base-pair. Ester coupling of phenol **5** with silyl-protected 4-4′-hydroxy-4-biphenylcarboxylic acid (**6**) followed by TBAF-mediated removal of the phenol protecting group afforded **7** in good yield.^22^

**Fig. 5:**
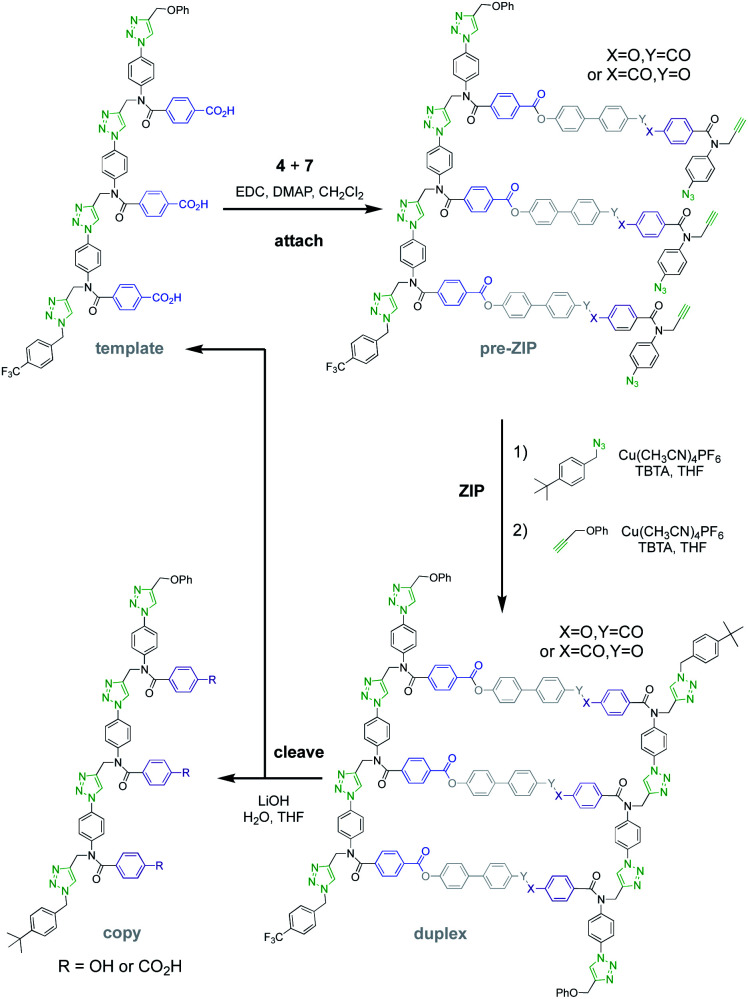
Covalent template-directed replication and mutation of the chemical information encoded in template **1**. Ester base-pair chemistry was used to attach a mixture of monomers **4** and **7** to the template. The resulting pre-ZIP intermediates were subjected to intramolecular CuAAC reaction in the presence of 4-*t*-butylbenzyl azide to block the alkyne chain end. The azide chain end was capped with phenyl propargyl ether to give the corresponding duplexes (only the antiparallel arrangement of the backbones is shown, but parallel is also possible). Hydrolysis of the ester base-pairs in the cleave step regenerated the template together with the templated products (**8–15**). The copied products are distinguished from the original template by the terminal *t*-butyl group.

Fig. 5 shows the reaction steps carried out to mutate the information encoded in template **1***via* covalent template-directed replication. The first step is attachment of a mixture of monomers **4** and **7** to the template *via* ester bond formation to obtain the corresponding mixture of pre-ZIP intermediates. We carried out five independent experiments with different ratios of monomer **4**, which would result in a direct copy of the template sequence, and monomer **7**, which leads to mutation. ^1^H NMR analysis of the mixture of pre-ZIP intermediates indicated that the reactivity of the two monomers used in the ester coupling reaction is very similar. There is a difference of 0.02 ppm between the signals due to the alkyne protons of the phenol and benzoic acid monomers, so deconvolution of these signals was used to determine the loading of each monomer on the template in the attach step (see Fig. S7 and S8, and Table S1 of the ESI for details†). The proportion of mutator monomer (*χ*_mutator_) found attached to the template in the pre-ZIP intermediate (0%, 38%, 54%, 75% or 100%) is similar to the proportion present in the reaction mixture (0%, 30%, 50%, 70% or 100%) in each of the five experiments.

**Fig. 6:**
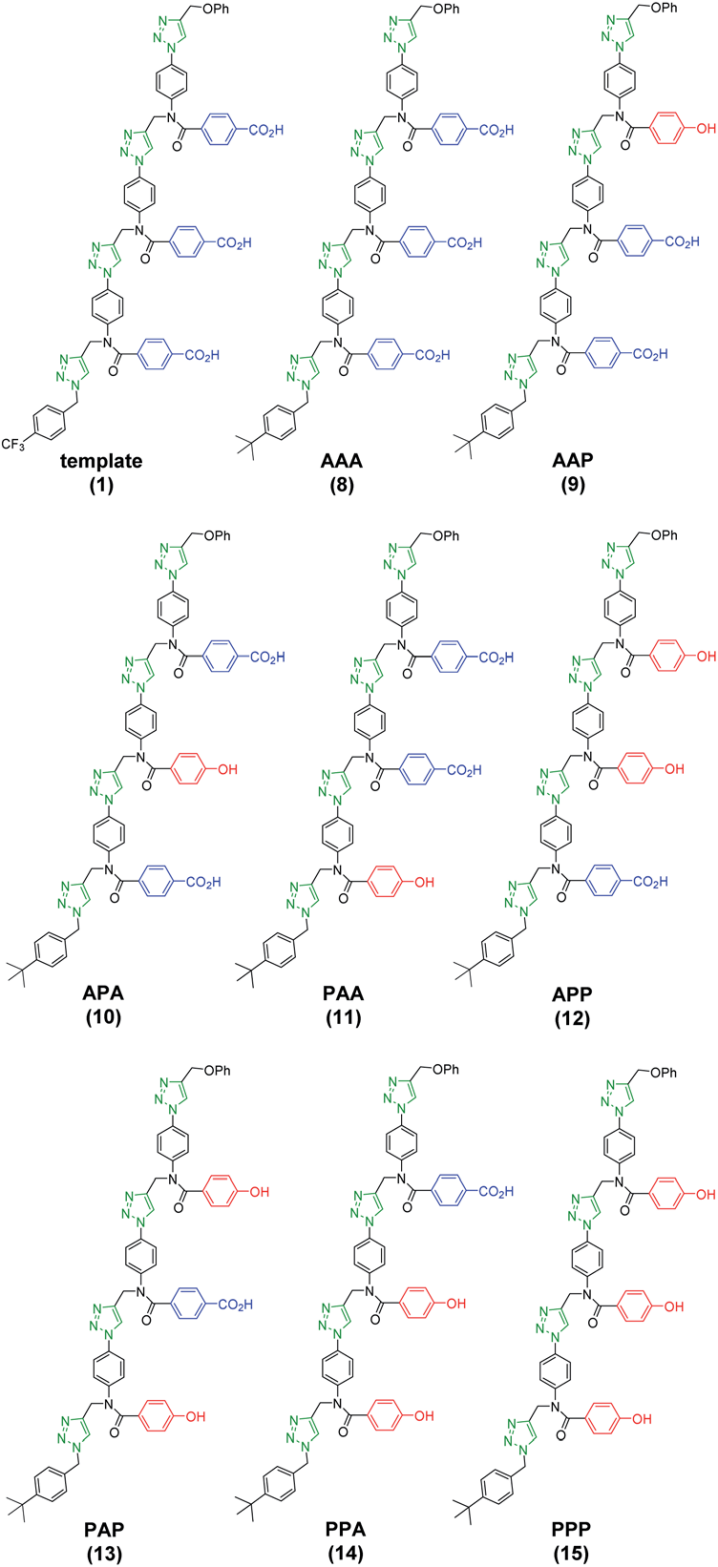
Molecular structure of all possible product sequences after covalent template-directed replication of template **1** using a mixture of monomers **4** and **7**. Sequences are written from the benzyl terminus using a 2-letter code: A for benzoic acid and P for phenol.

For each of the five different mixtures of pre-ZIP intermediates, the CuAAC ZIP reaction was then carried out in the presence of an excess of 4-*t*-butylbenzyl azide, which blocks intermolecular reactions between two different oligomers and prevents intramolecular macrocyclization reactions on the template.^24,25^ After washing with petroleum ether to remove excess capping azide, phenyl propargyl ether was used to cap the unreacted terminal azide groups of the daughter strands. The resulting mixture of duplexes was cleaved using basic hydrolysis to release the traceless linkers and regenerate the template **1** along with the templated products **8–15** (Fig. 6).

**Fig. 7:**
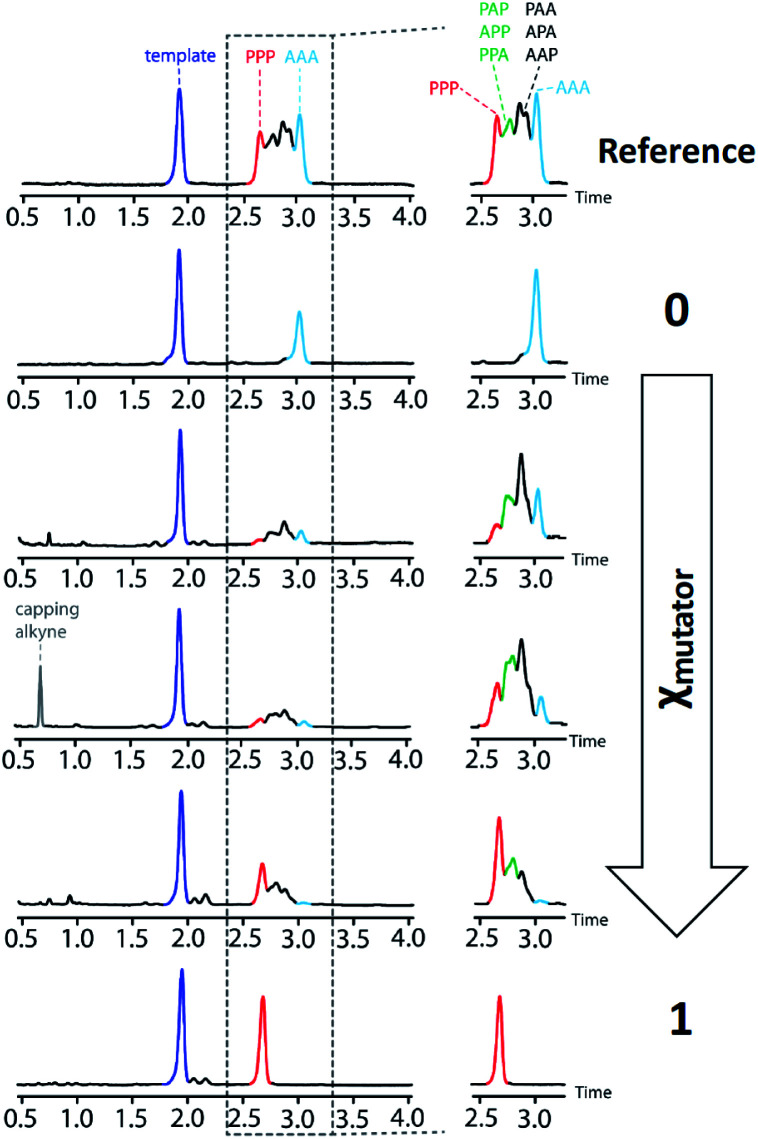
UPLC chromatograms from a cycle of covalent template-directed replication and mutation of the chemical information encoded in template **1** using different amounts of mutator **7** in the attach step (*χ*_mutator_). The top trace was created as a reference by mixing equimolar amounts of pure samples all eight product sequences (**8–15**) with template **1**. Experimental details are provided in the ESI.†

The product distributions from the replication cycle shown in Fig. 5 were determined using UPLC (Fig. 7). When the replication cycle was carried out using only monomer **4** in the attach step, a direct copy of the template (AAA, **8**) was obtained as the only product (Fig. 7, *χ*_mutator_ = 0). When only monomer **7** was used in the attach step, a reciprocal copy of the template (PPP, **15**) was obtained as the only product (Fig. 7, *χ*_mutator_ = 1). When mixtures of the two monomers were used, a mixture of product sequences was obtained, and the UPLC traces in Fig. 7 show clearly that the product distribution is directly related to the amount of mutator monomer **7** used in the attach step.

**Fig. 8:**
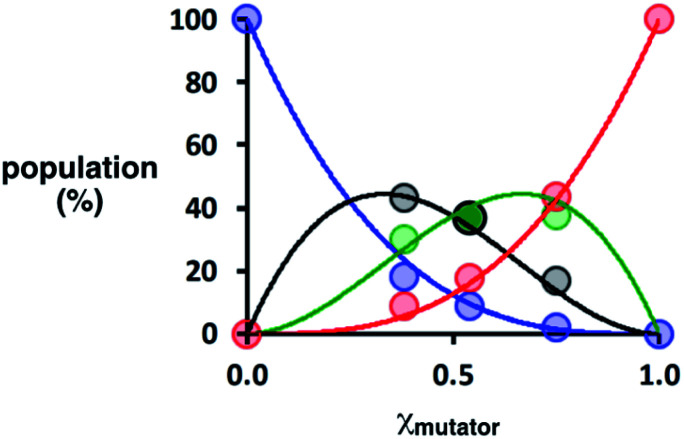
Theoretical (lines) and experimental (dots) product distributions for covalent template-directed replication of template **1** in the presence of different amounts of mutator monomer **7** (*χ*_mutator_). The total populations of isomeric sequences are plotted as a single point: the sum of the overlapped peaks corresponding to PAA, APA and AAP in black, and the sum of PPA, PAP and APP in green. The population of the direct copy AAA is shown in blue, and the reciprocal copy PPP in red.

Deconvolution of the UPLC peaks was used to quantify the product distribution (see ESI for details†). For reference purposes, each of the eight possible product sequences shown in Fig. 6 was synthesized independently (see ESI for details†). The UPLC trace labelled “Reference” in Fig. 7 shows an equimolar mixture of each of these oligomers and template **1**. We were not able to fully resolve the three isomeric sequences containing two phenols and one benzoic acid (PAP, APP and PPA), so the yield of these oligomers was determined using the total area of the peak corresponding to all three isomers. Similarly, the three sequences with one phenol and two benzoic acids (PAA, APA and AAP) were not fully resolved, so these peaks were combined to determine the total yield of the three isomeric oligomers. Fig. 8 compares the experimental results with the theoretical product distribution calculated assuming statistical incorporation of the two different monomers. The agreement indicates that none of the steps in the replication process are strongly dependent on the difference between the chemical structures of the two monomers. Thus the mutation rate in the replication process can be directly determined by choosing an appropriate monomer composition used in the attach step.

## Conclusions

These experiments demonstrate that the use of covalent base-pairing strategies for the replication of sequence information encoded in synthetic oligomers opens up new opportunities that are not available with the non-covalent base-pairs used in biology. More complex information transfer processes are possible by manipulation of the chemistry used to connect the two components of a covalent base-pair. Here, isosteric covalent base-pairs with traceless linkers have been used to introduce mutation in the replication of an oligotriazole, where information is encoded as the sequence of benzoic acid and phenol monomer units. When a benzoic acid–benzoic acid base-pairing system was used, a direct copy of a benzoic acid homo-oligomer template was obtained. When a phenol–benzoic acid base-pairing system was used, a reciprocal copy, the phenol homo-oligomer, was obtained. The two base-pairing systems are isosteric, which means that they can be used interchangeably, allowing direct and reciprocal copying to take place simultaneously on the same template strand. As a result, it is possible to introduce mutations in a replication process by spiking the monomer used for direct copying with the monomer used for reciprocal copying. We show that the mutation rate is determined precisely by the relative proportions of the two monomers. The ability to introduce mutation at a controlled rate is a key step in the development of synthetic systems capable of evolution, which requires replication with variation.

## Conflicts of interest

There are no conflicts to declare.

## Supplementary Material

SC-012-D0SC06770A-s001
