# Supplementary material for: Controlled mutation in the replication of synthetic oligomers
Source: Chem Sci. 2021 Feb 5;12(11):4063–8. doi: 10.1039/d0sc06770a (PMC8179503; doi:10.1039/d0sc06770a)
Supplement: SC-012-D0SC06770A-s001 [file SC-012-D0SC06770A-s001.pdf]

Electronic Supplementary Material (ESI) for Chemical Science.
This journal is © The Royal Society of Chemistry 2021

Supporting information

Controlled mutation in the replication of synthetic oligomers.

Diego Núñez-Villanueva, Christopher A. Hunter*

Department of Chemistry, University of Cambridge, Lensfield Road, Cambridge CB2 1EW, UK. E-mail: herchelsmith.orgchem@ch.cam.ac.uk

TABLE OF CONTENTS **Page**

General experimental details. S2

Synthesis of building blocks. S3

Reference 3-mer oligomers S12

Synthesis of APA 3-mer (10). S15

Synthesis of PAA 3-mer (11). S25

Synthesis of PPP 3-mer (15). S32

Covalent template-directed mutation of chemical information encoded in template 1. S36

References S73

General experimental details.

All the reagents and materials used in the synthesis of the compounds described below were bought from commercial sources, without prior purification. Dry THF and CH_2Cl_2 were obtained from a solvent purification system (Pure Solv™, Innovative Technology, Inc.). Anhydrous DMF was purchased from Sigma-Aldrich. Thin layer chromatography was carried out using with silica gel 60F (Merck) on glass plates. Flash chromatography was carried out on an automated system (Combiflash Rf+ or Combiflash Rf Lumen) using prepacked cartridges of silica (25µ PuriFlash® columns). All NMR spectroscopy was carried out on a Bruker 400 MHz DPX400, 400 MHz AVIII400, 500 MHz DCH cryoprobe or 500 MHz TCI Cryoprobe spectrometer using the residual solvent as the internal standard. All chemical shifts (δ) are quoted in ppm and coupling constants given in Hz. Splitting patterns are given as follows: s (singlet), bs (broad singlet), d (doublet), t (triplet), q (quadruplet), m (multiplet). FT-IR spectra were measured on a Bruker Alpha spectrometer equipped with an ATR cell. Melting points were measured in a Mettler Toledo MP50 Melting Point System. UPLC analysis of samples was performed using Waters Acquity H-class UPLC coupled with a single quadrupole Waters SQD2. Acquity UPLC CSH C18 column, 130 Å, 1.7 µm, 2.1 mm x 50 mm or Acquity UPLC BEH C8 column, 130 Å, 1.7 µm, 2.1 mm x 50 mm were used as UPLC columns. The conditions of the UPLC method are as follows: gradients of water + 0.1% formic acid (solvent A) and acetonitrile + 0.1% formic acid (solvent B) as specified in each case. Flow rate: 0.6 ml/min; Column temperature of 40°C; Injection volume of 2 µL. The signal was monitored at 254 nm. HRMS analysis was performed in a Waters LCT Premier equipped with a TOF mass analyser and W optics for enhanced resolution, using 50% aqueous acetonitrile with 0.25% formic acid as mobile phase.

Synthesis of building blocks.

Synthesis of 1-mer 4.

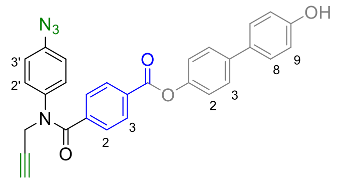

Compound **2**[S1] (0.108 g, 0.34 mmol), 4,4'-dihydroxybiphenyl (0.314 g, 1.68 mmol), EDC (0.097 g, 0.51 mmol) and DMAP (0.006 g, 0.05 mmol) were mixed in a round-bottom flask and dissolved in a mixture of dry CH_2Cl_2 (5 mL) and dry DMF (0.5 mL). The reaction was stirred at room temperature for 2h. The reaction was quenched with 0.1 M HCl soln. and diluted with EtOAc. The organic layer was separated and washed with 0.1 M HCl soln. (2x), H_2O (1x) and brine (1x). The solution was dried with anhydrous $MgSO_4$, filtered and the solvents evaporated. The obtained residue was purified by flash chromatography (from 0% to 40% of EtOAc in Pet. Ether followed by a second column using a gradient from 0% to 4% of MeOH in CH_2Cl_2) to afford 1-mer **4** (0.078 g, 47%) as a foam, along with the corresponding disubstituted derivative (0.014 g, 11%)

^{1}H NMR (400 MHz, CDCl$_3$): δ_H = 8.05 (d, 2H, *J* = 8.5 Hz, 3-H), 7.56 (d, 2H, *J* = 8.5 Hz, 3-H, biph), 7.46 (m, 4H, 2-H and 8-H, biph), 7.22 (d, 2H, *J* = 8.5 Hz, 2-H, biph), 7.15 (d, 2H, *J* = 8.5 Hz, 2'-H), 6.94 (d, 2H, *J* = 8.5 Hz, 3'-H), 6.90 (d, 2H, *J* = 8.5 Hz, 9-H, biph), 4.87 (s, 1H, OH), 4.69 (d, 2H, *J* = 2.5 Hz, N-CH$_2$), 2.29 (t, 1H, *J* = 2.5 Hz, CH, alkyne).

^{13}C NMR (100.6 MHz, CDCl$_3$): δ_C = 169.4 (CO, amide), 164.7 (CO, ester), 155.7 (10-C, biph), 149.7 (1-C, biph), 140.0 (1-C), 139.8 (4'-C), 139.0 (4-C, biph), 138.7 (1'-C), 132.8 (7-C, biph), 130.8 (4-C), 130.0 (3-C), 129.4 (2'-C), 128.9 (2-C), 128.4 (8-C, biph), 127.9 (3-C, biph), 121.9 (2-C, biph), 120.1 (3'-C), 115.9 (9-C, biph), 78.5 (C, alkyne), 73.1 (CH, alkyne), 39.9 (N-CH$_2$).

HRMS (ES+): calcd for $C_{29}H_{21}N_4O_4$ 489.1563 $[M+H]^+$, found 489.1562 $[M+H]^+$.

FT-IR (ATR): 3288, 2921, 2851, 2125, 2114, 1734, 1645, 1505, 1497, 1296 and 1203 ν_{max}/cm^{-1}.

^{1}H-NMR (400 MHz, $CDCl_3$) compound 4.

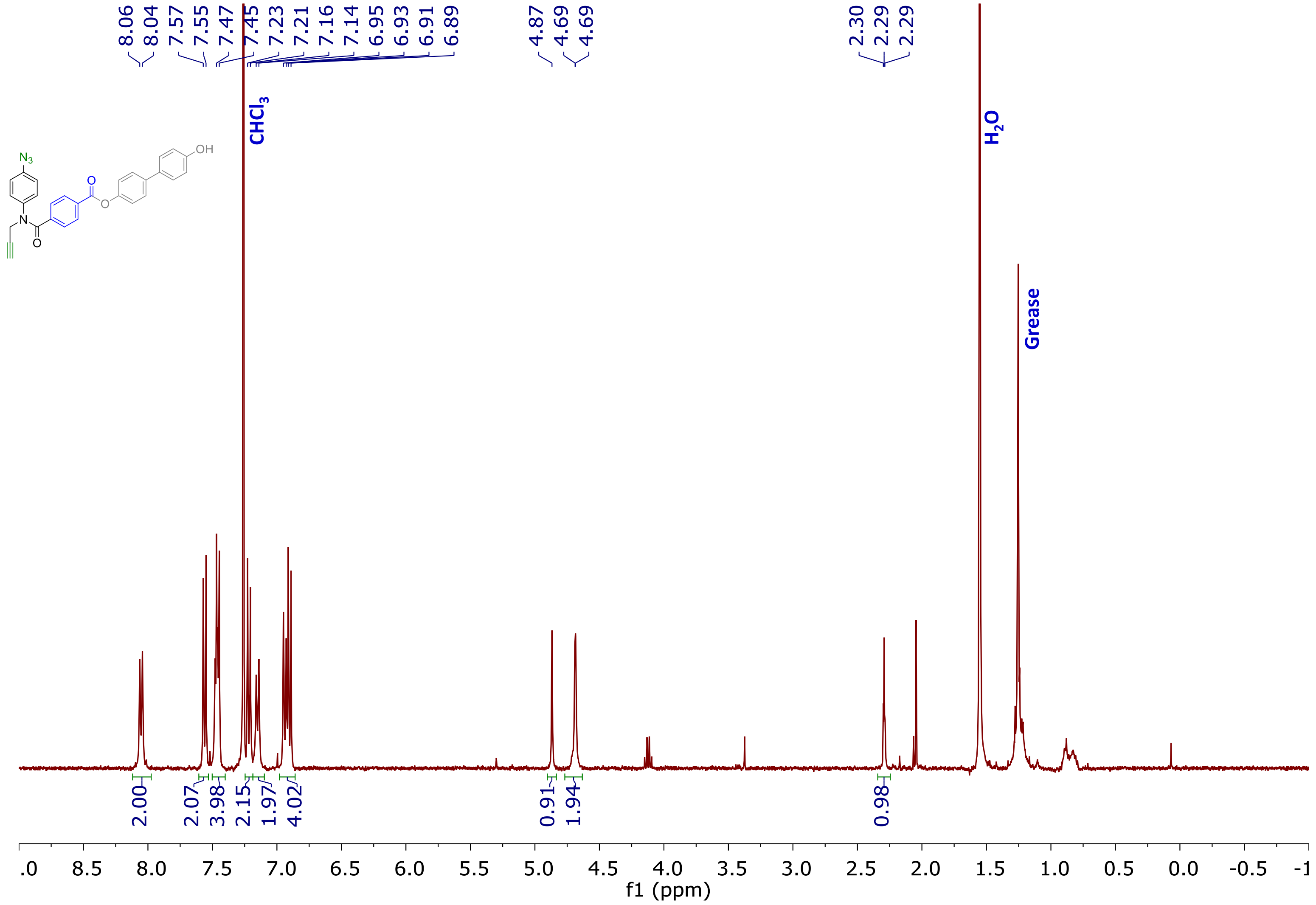

^{13}C-NMR (100.6 MHz, $CDCl_3$) compound 4.

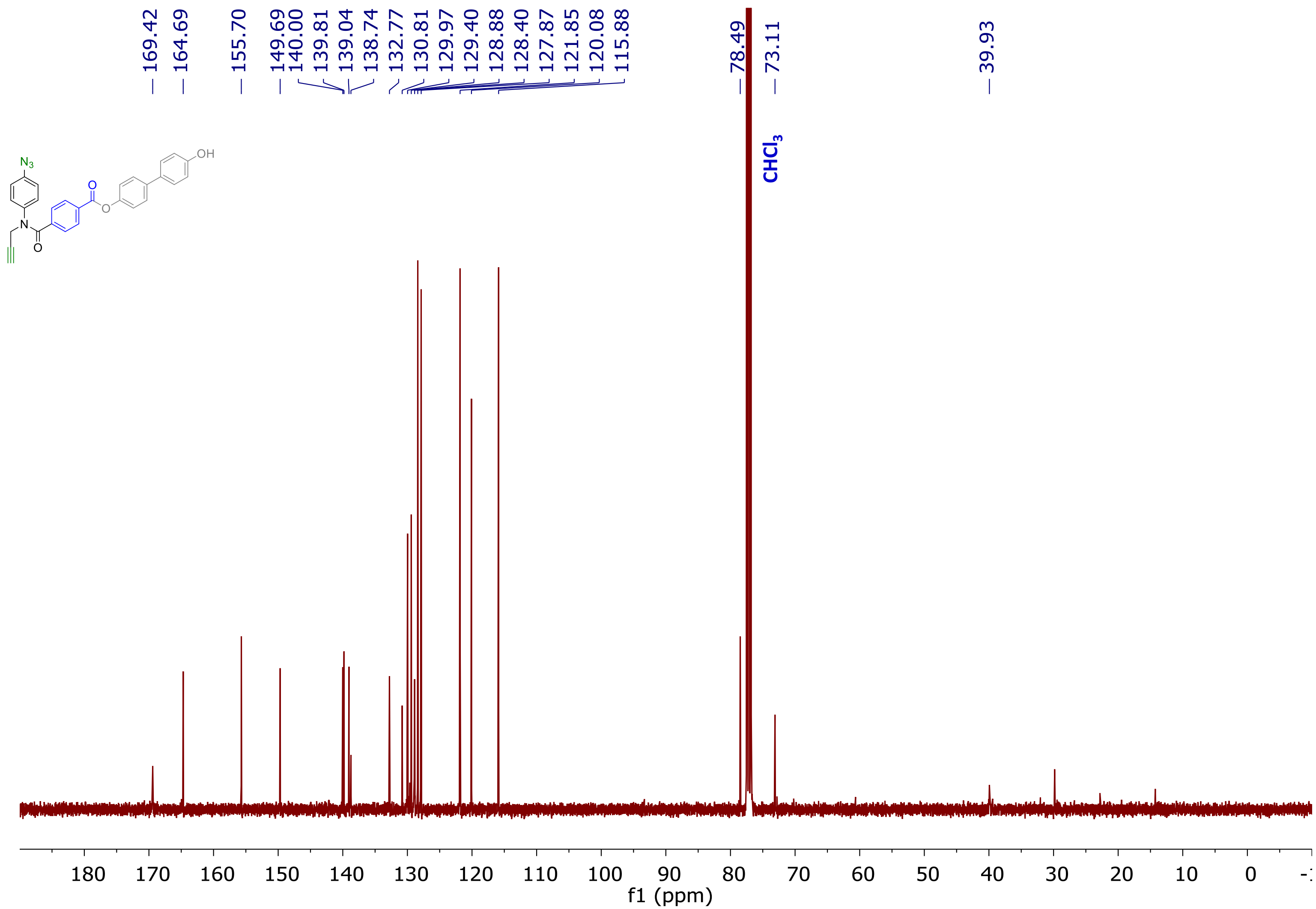

Synthesis of compound 6

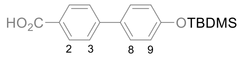

4'-hydroxy-4-biphenylcarboxylic acid (0.500 g, 2.34 mmol) was dissolved in DMF (10 mL) and treated with imidazole (1.41 g, 9.34 mmol) and TBDMS-Cl (0.795 g, 11.68 mmol). The reaction was stirred at room temperature for 16 h. The reaction was diluted with H_2O/EtOAc, washed with 5% aq. soln. LiCl (3x), H_2O (1x) and brine. The organic layer was dried over $MgSO_4$ and evaporated to dryness. The crude material (0.784 g, quant.) was used without further purification. The analytical and spectroscopic data match those previously reported in the literature.[S2,S3]

^{1}H NMR (400 MHz, CDCl$_3$): δ_H = 8.15 (d, 2H, *J* = 8.0 Hz, 2-H), 7.66 (d, 2H, *J* = 8.0 Hz, 3-H), 7.53 (d, 2H, *J* = 8.0 Hz, 8-H), 6.94 (d, 2H, *J* = 8.0 Hz, 9-H), 1.01 (s, 9H, ^{t}Bu, TBDMS), 0.24 (s, 6H, CH_3, TBDMS).

^{13}C NMR (100.6 MHz, CDCl$_3$): δ_C = 171.3 (CO), 156.2 (10-C), 146.2 (4-C), 132.8 (7-C), 130.7 (2-C), 128.4 (8-C), 127.3 (1-C), 126.6 (3-C), 120.6 (9-C), 25.7 (CH_3, ^{t}Bu, TBDMS), 18.3 (C, ^{t}Bu, TBDMS), -4.4 (CH_3, TBDMS).

FT-IR (ATR): 3295, 2920, 2850, 2104, 2094, 1733, 1603, 1505, 1268, 1185 and 1068 ν_{max}/cm^{-1}.

^{1}H-NMR (400 MHz, $CDCl_3$) compound 6.

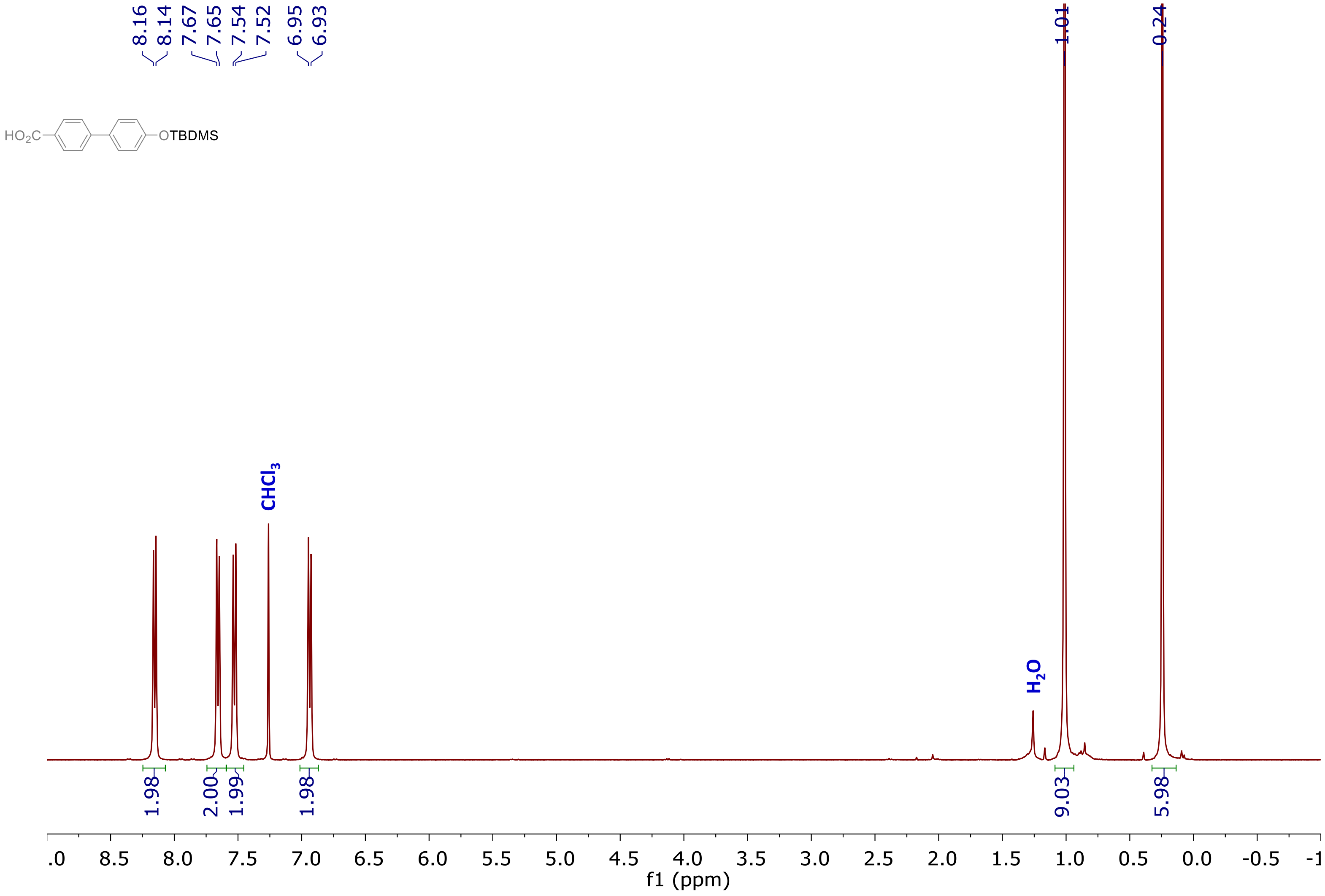

^{13}C-NMR (100.6 MHz, $CDCl_3$) compound 6.

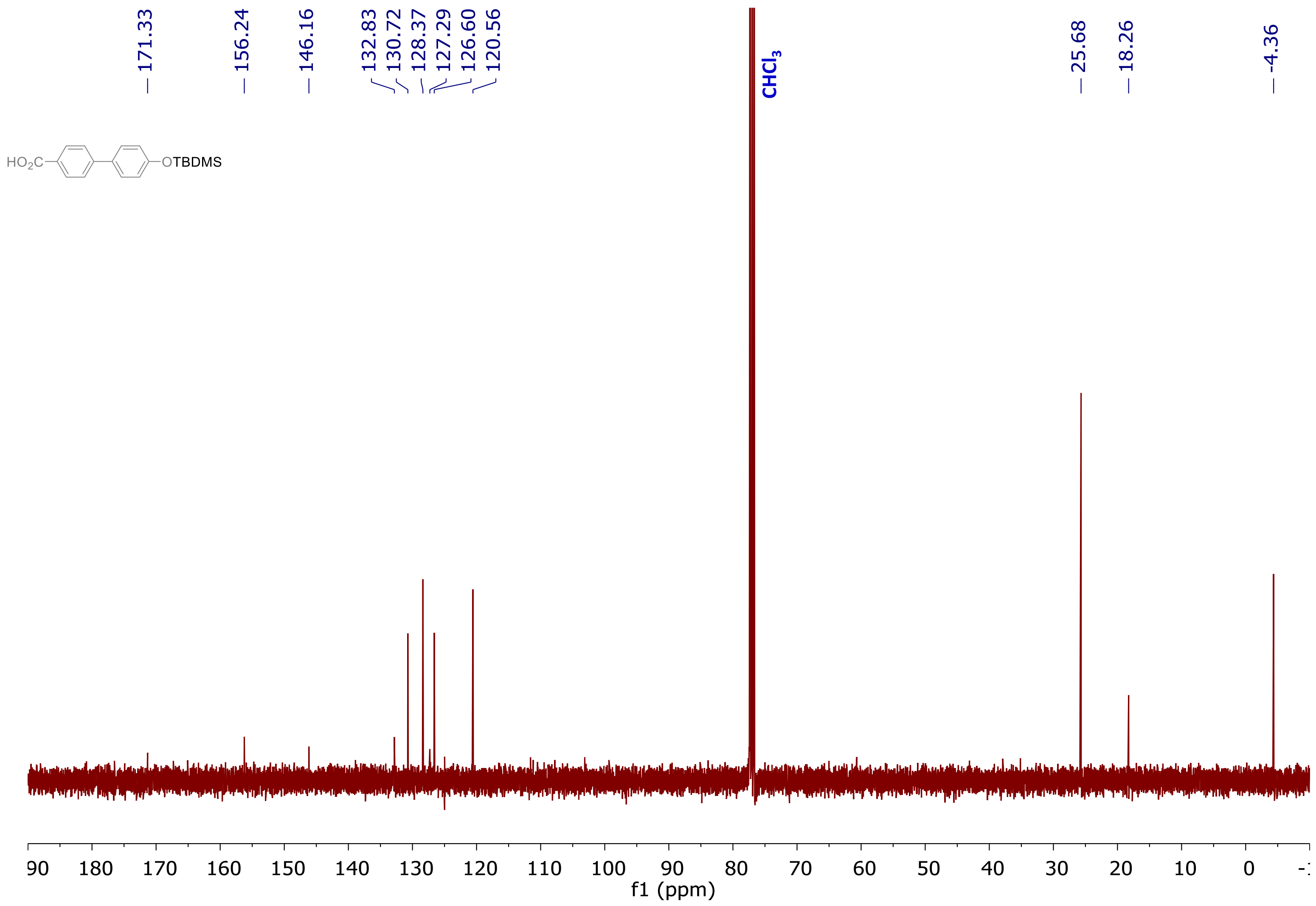

Synthesis of mutant 1-mer 7.

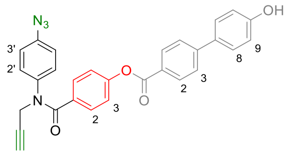

Compound **5**[S1] (0.100 g, 0.34 mmol), compound **6** (0.112 g, 0.34 mmol), EDC (0.098 g, 0.51 mmol) and DMAP (0.021 g, 0.17 mmol) were mixed in a round-bottom flask and dissolved in dry CH_2Cl_2 (5 mL). The reaction was stirred at room temperature for 1h and then TBAF (1M in THF, 0.69 mL, 0.69 mmol) was added dropwise. After 30 minutes of stirring, the reaction was quenched with 0.1 M HCl soln. and diluted with EtOAc. The organic layer was separated and washed with 0.1 M HCl soln. (2x), H_2O (1x) and brine (1x). The solution was dried with anhydrous $MgSO_4$, filtered and the solvents evaporated. The obtained residue was purified by flash chromatography (from 0% to 40% of EtOAc in Pet. Ether) to afford compound **7** (0.119 g, 72%) as a foam.

^{1}H NMR (400 MHz, CDCl$_3$): δ_H = 8.17 (d, 2H, *J* = 8.5 Hz, 2-H, biph), 7.66 (d, 2H, *J* = 8.5 Hz, 3-H, biph), 7.54 (d, 2H, *J* = 8.5 Hz, 8-H, biph), 7.44 (d, 2H, *J* = 8.5 Hz, 2-H), 7.16 (d, 2H, *J* = 8.5 Hz, 2'-H), 7.10 (d, 2H, *J* = 8.5 Hz, 3-H), 6.96 (d, 2H, *J* = 8.5 Hz, 3'-H), 6.93 (d, 2H, *J* = 8.5 Hz, 9-H, biph), 5.26 (s, 1H, OH), 4.67 (d, 2H, *J* = 2.5 Hz, N-CH$_2$), 2.28 (t, 1H, *J* = 2.5 Hz, CH, alkyne).

^{13}C NMR (100.6 MHz, CDCl$_3$): δ_C = 169.4 (CO, amide), 164.7 (CO, ester), 156.4 (10-C, biph), 152.4 (4-C), 146.3 (4-C, biph), 139.5 and 139.4 (1'C and 4'-C), 132.4 and 132.4 (1-C and 1-C, biph), 130.9 (2-C, biph), 130.6 (2-C), 129.3 (2'-C), 128.8 (8-C, biph), 127.3 (7-C), 126.8 (3-C, biph), 121.5 (3-C), 120.1 (3'-C), 116.1 (9-C, biph), 78.9 (C, alkyne), 72.8 (CH, alkyne), 40.2 (N-CH$_2$).

HRMS (ES+): calcd for $C_{29}H_{21}N_4O_4$ 489.1563 [M+H]$^+$, found 489.1553 [M+H]$^+$.

FT-IR (ATR): 3295, 2920, 2850, 2104, 2094, 1733, 1603, 1505, 1268, 1185 and 1068 ν_{max}/cm^{-1}.

^{1}H-NMR (400 MHz, $CDCl_3$) compound 7.

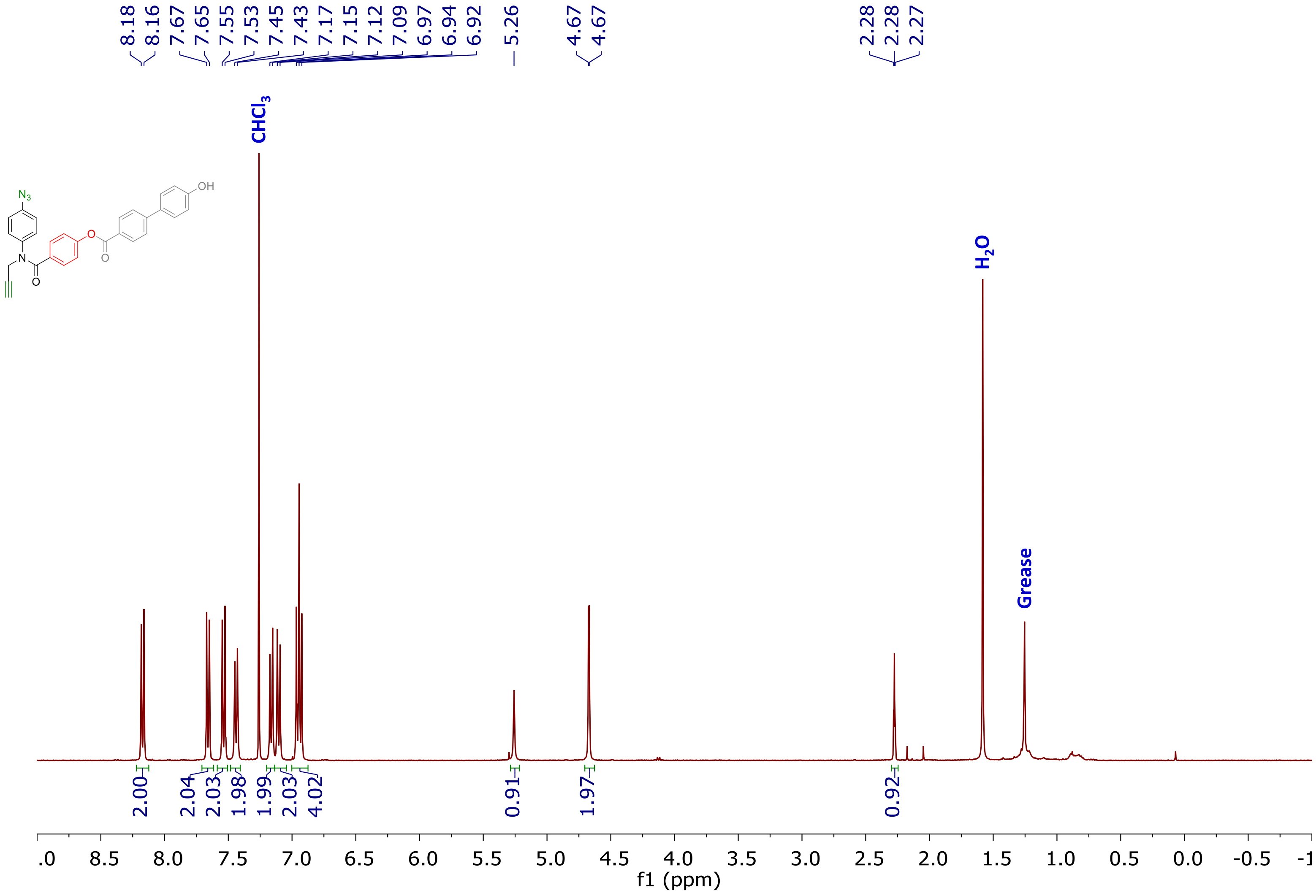

^{13}C-NMR (100.6 MHz, $CDCl_3$) compound 7.

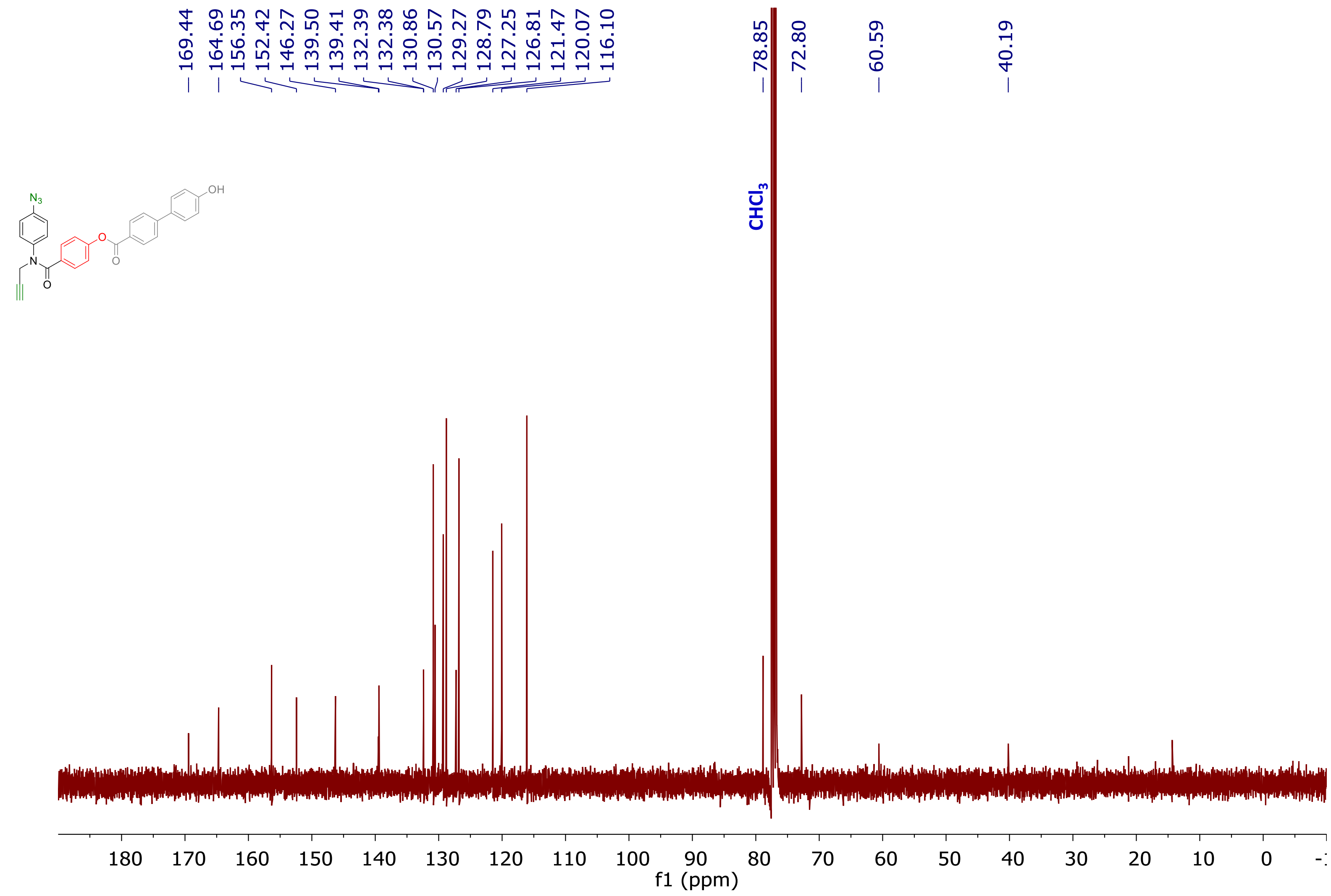

Reference 3-mer oligomers

Scheme S1 shows the chemical structure of template **1** and all the possible sequences of phenol and benzoic acid information units in a 3-mer oligomer (**8**-**15**). The template (**1**) bears a *p*-trifluoromethylbenzyl terminal group in contrast to the *p*-*tert*-butylbenzyl groups of oligomers **8**-**15** to provide a spectroscopic handle to distinguish the template **1** and its identical copy **8**. Sequences are written from the *tert*-butylbenzyl or *p*-trifluoromethylbenzyl terminus using a 2-letter code: A for benzoic acid and P for phenol.

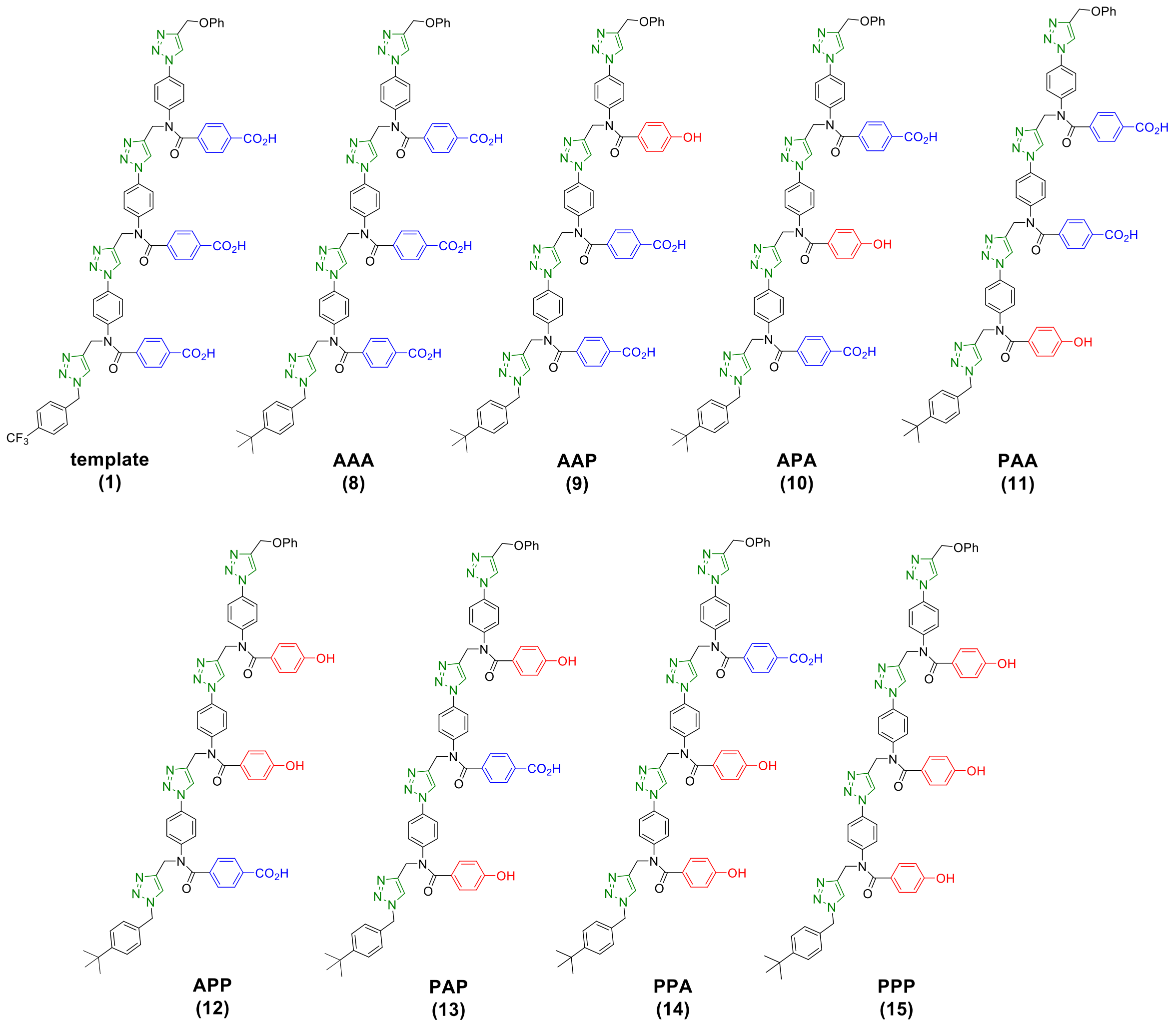

Scheme S1. Molecular structure of template and all the possible product sequences from the covalent template-directed synthesis using template **1** and 1-mers **4** and **7**.

Synthesis and characterization of reference 3-mer oligomers.

Template (1).

This compound has been previously described.[S4]

AAA (8).

This compound has been previously described.[S4]

AAP (9).

This compound has been previously described.[S1]

APA (10).

Synthesis and characterization of this compound is provided in the next section.

PAA (11).

Synthesis and characterization of this compound is provided in the next section.

APP (12).

This compound has been previously described.[S1]

PAP (13).

This compound has been previously described.[S1]

PPA (14).

This compound has been previously described.[S1]

PPP (15).

Synthesis and characterization of this compound is provided in the next section.

UPLC traces for reference 3-mer oligomers

Fig. S1 shows the individual UPLC traces for template **1** and the sequences **8**-**15**. The bottom two chromatograms correspond to two mixtures prepared as reference for the covalent template-directed mutation experiments.

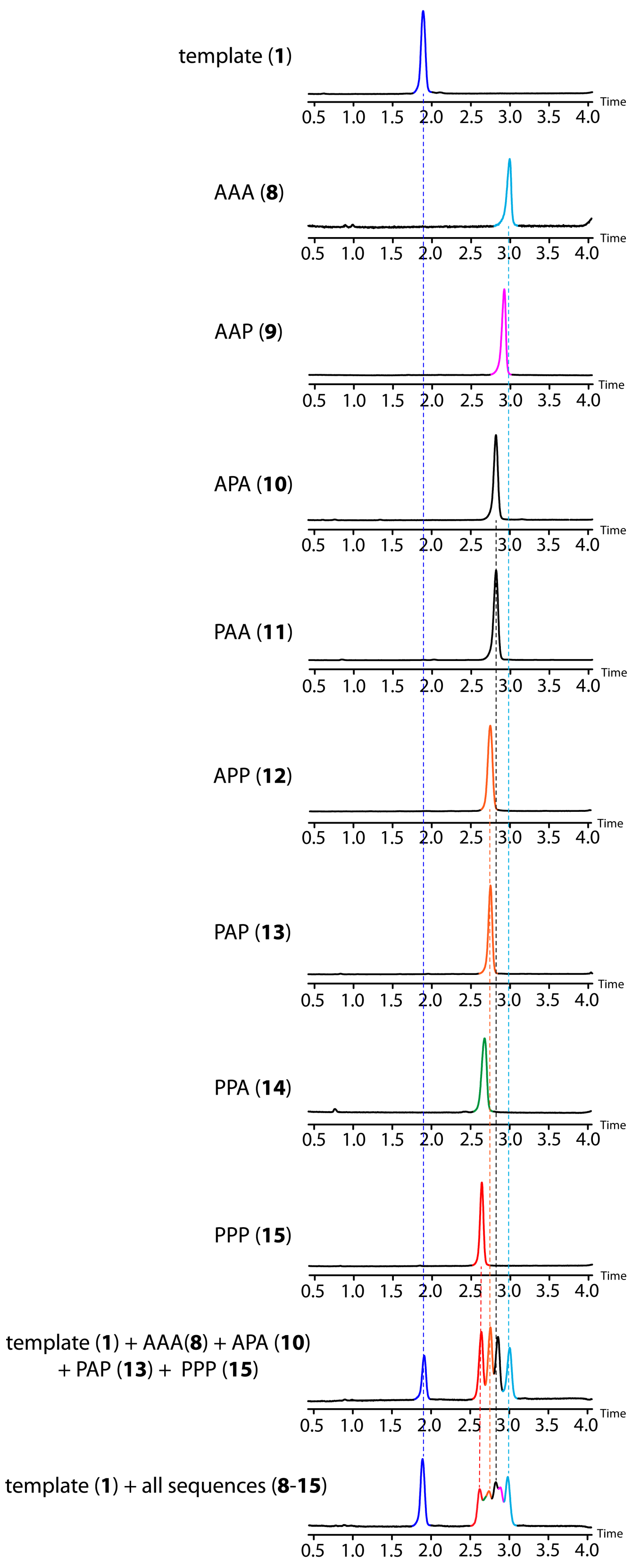

Fig S1. UPLC traces for template **1** and the sequences **8-15**. The bottom two chromatograms correspond to two mixtures prepared as reference for the covalent template-directed mutation experiments. *Conditions*: C18 column at 40 °C (254 nm) using water + 0.1% formic acid (A) and CH_3CN + 0.1% formic acid (B) with flow rate of 0.6 ml/min; Gradient of 0-4 minutes from 45% to 50% B + 1 minute 100% B.

Synthesis of APA 3-mer (10).

Scheme S2 shows the synthetic route towards APA 3-mer oligomer **10**. Copper-catalysed azide alkyne cycloaddition (CuAAC) reaction between compound **S1**[S5] and **S2**[S1] followed by TBAF-mediate removal of silyl protecting groups afforded 2-mer **S3** in moderate yield. Subsequent CuAAC reaction of **S3** with protected 1-mer **S4**[S1] followed by alkyne deprotection using TBAF provided 3-mer **S5** in good yield. Final step toward 3-mer **10** involved capping of **S5** with *tert*-butyl benzyl azide followed by basic hydrolysis of methyl ester groups to afford 3-mer **10** in good yield.

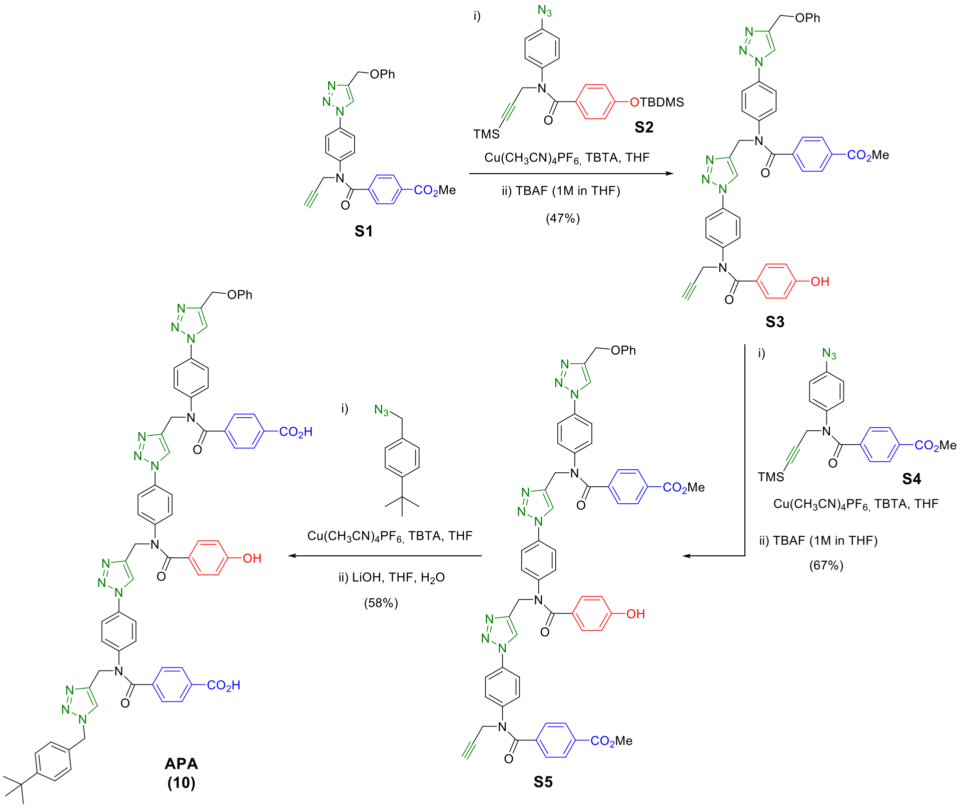

Scheme S2. Synthesis of APA 3-mer oligomer (**10**).

Synthesis of compound S3.

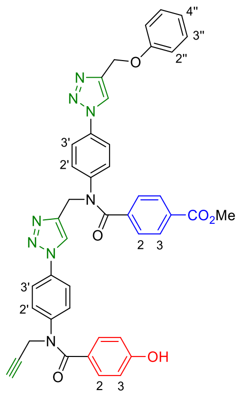

Compound **S1**[S5] (0.018 g, 0.04 mmol), compound **S2**[S1] (0.018 g, 0.04 mmol), $Cu(CH_3CN)_4PF_6$ (1.4 mg, 0.004 mmol) and TBTA (2.1 mg, 0.004 mmol) were mixed in a round-bottom flask and, under N_2, THF (5 mL) was added. The reaction was stirred overnight at room temperature. Once the reaction was completed, TBAF (1 M in THF, 0.08 mL, 0.08 mmol) was added dropwise. The solution was stirred for 10 min at room temperature, quenched with 0.1 M HCl and extracted with EtOAc (3x) followed by washing with H_2O (1x) and brine (1x). The organic layer was dried over anhydrous $MgSO_4$ and concentrate under vacuum. The crude was purified by flash column chromatography on silica gel (gradient from 10% to 70% of EtOAc in Pet. Ether) to afford compound **S3** (0.014 g, 47%) as a foam.

^{1}H NMR (400 MHz, CDCl$_3$): δ_H = 8.17 (s, 1H, CH$_{triaz}$, internal), 7.98 (s, 1H, CH$_{triaz}$, PhO cap), 7.84 (d, 2H, *J* = 8.0 Hz, 3-H, ester), 7.65 (d, 2H, *J* = 8.5 Hz, 3'-H, internal), 7.58 (d, 2H, *J* = 8.5 Hz, 3'-H, PhO cap), 7.37 (bs, 1H, OH), 7.36 (d, 2H, *J* = 8.0 Hz, 2-H, ester), 7.29-7.24 (m, 6H, 2'-H and 3''-H), 7.22 (d, 2H, *J* = 8.5 Hz, 2-H, phenol), 6.99 (d, 2H, *J* = 7.5 Hz, 2''-H), 6.98 (t partially overlapped, 1H, *J* = 7.5 Hz, 4''-H), 6.61 (d, 2H, *J* = 8.5 Hz, 3-H, phenol), 5.26 (s, 2H, O-CH$_2$), 5.22 (s, 2H, N-CH$_2$, ester), 4.69 (d, 2H, *J* = 2.5 Hz, N-CH$_2$, phenol), 3.85 (s, 3H, O-CH$_3$), 2.25 (t, 1H, *J* = 2.5 Hz, alkyne).

^{13}C NMR (100.6 MHz, CDCl$_3$): δ_C = 170.1 (CO, amide phenol), 169.7 (CO, amide ester), 166.2 (CO, ester), 158.7 (4-C, phenol), 158.2 (1''-C), 145.5 (C$_{triaz}$, PhO cap), 144.3 (C$_{triaz}$, internal), 143.9 (1'-C, internal), 143.6 (1'-C, PhO cap), 139.1 (1-C, ester), 135.5 (4'-C, PhO cap), 135.2 (4'-C, internal), 131.8 (4-C, ester), 131.4 (2-C, phenol), 129.8, 129.5, 129.0, 128.9 and 128.8 (2-C and 3-C, ester; 2'-C; 3''-C), 126.1 (1-C, phenol), 122.3 (CH$_{triaz}$, internal), 121.6 (4''-C), 121.5 (3'-C, PhO cap), 121.3 (3'-C, internal), 120.9 (CH$_{triaz}$, PhO cap), 115.1 (3-C, phenol), 114.9 (2''-C), 78.9 (C, alkyne), 72.9 (CH, alkyne), 61.9 (O-CH$_2$), 52.5 (O-CH$_3$), 46.2 (N-CH$_2$, ester), 40.0 (N-CH$_2$, phenol).

HRMS (ES+): calcd for $C_{43}H_{35}N_8O_6$ 759.2680 $[M+H]^+$, found 759.2668 $[M+H]^+$.

FT-IR (ATR): ν_{max} 3294, 2919, 1719, 1644, 1518, 1279, 1237, 826 and 757 cm^{-1}.

^{1}H-NMR (400 MHz, $CDCl_3$) compound S3.

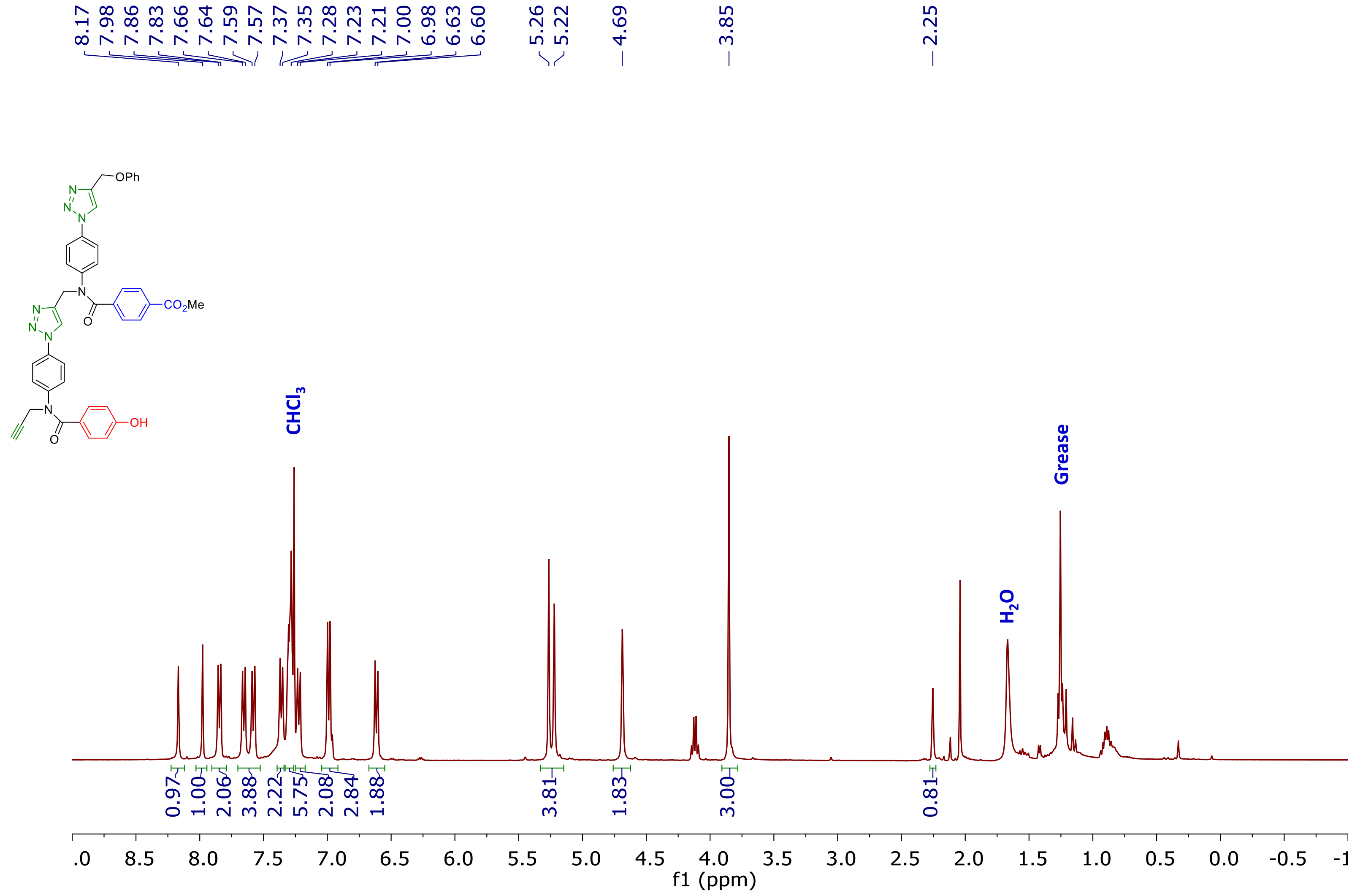

^{13}C-NMR (100.6 MHz, $CDCl_3$) compound S3.

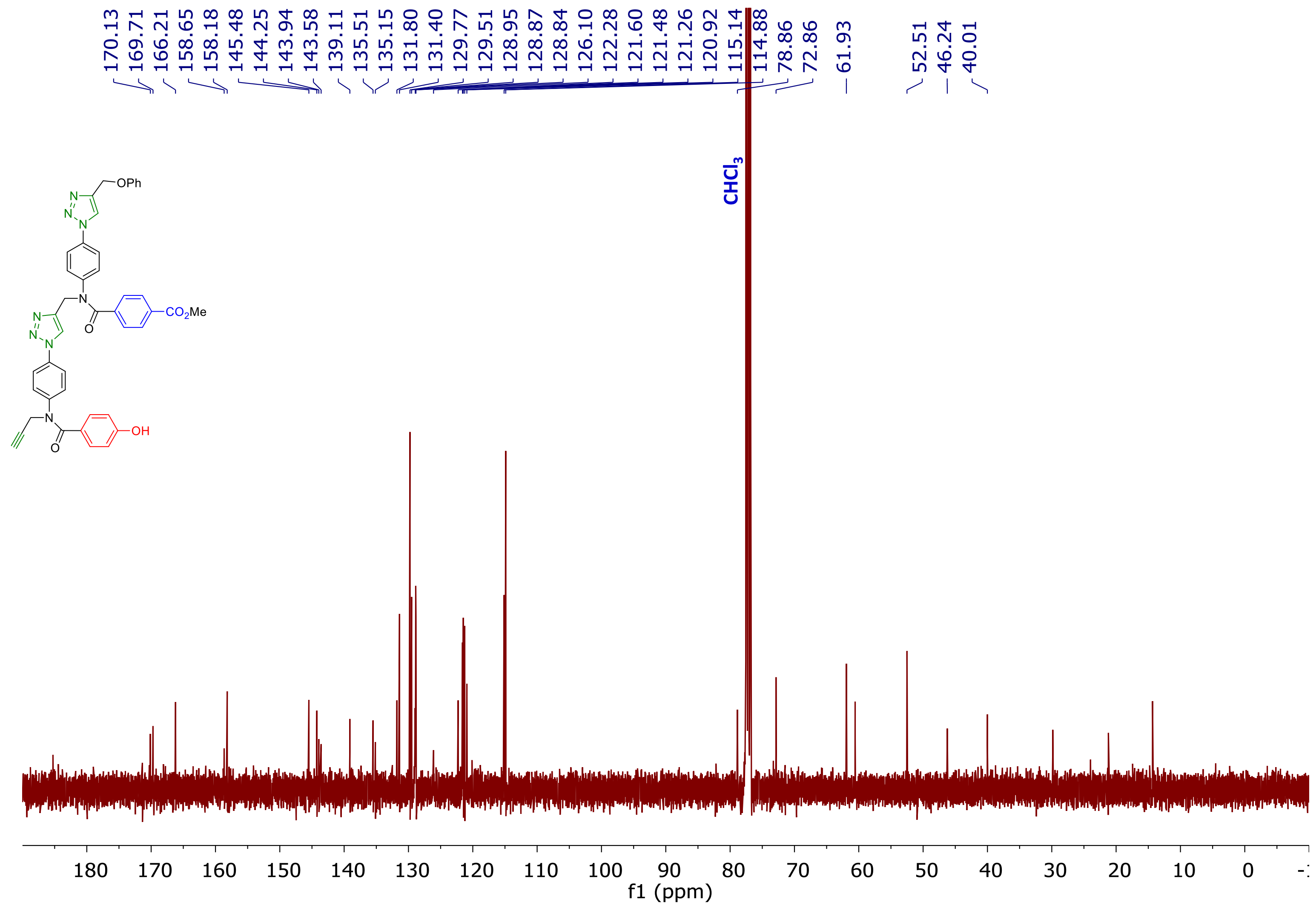

Synthesis of compound S5.

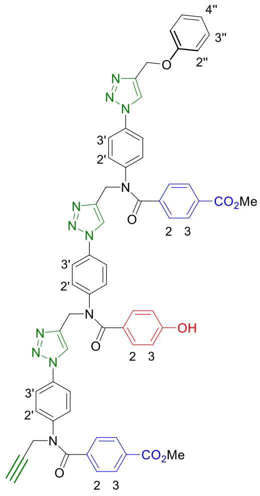

Compound **S3** (0.014 g, 0.02 mmol), compound **S4**[S1] (0.008 g, 0.02 mmol), $Cu(CH_3CN)_4PF_6$ (0.7 mg, 0.002 mmol) and TBTA (1.0 mg, 0.002 mmol) were mixed in a round-bottom flask and, under N_2, THF (2 mL) was added. The reaction was stirred overnight at room temperature. Once the reaction was completed, TBAF (1 M in THF, 0.02 mL, 0.02 mmol) was added dropwise. The solution was stirred for 10 min at room temperature, quenched with 0.1 M HCl and extracted with EtOAc (3x) followed by washing with H_2O (1x) and brine (1x). The organic layer was dried over anhydrous $MgSO_4$ and concentrate under vacuum. The crude was purified by flash column chromatography on silica gel (gradient from 10% to 85% of EtOAc in Pet. Ether) to afford compound **S5** (0.014 g, 67%) as a foam.

^{1}H NMR (400 MHz, CDCl$_3$): δ_H = 8.18 (s, 1H, CH$_{triaz}$, internal), 8.13 (s, 1H, CH$_{triaz}$, internal), 7.98 (s, 1H, CH$_{triaz}$, PhO cap), 7.87 (d, 2H, *J* = 8.0 Hz, 3-H, ester), 7.84 (d, 2H, *J* = 8.0 Hz, 3-H, ester), 7.64 (d, 2H, *J* = 8.5 Hz, 3'-H), 7.59 (d, 2H, *J* = 8.5 Hz, 3'-H), 7.58 (d, 2H, *J* = 8.5 Hz, 3'-H), 7.41 (d, 2H, *J* = 8.0 Hz, 2-H, ester), 7.36 (d, 2H, *J* = 8.0 Hz, 2-H, ester), 7.32-7.26 (m, 8H, 2'-H and 3''-H), 7.15 (d, 2H, *J* = 8.5 Hz, 2-H, phenol), 6.98 (d, 2H, *J* = 8.0 Hz, 2''-H), 6.97 (t partially overlapped, 1H, 4''-H), 6.58 (d, 2H, *J* = 8.5 Hz, 3-H, phenol), 5.26 (s, 2H, O-CH$_2$), 5.21 (s, 2H, N-CH$_2$), 5.17 (s, 2H, N-CH$_2$), 4.71 (d, 2H, *J* = 2.5 Hz, N-CH$_2$, alkyne), 3.85 (s, 6H, O-CH$_3$), 2.30 (t, 1H, *J* = 2.5 Hz, alkyne).

^{13}C NMR (100.6 MHz, CDCl$_3$): δ_C = 170.3 (CO, amide phenol), 169.9 and 169.4 (CO, amide ester), 166.3 and 166.2 (CO, ester), 158.8 (4-C, phenol), 158.2 (1''-C), 145.5 (C$_{triaz}$, PhO cap), 145.0 (1'-C, PhO cap), 144.8 and 144.2 (C$_{triaz}$), 143.6 and 142.5 (1'-C, internal), 139.2 and 139.1 (1-C, ester), 135.8 and 135.5 (4'-C, internal), 131.8 (4-C, ester), 131.4 (2-C, phenol), 129.8, 129.5, 129.2, 128.9, 128.8 and 128.7 (2-C and 3-C, ester; 2'-C; 3''-C), 126.2 (1-C, phenol), 122.2 (CH$_{triaz}$, internal), 121.6 (4''-C), 121.5, 121.3 and 121.2 (3'-C), 120.9 (CH$_{triaz}$, PhO cap), 115.2 (3-C, phenol), 114.9 (2''-C), 78.4 (C, alkyne), 73.3 (CH, alkyne), 61.9 (O-CH$_2$), 52.5 (O-CH$_3$), 46.5 and 46.1 (N-CH$_2$), 39.8 (N-CH$_2$, alkyne).

HRMS (ES+): calcd for $C_{61}H_{49}N_{12}O_9$ 1093.3745 [M+H]$^+$, found 1093.3730 [M+H]$^+$.

FT-IR (ATR): ν_{max} 3139, 2951, 2922, 1719, 1643, 1517, 1277, 1238, 845 and 751 cm^{-1}.

^{1}H-NMR (400 MHz, $CDCl_3$) compound S5.

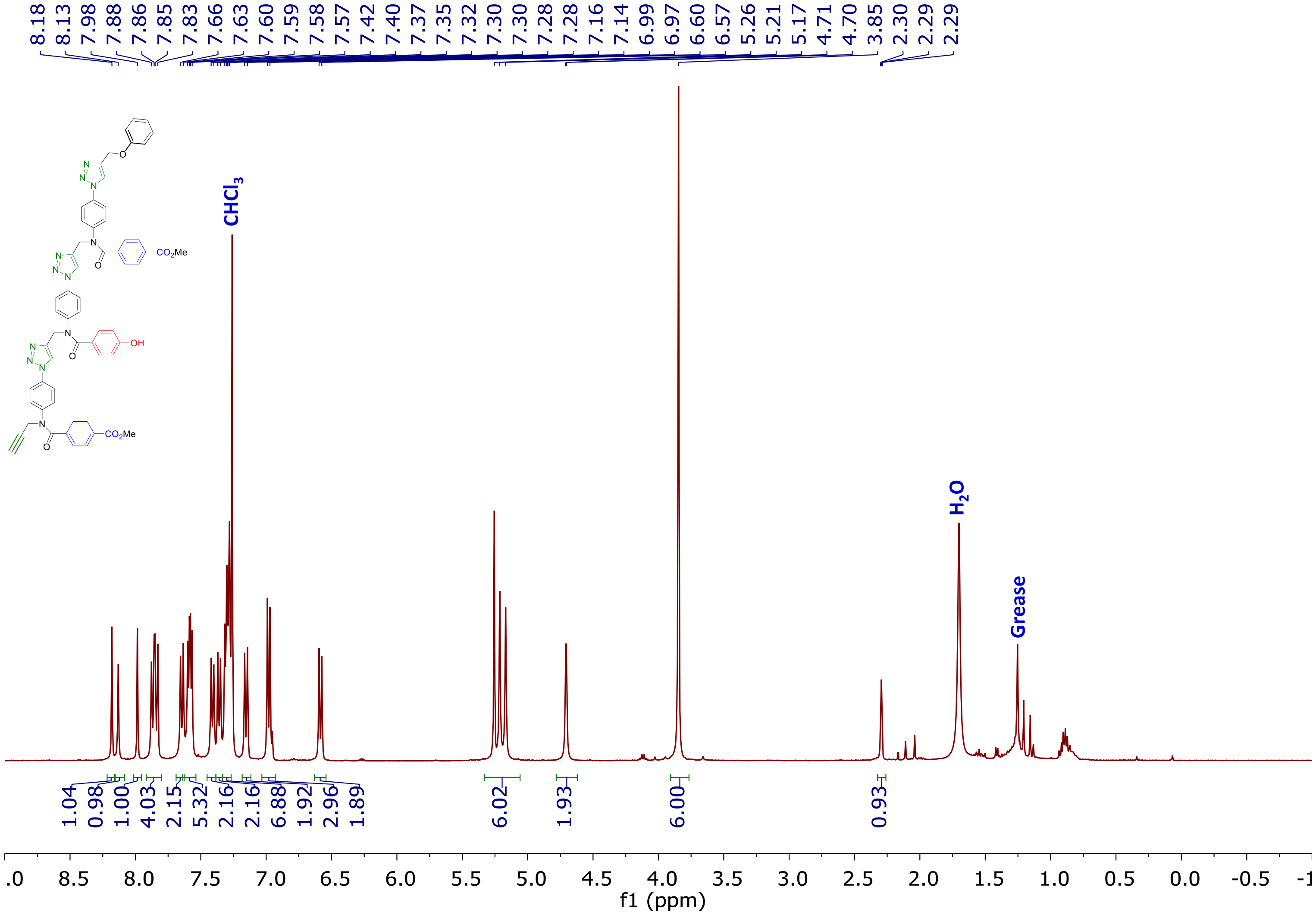

^{13}C-NMR (100.6 MHz, $CDCl_3$) compound S5.

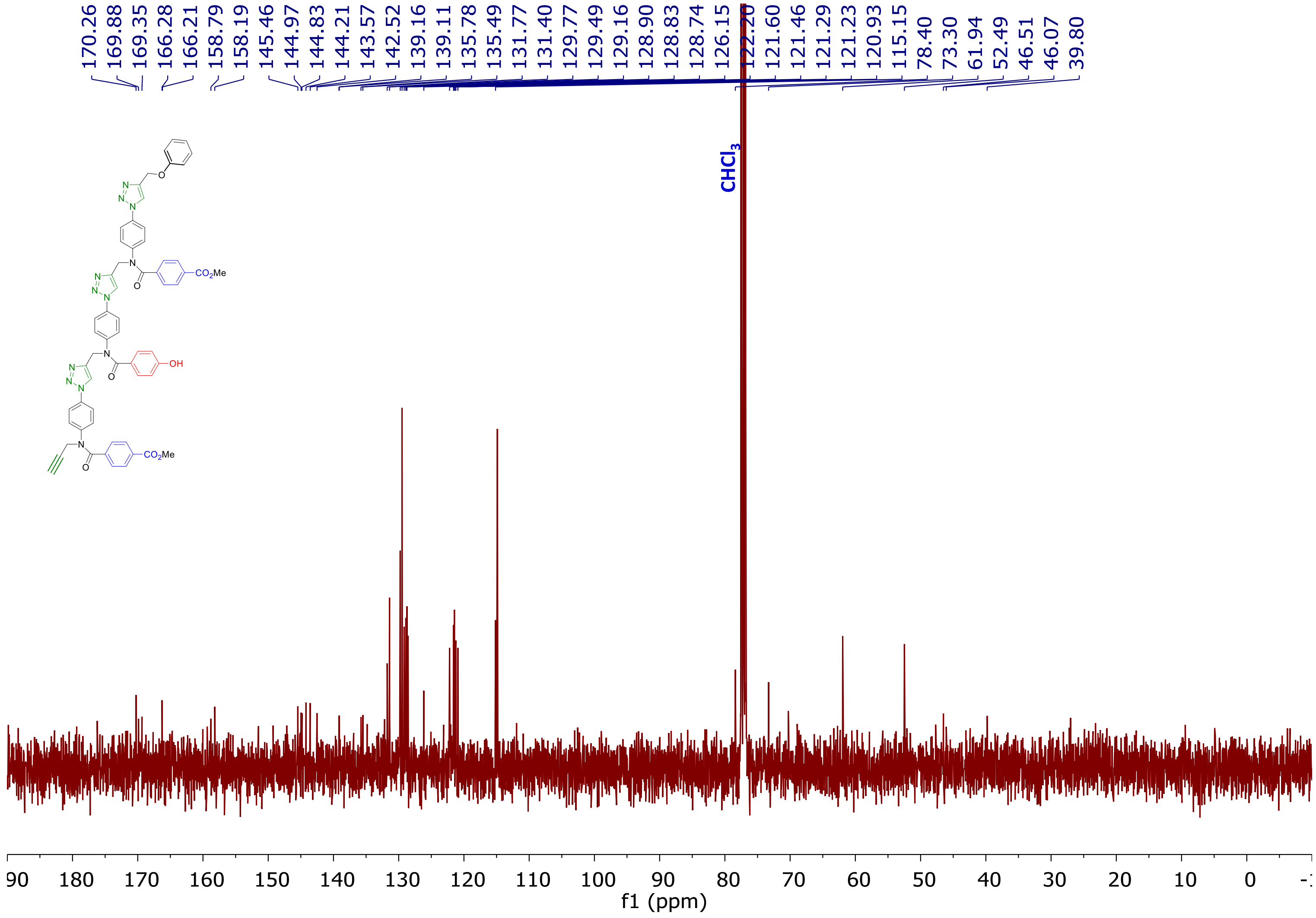

Synthesis of APA 3-mer (10).

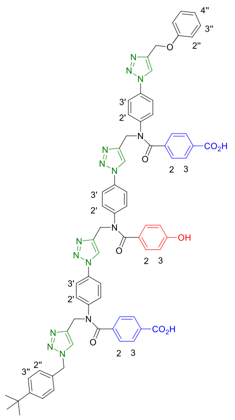

Compound **S5** (0.011 g, 0.01 mmol), 1-(azidomethyl)-4-*tert*-butylbenzene (3 mg, 0.02 mmol), $Cu(CH_3CN)_4PF_6$ (0.3 mg, 0.001 mmol) and TBTA (0.5 mg, 0.001 mmol) were mixed in a round-bottom flask and, under N_2, THF (3 mL) was added. The reaction was stirred overnight at room temperature. Then, H_2O was added (1.5 mL) followed by LiOH (2.5 mg, 0.06 mmol) and the reaction was stirred at room temperature for 2h. Then, the crude was diluted with H_2O and acidified with 1 M HCl soln. to pH 2-3. The aqueous phase was extracted with EtOAc (3x) and the combined organic phase was washed with EDTA soln. (2x), H_2O (1x) and brine (1x), dried over $MgSO_4$ and evaporated to dryness. The residue was purified by flash chromatography on silica gel (gradient from 0% to 20% of MeOH in CH_2Cl_2) to afford 3-mer **10** (0.007 g, 58%) as a white powder.

^{1}H NMR (500 MHz, DMSO-*d*$_6$): δ_H = 8.87 (s, 1H, CH_{triaz}, PhO cap), 8.73 (s, 1H, $CH_{triaz,}$), 8.70 (s, 2H, CH_{triaz}), 8.10 (s, 1H, CH_{triaz}, ^{t}Bu cap), 7.77 (m, 10H, 3-H, acid; 3'-H), 7.44 (d, 2H, *J* = 8.5 Hz, 2-H, acid), 7.40 (m, 6H, 2'-H), 7.33 (d, 2H, *J* = 8.5 Hz, 2-H, acid), 7.28 (m, 4H, 3''-H, PhO cap; 3''-H, ^{t}Bu cap), 7.21 (d, 2H, *J* = 8.5 Hz, 2-H, phenol), 7.07 (d, 2H, *J* = 8.5 Hz, 2''-H, ^{t}Bu cap), 7.03 (d, 2H, *J* = 8.0 Hz, 2''-H, PhO cap), 6.94 (t, 1H, *J* = 7.5 Hz, 4''-H, PhO cap), 6.59 (d, 2H, *J* = 8.5 Hz, 3-H, phenol), 5.49 (s, 2H, N-CH_2, ^{t}Bu cap), 5.20 (s, 2H, N-CH_2), 5.16 (bs, 6H, N-CH_2), 1.17 (s, 9H, ^{t}Bu).

^{13}C NMR (125 MHz, DMSO-*d*$_6$): δ_C = 169.3 (CO, amide phenol), 168.9 (CO, amide acid), 167.7 (CO, acid), 159.1 (4-C, phenol), 158.0 (1''-C, PhO cap), 150.5 (4''-C, ^{t}Bu cap), 144.7, 144.3 and 144.0 (C_{triaz}), 143.7 (1'-C), 143.0 (C_{triaz}), 142.8 and 142.4 (1'-C), 138.4 and 138.4 (1-C, acid), 134.6, 134.6 and 134.2 (4-C, acid; 4'-C), 133.2 (1''-C, ^{t}Bu cap), 131.0 (2-C, phenol), 129.6, 129.2, 129.2, 128.9, 128.8, 128.8, 128.2 and 127.4 (C_{arom}), 125.6 (1-C, phenol), 125.4 (3''-C, ^{t}Bu cap), 124.2 (CH_{triaz}, ^{t}Bu cap), 122.9 (CH_{triaz}, PhO cap), 121.5 and 121.5 (CH_{triaz}), 121.8 (4''-C, PhO cap), 120.6, 120.5 and 120.3 (3'-C), 114.7 (2''-C, PhO cap), 114.5 (3-C, phenol), 60.9 (O-CH_2), 52.4 (N-CH_2, ^{t}Bu cap), 45.4, 45.4 and 44.9 (N-CH_2), 34.2 (C, ^{t}Bu), 31.0 (CH_3, ^{t}Bu).

HRMS (ES+): calcd for $C_{70}H_{60}N_{15}O_9$ 1254.4698 $[M+H]^+$, found 1254.4672 $[M+H]^+$.

FT-IR (ATR): ν_{max} 3369, 2958, 2923, 2850, 1641, 1599, 1517, 1385, 1234, 1045 and 845 cm^{-1}.

^{1}H-NMR (500 MHz, DMSO-d_6) compound 10.

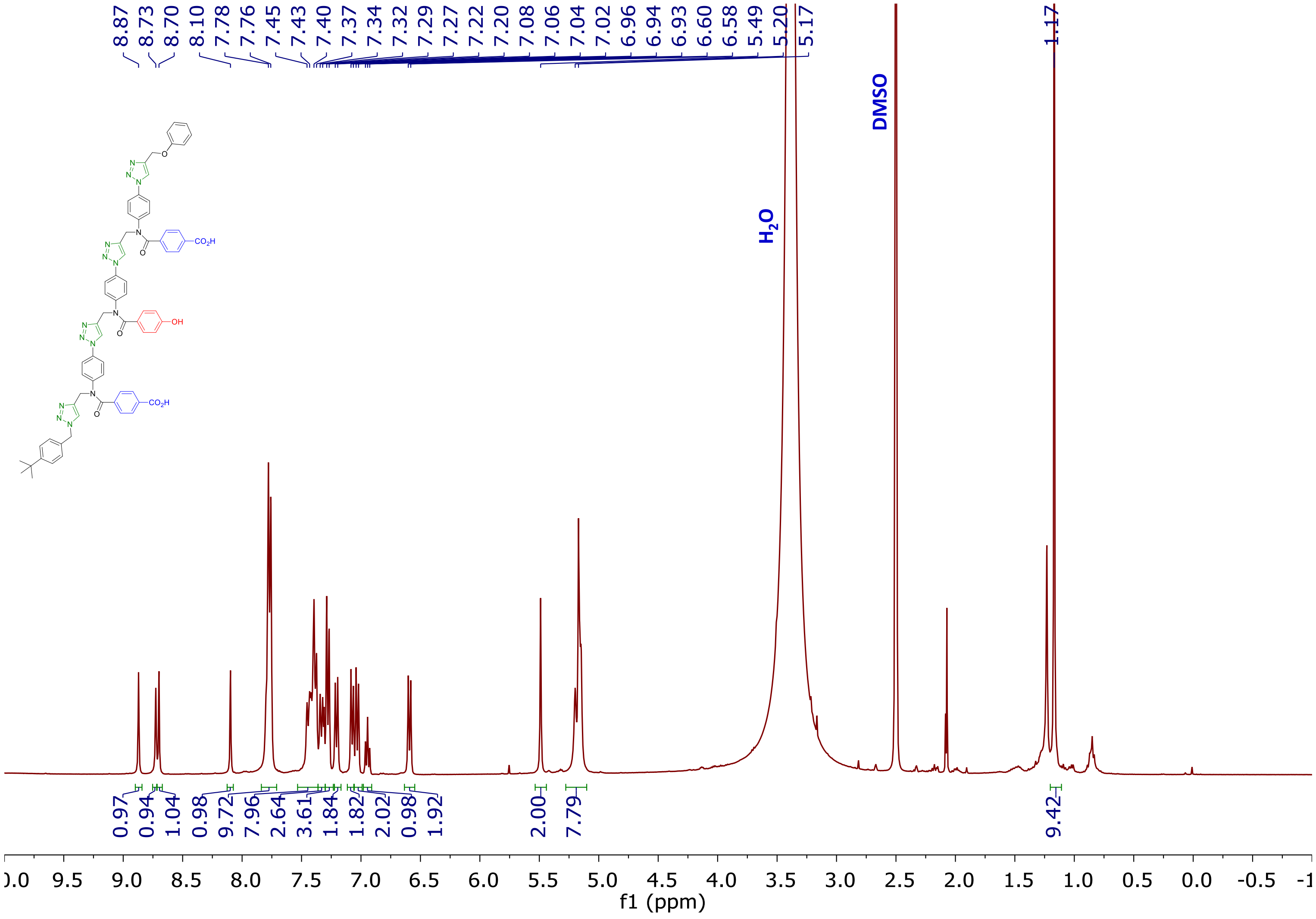

^{13}C-NMR (125 MHz, DMSO-d_6) compound 10.

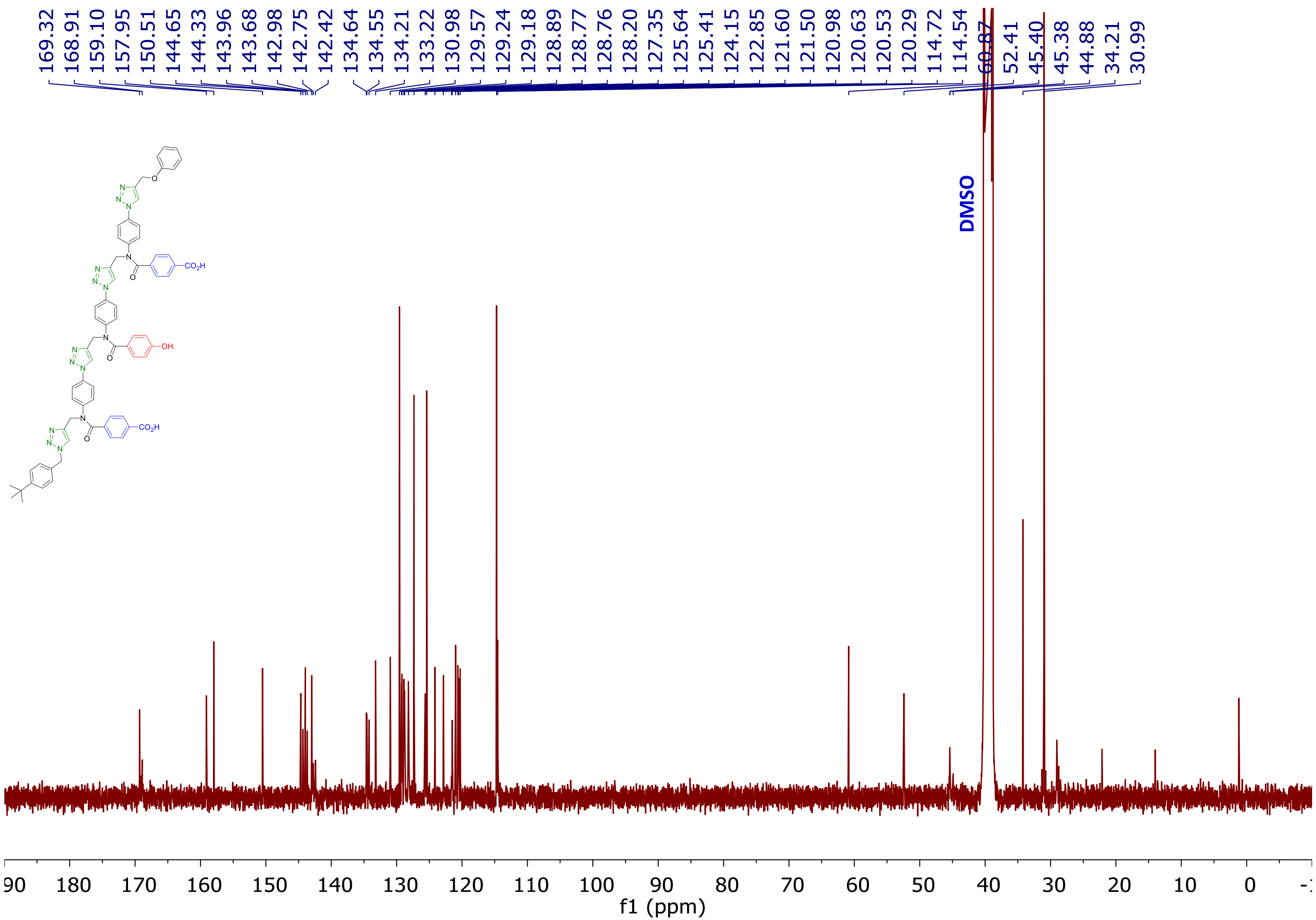

Synthesis of PAA 3-mer (11).

Scheme S3 shows the synthetic route towards PAA 3-mer oligomer **11**. CuAAC reaction between compound **S6**[S5] and **S2**[S1] followed by TBAF-mediate removal of silyl protecting groups afforded 3-mer **S7** in excellent yield. Capping of **S7** with *tert*-butyl benzyl azide followed by basic hydrolysis of methyl ester groups gave access to 3-mer **11** in good yield.

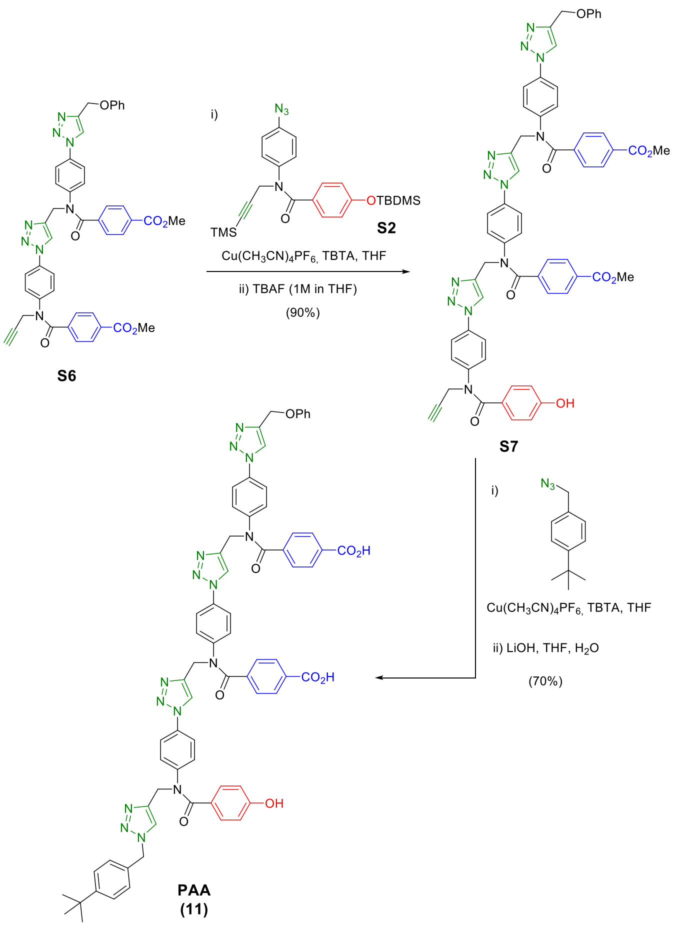

Scheme S3. Synthesis of PAA 3-mer oligomer (**11**).

Synthesis of compound S7.

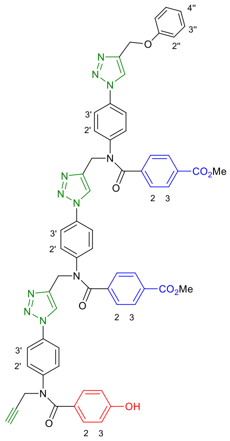

Compound **S6**[S5] (0.020 g, 0.03 mmol), compound **S2**[S1] (0.012 g, 0.03 mmol), $Cu(CH_3CN)_4PF_6$ (0.9 mg, 0.003 mmol) and TBTA (1.3 mg, 0.003 mmol) were mixed in a round-bottom flask and, under N_2, THF (5 mL) was added. The reaction was stirred overnight at room temperature. Once the reaction was completed, TBAF (1 M in THF, 0.05 mL, 0.05 mmol) was added dropwise. The solution was stirred for 10 min at room temperature, quenched with 0.1 M HCl soln. and extracted with EtOAc (3x) followed by washing with H_2O and brine. The organic layer was dried over anhydrous $MgSO_4$ and concentrate under vacuum. The crude was purified by flash column chromatography on silica gel (gradient from 10% to 90% of EtOAc in Pet. Ether) to afford compound **S7** (0.025 g, 90%) as a foam.

^{1}H NMR (400 MHz, $CDCl_3$): δ_H = 8.13 (s, 2H, CH_{triaz}, internal), 7.98 (s, 1H, CH_{triaz}, PhO cap), 7.84 (m, 5H, 3-H, ester; OH), 7.64 (d, 2H, *J* = 8.5 Hz, 3'-H), 7.59 (m, 4H, *J* = 8.5 Hz, 3'-H), 7.36 (m, 4H, 2-H, ester), 7.32-7.24 (m, 8H, 2'-H and 3''-H), 7.20 (d, 2H, *J* = 8.5 Hz, 2-H, phenol), 6.98 (d, 2H, *J* = 7.5 Hz, 2''-H), 6.97 (t partially overlapped, 1H, 4''-H), 6.60 (d, 2H, *J* = 8.5 Hz, 3-H, phenol), 5.26 (s, 2H, O-CH_2), 5.24 (s, 2H, N-CH_2), 5.20 (s, 2H, N-CH_2), 4.68 (d, 2H, *J* = 2.5 Hz, N-CH_2, alkyne), 3.85 (s, 3H, O-CH_3), 3.84 (s, 3H, O-CH_3), 2.30 (t, 1H, *J* = 2.5 Hz, alkyne).

^{13}C NMR (100.6 MHz, $CDCl_3$): δ_C = 170.1 (CO, amide phenol), 169.7 and 169.7 (CO, amide ester), 166.2 and 166.2 (CO, ester), 158.8 (4-C, phenol), 158.2 (1''-C), 145.5 (C_{triaz}, PhO cap), 144.3 and 144.2 (C_{triaz}), 143.9, 143.6 and 143.3 (1'-C), 139.2 and 139.1 (1-C, ester), 135.5, 135.4 and 135.1 (4'-C,), 131.8 and 131.4 (4-C, ester), 129.8, 129.5, 129.0, 128.8 and 126.0 (2-C and 3-C, ester; 2'-C; 3''-C), 122.1 and 122.1 (CH_{triaz}, internal), 121.6, 121.4, 121.3 and 121.2 (3'-C and 4''-C) 120.9 (CH_{triaz}, PhO cap), 115.1 (3-C, phenol), 114.9 (2''-C), 78.9 (C, alkyne), 72.8 (CH, alkyne), 61.9 (O-CH_2), 52.5 (O-CH_3), 46.2 and 46.0 (N-CH_2), 40.0 (N-CH_2, alkyne).

HRMS (ES+): calcd for $C_{61}H_{49}N_{12}O_9$ 1093.3745 $[M+H]^+$, found 1093.3733 $[M+H]^+$.

FT-IR (ATR): ν_{max} 3153, 2237, 1720, 1643, 1606, 1518, 1279, 1239, 845 and 752 cm^{-1}.

^{1}H-NMR (400 MHz, $CDCl_3$) compound S7.

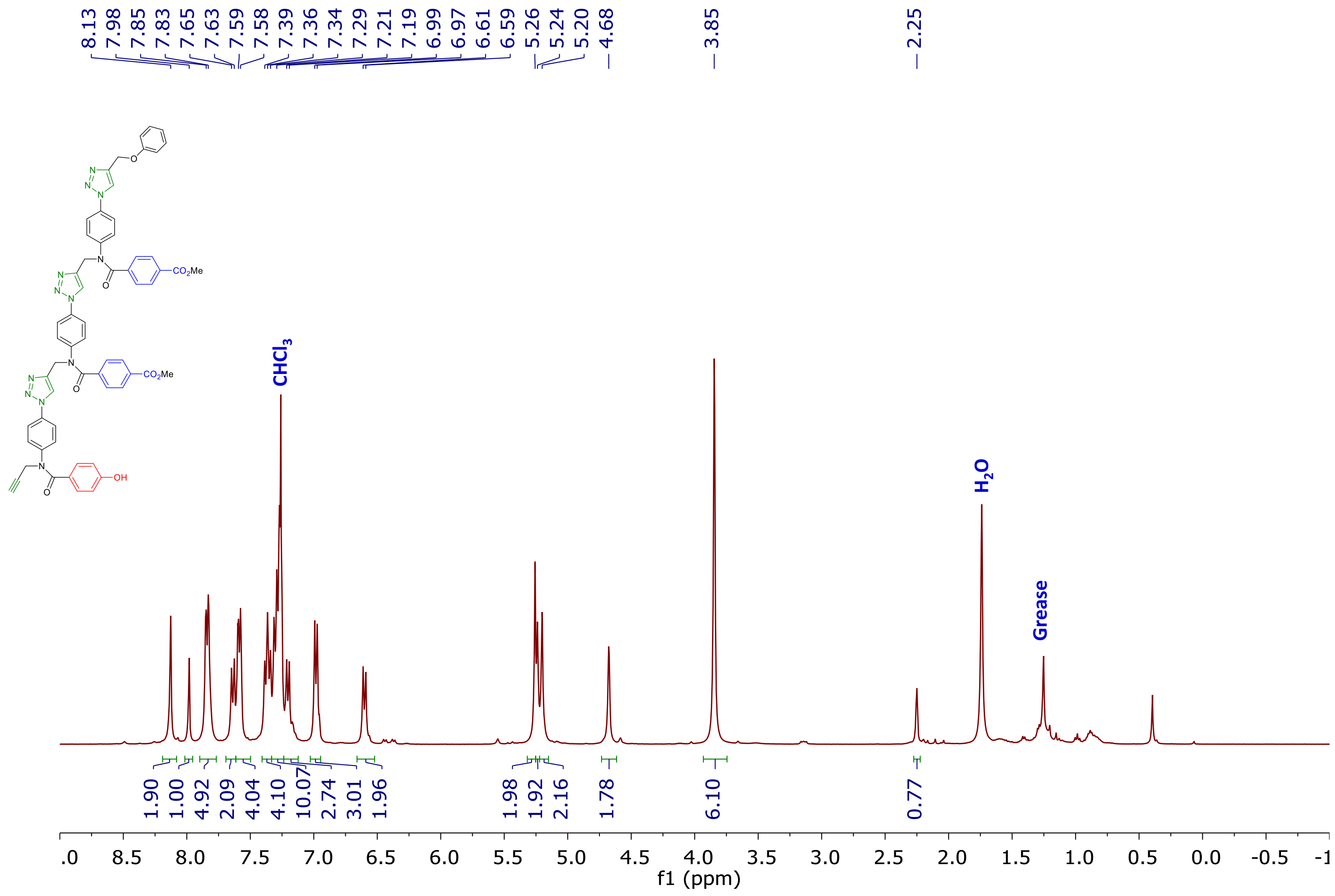

^{13}C-NMR (100.6 MHz, $CDCl_3$) compound S7.

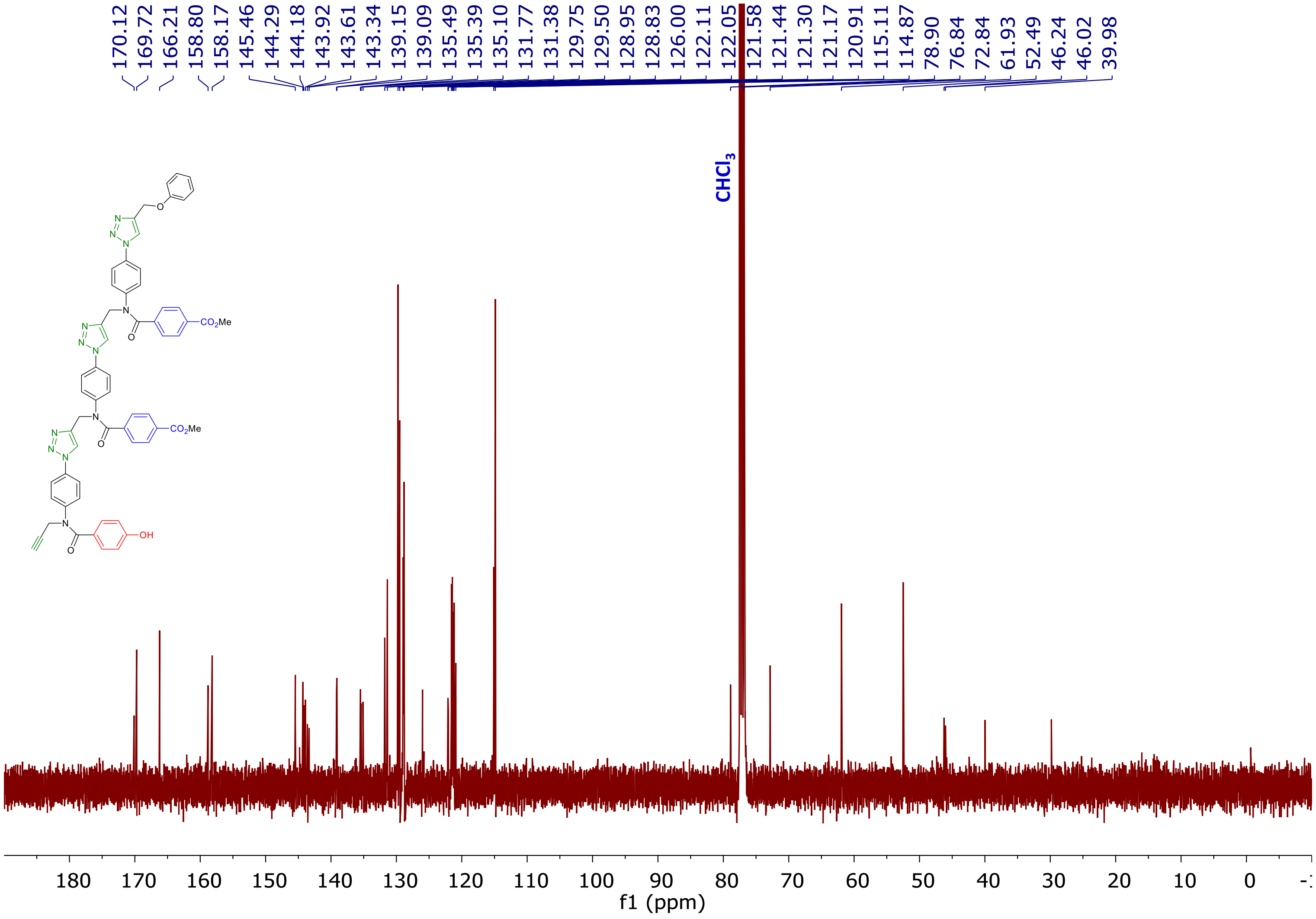

Synthesis of PAA 3-mer (11).

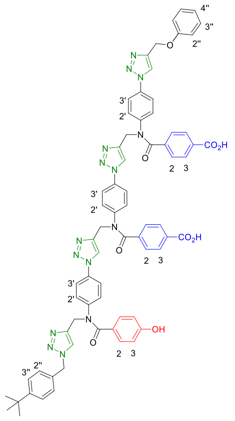

Compound **S7** (0.020 g, 0.02 mmol), 1-(azidomethyl)-4-*tert*-butylbenzene (0.005 g, 0.03 mmol), $Cu(CH_3CN)_4PF_6$ (0.6 mg, 0.002 mmol) and TBTA (1.0 mg, 0.002 mmol) were mixed in a round-bottom flask and, under N_2, THF (3 mL) was added. The reaction was stirred overnight at room temperature. Then, H_2O was added (1.5 mL) followed by LiOH (4.5 mg, 0.08 mmol) and the reaction was stirred at room temperature for 2h. Then, the crude was diluted with H_2O and acidified with 1 M HCl soln. to pH 2-3. The aqueous phase was extracted with EtOAc (3x) and the combined organic phase was washed with EDTA soln. (2x), H_2O (1x) and brine (1x), dried over $MgSO_4$ and evaporated to dryness. The residue was purified by flash chromatography on silica gel (gradient from 0% to 20% of MeOH in CH_2Cl_2) to afford compound **11** (0.016 g, 70%) as a white powder.

^{1}H NMR (400 MHz, DMSO-*d*$_6$): δ_H = 8.85 (s, 1H, CH_{triaz}, PhO cap), 8.75 (s, 1H, CH_{triaz}, acid), 8.70 (s, 1H, CH_{triaz}, phenol), 8.05 (s, 1H, CH_{triaz}, ^{t}Bu cap), 7.76 (m, 10H, 3-H, acid; 3'-H), 7.42 (m, 8H, 2-H, acid; 2'-H), 7.28 (m, 6H, 2-H, acid; 3''-H, PhO cap; 3''-H, ^{t}Bu cap), 7.17 (d, 2H, *J* = 8.5 Hz, 2-H, phenol), 7.08 (d, 2H, *J* = 8.0 Hz, 2''-H, ^{t}Bu cap), 7.03 (d, 2H, *J* = 8.0 Hz, 2''-H, PhO cap), 6.94 (t, 1H, *J* = 7.5 Hz, 4''-H, PhO cap), 6.60 (d, 2H, *J* = 8.5 Hz, 3-H, phenol), 5.48 (s, 2H, N-CH_2, ^{t}Bu cap), 5.21 (s, 2H, N-CH_2, acid), 5.17 (s, 4H, N-CH_2, PhO cap; N-CH_2, acid), 5.11 (s, 2H, N-CH_2, phenol), 1.17 (s, 9H, ^{t}Bu).

^{13}C NMR (100.6 MHz, DMSO-*d*$_6$): δ_C = 169.2 (CO, amide phenol), 168.8 (CO, amide acid), 165.4 (CO, acid), 159.3 (4-C, phenol), 157.9 (1''-C, PhO cap), 150.5 (4''-C, ^{t}Bu cap), 144.3, 144.2 and 143.9 (C_{triaz}), 143.6 and 143.5 (1'-C), 143.4 (C_{triaz}), 142.7 and 142.5 (1'-C), 139.5 and 139.4 (1-C, acid), 134.6, 134.6 and 134.5 (4'-C), 134.0 (4-C, acid), 133.2 (1''-C, ^{t}Bu cap), 130.8 (2-C, phenol), 130.3, 129.5, 128.7, 128.1 and 127.3 (C_{arom}), 125.6 (1-C, phenol), 125.4 (3''-C, ^{t}Bu cap), 124.0 (CH_{triaz}, ^{t}Bu cap), 122.8 (CH_{triaz}, PhO cap), 121.6 and 121.5 (CH_{triaz}), 120.9 (4''-C, PhO cap), 120.6, 120.5 and 120.3 (3'-C), 114.7 (2''-C, PhO cap), 114.6 (3-C, phenol), 60.9 (O-CH_2), 52.4 (N-CH_2, ^{t}Bu cap), 45.2, 45.1 and 45.1 (N-CH_2), 34.2 (C, ^{t}Bu), 31.0 (CH_3, ^{t}Bu).

HRMS (ES+): calcd for $C_{70}H_{60}N_{15}O_9$ 1254.4698 $[M+H]^+$, found 1254.4702 $[M+H]^+$.

FT-IR (ATR): ν_{max} 2959, 2924, 2853, 1708, 1599, 1516, 1278, 1233, 1023, 844, 752 and 735 cm^{-1}.

^{1}H-NMR (400 MHz, DMSO-d_6) compound 11.

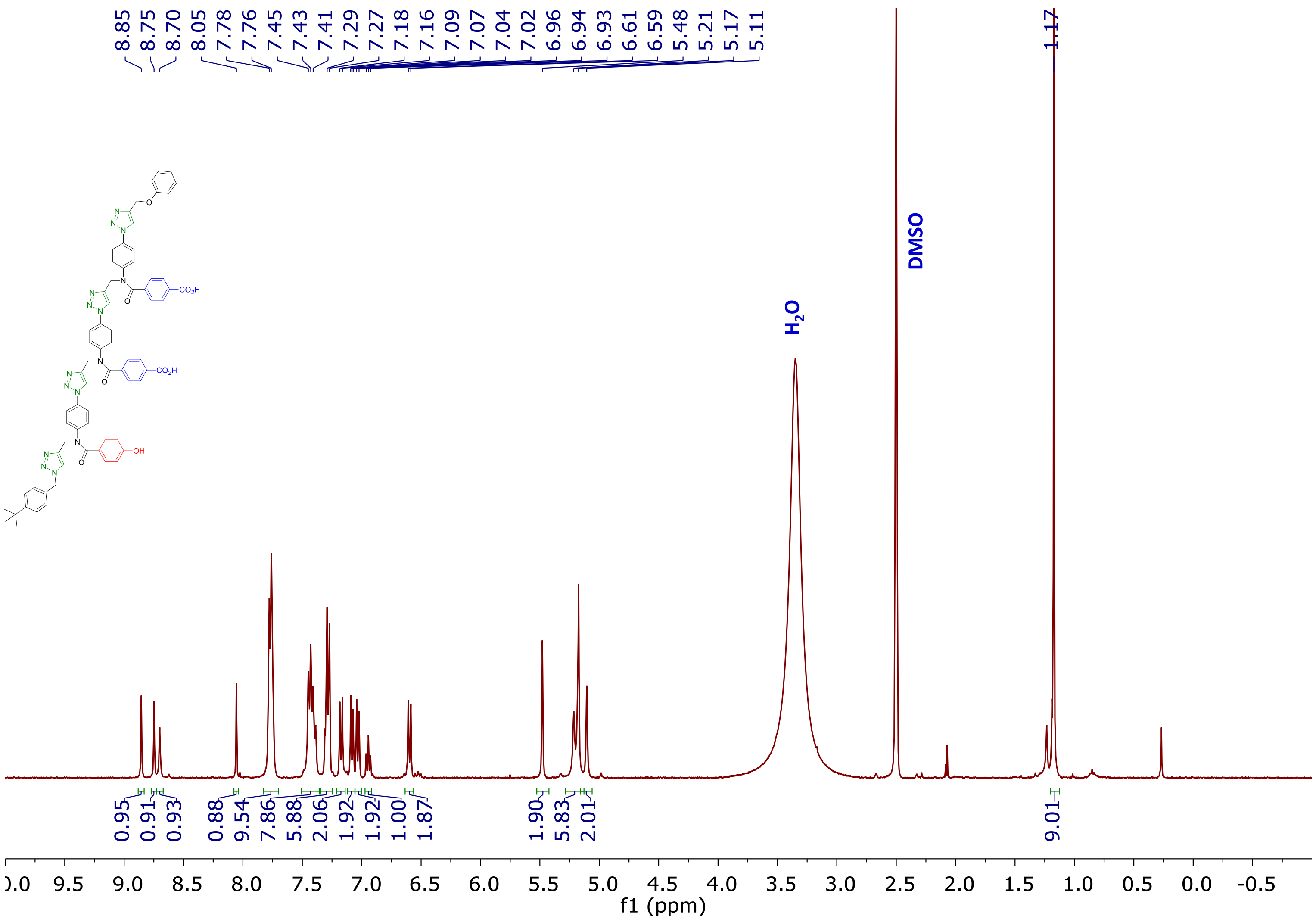

^{13}C-NMR (100.6 MHz, DMSO-d_6) compound 11.

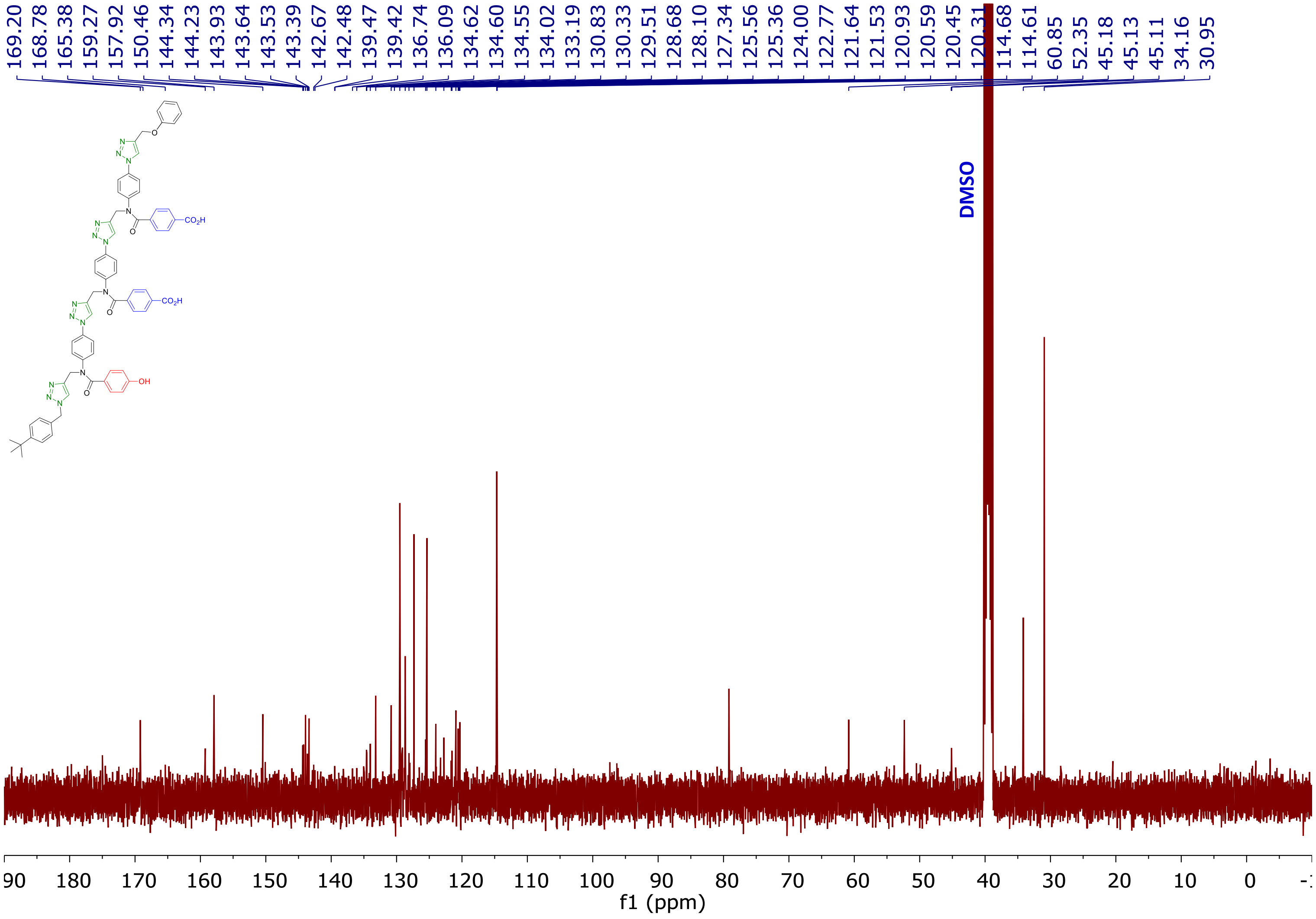

Synthesis of PPP 3-mer (15).

Scheme S4 shows the synthetic route towards PPP 3-mer oligomer **15**. CuAAC reaction between compound **S8**[S1] and **S2**[S1] followed by TBAF-mediate removal of silyl protecting groups afforded the corresponding alkyne-terminated 3-mer. Subsequent capping of this 3-mer with *tert*-butyl benzyl azide gave access to 3-mer **15** in moderate yield.

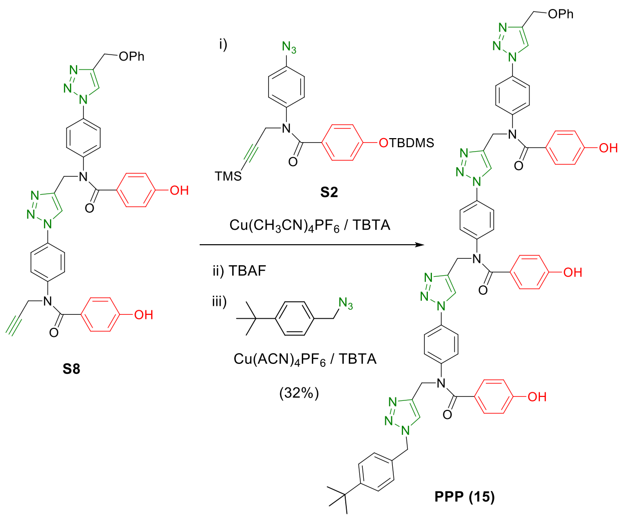

Scheme S4. Synthesis of PPP 3-mer oligomer (**15**).

Synthesis of PPP 3-mer (15).

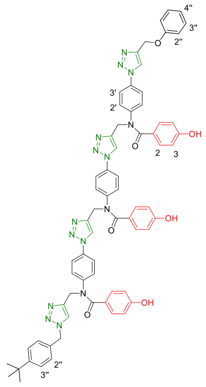

Compound **S8**[S1] (0.008 g, 0.01 mmol), compound **S2**[S1] (0.005 g, 0.01 mmol), $Cu(CH_3CN)_4PF_6$ (0.4 mg, 0.001 mmol) and TBTA (0.6 mg, 0.001 mmol) were mixed in a round-bottom flask and, under N_2, THF (2 mL) was added. The reaction was stirred overnight at room temperature. Once the reaction was completed, TBAF (1 M in THF, 0.02 mL, 0.02 mmol) was added dropwise. The solution was stirred for 10 min at room temperature, quenched with 0.1 M HCl soln. and extracted with EtOAc (3x) followed by washing with H_2O and brine. The organic layer was dried over anhydrous $MgSO_4$ and concentrate under vacuum. The crude was mixed with 1-(azidomethyl)-4-*tert*-butylbenzene (0.006 g, 0.03 mmol), $Cu(CH_3CN)_4PF_6$ (0.4 mg, 0.001 mmol) and TBTA (0.6 mg, 0.001 mmol) in a round-bottom flask and, under N_2, THF (2 mL) was added. The reaction was stirred overnight at room temperature. The reaction was then diluted with EtOAc and washed with EDTA soln. (2x), H_2O (1x) and brine. The organic layer was dried over $MgSO_4$ and concentrate under vacuum. The crude was purified by flash column chromatography on silica gel (gradient from 0% to 10% of MeOH in CH_2Cl_2) to afford compound **15** (0.004 g, 32%) as a white amorphous powder.

^{1}H NMR (400 MHz, DMSO-*d*$_6$): δ_H = 9.88 (bs, 3H, OH), 8.88 (s, 1H, CH_{triaz}, PhO cap), 8.70 (s, 2H, CH_{triaz}), 8.06 (s, 1H, CH_{triaz}, ^{t}Bu cap), 7.78 (m, 6H, 3'-H), 7.41 (d, 2H, *J* = 8.5 Hz, 2'-H), 7.40 (d, 2H, *J* = 8.5 Hz, 2'-H), 7.29 (m, 6H, 2'-H; 3''-H, ^{t}Bu cap; 3''-H, PhO cap), 7.19 (m, 6H, 2-H), 7.08 (d, 2H, *J* = 8.0 Hz, 2''-H, ^{t}Bu cap), 7.05 (d, 2H, *J* = 8.5 Hz, 2''-H, PhO cap), 6.95 (t, 1H, *J* = 7.5 Hz, 4''-H, PhO cap), 6.59 (d, 6H, *J* = 8.5 Hz, 3-H), 5.48 (s, 2H, N-CH_2, ^{t}Bu cap), 5.19 (s, 2H, N-CH_2, PhO cap), 5.17 (s, 4H, N-CH_2), 5. 11 (s, 2H, N-CH_2), 1.17 (s, 9H, ^{t}Bu).

^{13}C NMR (100.6 MHz, DMSO-*d*$_6$): δ_C = 169.3, 169.2 and 169.2 (CO, amide), 159.1, 159.1 and 159.1 (4-C), 158.0 (1''-C, PhO cap), 150.5 (4''-C, ^{t}Bu cap), 144.7 and 144.6 (C_{triaz}), 144.0 (C_{triaz}, PhO cap), 143.9, 143.7 and 143.6 (1'-C), 143.4 (C_{triaz}, ^{t}Bu cap), 134.2, 134.2 and 134.1 (4'-C), 133.2 (1''-C, ^{t}Bu cap), 131.0 and 130.9 (2-C), 129.6, 128.9, 128.8, 128.7 and 127.4 (C_{arom}), 125.7, 125.7 and 125.7 (1-C, phenol), 125.4 (3''-C, ^{t}Bu cap), 124.0 (CH_{triaz}, ^{t}Bu cap), 122.8 (CH_{triaz}, PhO cap), 121.6 and 121.5 (CH_{triaz}), 121.0 (4''-C, PhO cap), 120.6, 120.4 and 120.3 (3'-C), 114.7 (2''-C, PhO cap), 114.6 and 114.5 (3-C, phenol), 60.9 (O-CH_2), 52.4 (N-CH_2, ^{t}Bu cap), 45.5 and 45.1 (N-CH_2), 34.2 (C, ^{t}Bu), 31.0 (CH_3, ^{t}Bu).

HRMS (ES+): calcd for $C_{68}H_{60}N_{15}O_7$ 1198.4800 $[M+H]^+$, found 1198.4795 $[M+H]^+$.

FT-IR (ATR): ν_{max} 3637, 2917, 2850, 1631, 1518, 1280, 1233 and 843 cm^{-1}.

^{1}H-NMR (400 MHz, DMSO-d_6) compound 15.

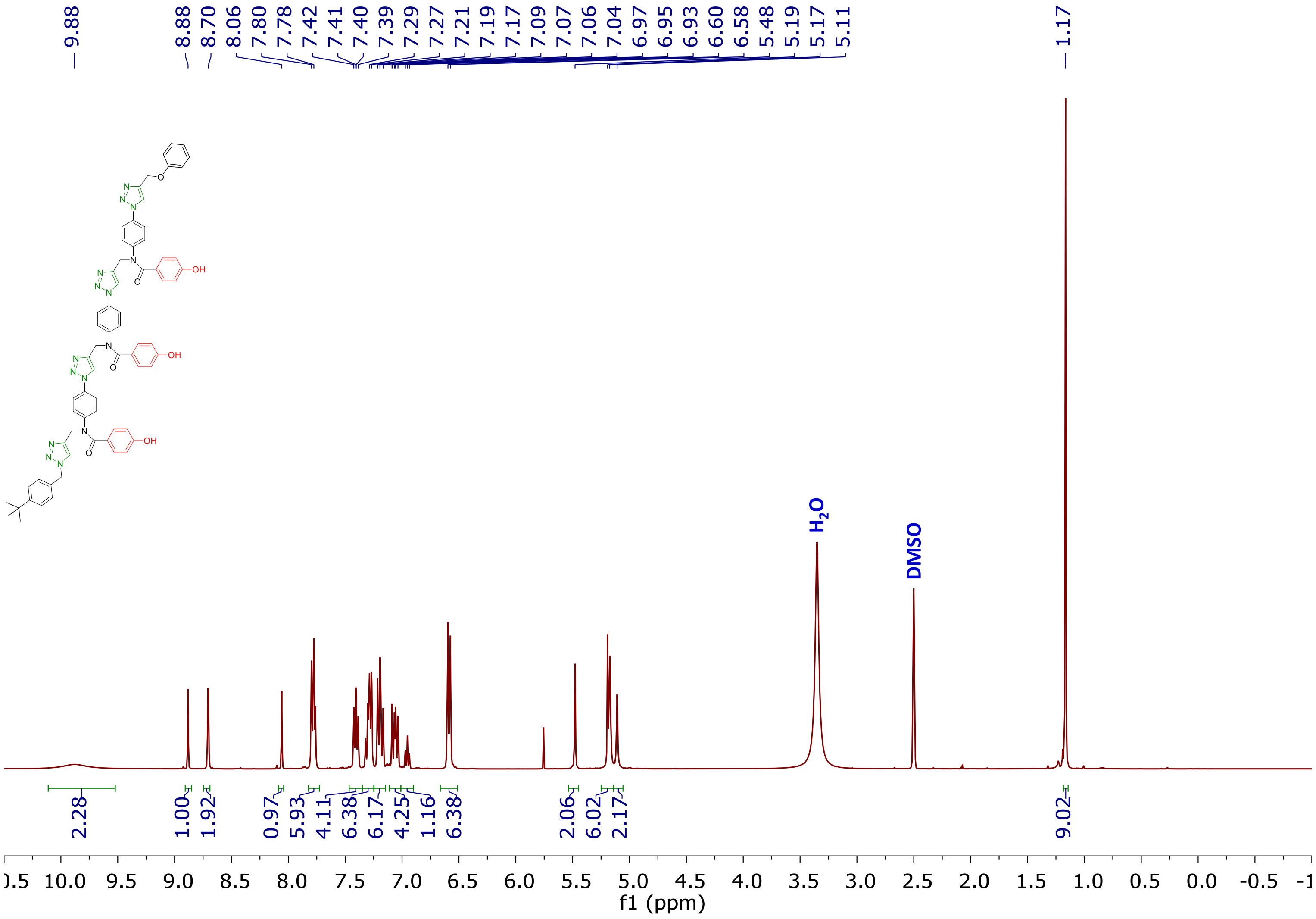

^{13}C-NMR (100.6 MHz, DMSO-d_6) compound 15.

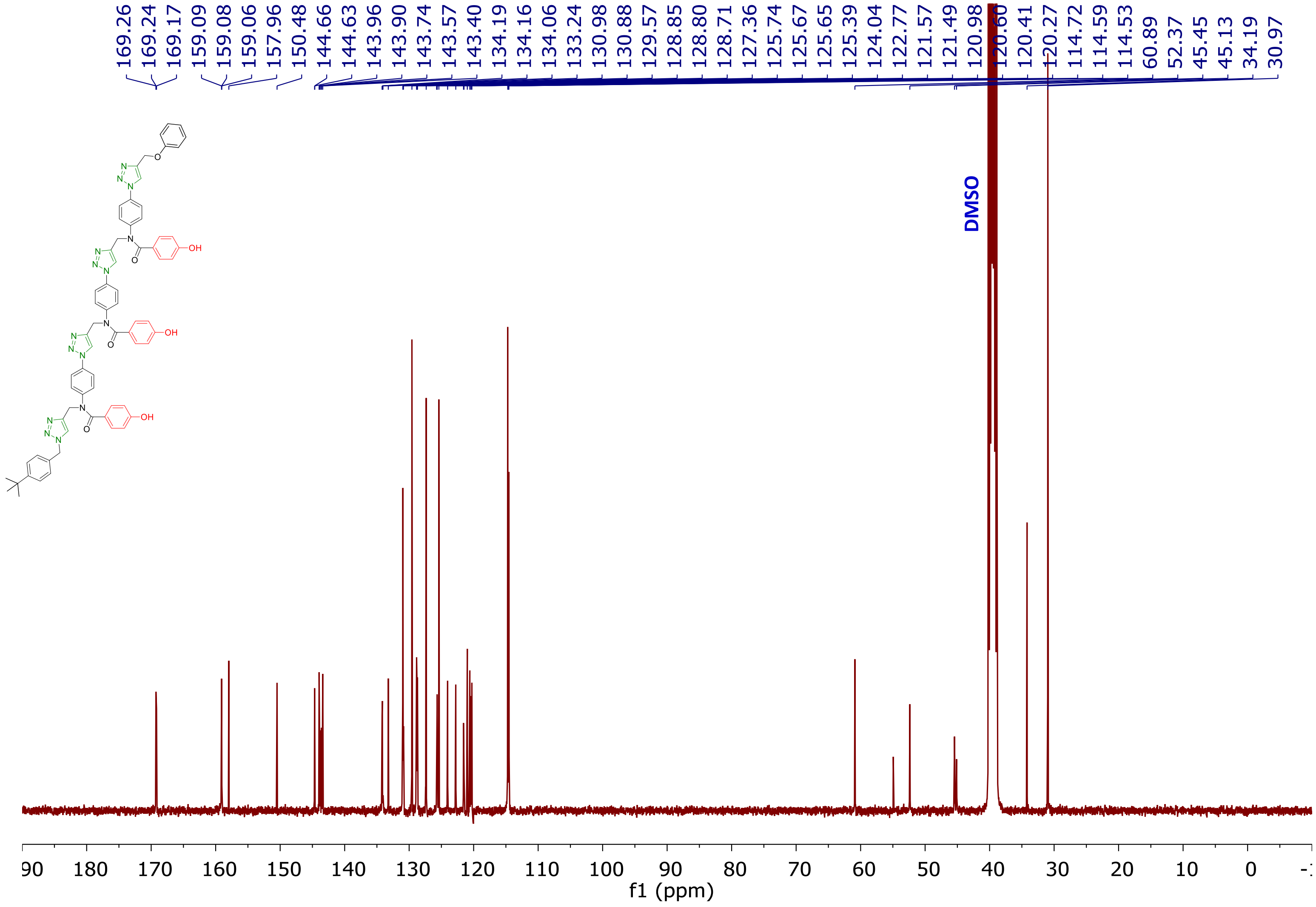

Covalent template-directed mutation of chemical information encoded in template 1.

Scheme S5 summarizes the process for the covalent template-directed mutation of chemical information encoded in template **1**. This process encompasses four steps:

1) *Monomer attachment*: Template **1** was loaded with different proportions of 1-mers **4** and **7** via ester coupling using EDC/DMAP as coupling reagents. This step determines the mutation rate of the process, as the obtained preZIP intermediates are enriched in phenol (X=O; Y=CO) or benzoic acid (X=CO; Y=O) 1-mers according to the initial ratio of 1-mers **4** and **7** used.

2) *ZIP*: CuAAC intramolecular reaction between the reactive groups of the attached 1-mers leads to the formation of the corresponding duplexes. The reaction is carried out in the presence of an excess of a capping azide (*tert*-butylbenzyl azide), which reacts with the pendant alkyne group of the duplexes preventing cyclization and intermolecular oligomerization through the reactive chain ends after intramolecular reaction.[S5,S6] As the backbone is directional, there are two possible arrangements of the duplexes: parallel and antiparallel in regard to the backbone direction (the duplexes shown in Scheme S5 shows the antiparallel arrangement).

3) *Capping*: Phenyl propargyl ether was used to cap the pendant azide groups in the obtained duplexes

4) *Cleavage*: Basic hydrolysis of the ester groups promote the release of the biphenyl traceless linkers. The template 1 is regenerated along with the 3-mer oligomer sequences **8**-**15**. The proportion of this oligomer sequences depends on the mutation rate determined in the attach step.

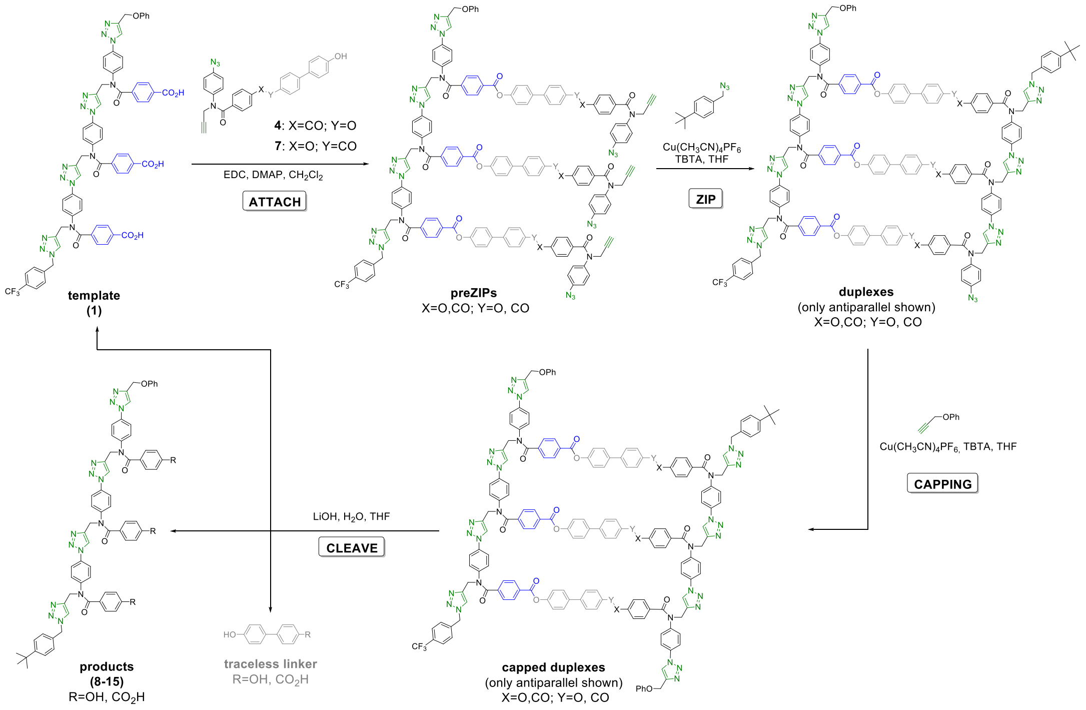

Scheme S5.

Step 1: Monomer attachment.

Scheme S6 shows the synthetic approach for the attachment of 1-mers to the template. Five different experiments were performed using a different ratio of 1-mers **4** and **7**.

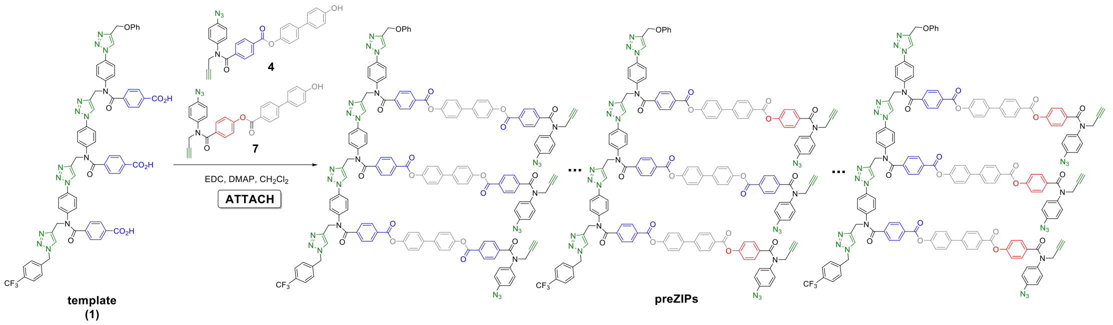

Scheme S6.

➢ **General procedure for monomer attachment.**

Template **1**, 1-mers **4** and **7**, EDC and DMAP were mixed in a round-bottom flask and, under N_2, CH_2Cl_2 was added. The reaction was stirred overnight at room temperature. The solution was diluted with EtOAc and washed with 0.1 M HCl soln. (3x), H_2O (1x) and brine. The organic phase was dried with $MgSO_4$ and concentrated. The crude material was purified by flash column chromatography on silica gel (gradient from 0% to 60% of EtOAc in Pet. Ether and then gradient from 0% to 4% of MeOH in CH_2Cl_2) to give the corresponding pre-ZIP intermediates.

➢ **Experiment 1.**

Reagents and solvent: Template **1** (0.014 g, 0.011 mmol), 1-mer **4** (0.018 g, 0.037 mmol), EDC (0.011 g, 0.056 mmol), DMAP (0.007 g, 0.056 mmol) and CH_2Cl_2 (2 mL).

PreZIP intermediate: pre-ZIP **S9** (0.022 g, 72%) as a white foam. Fig. S2 shows the UPLC traces of the starting template, reaction crude and pure pre-ZIP **S9**. Full characterization of pre-ZIP **S9** appears in the next page.

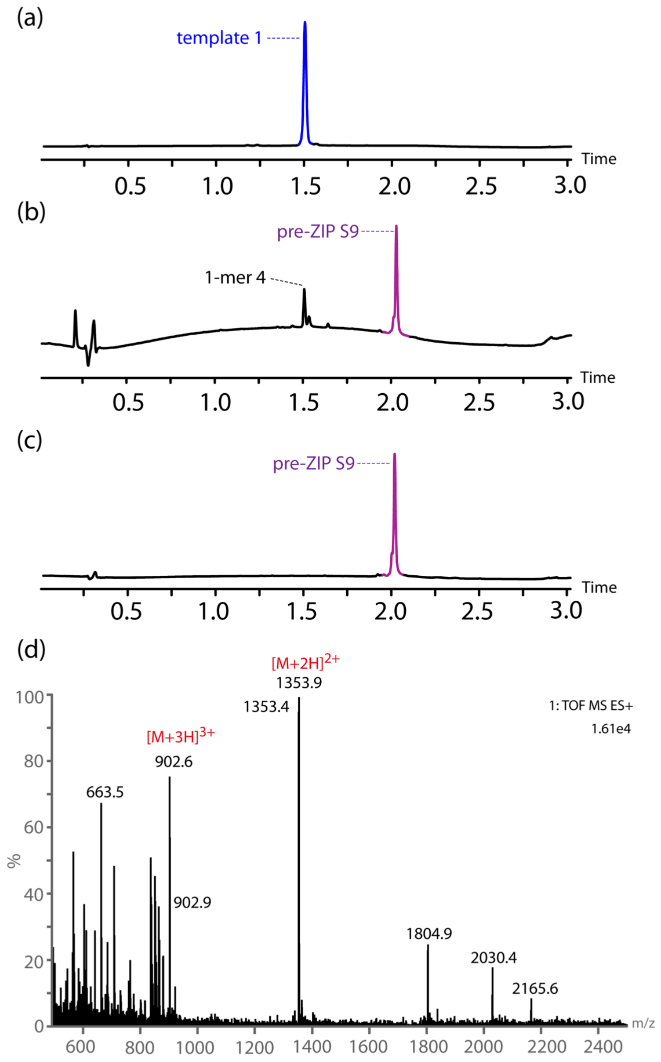

Fig S2. UPLC traces of: (a) starting template **1**. (b) Reaction crude for the attach step. (c) Pure pre-ZIP **S9**. (d) MS spectrum of pure pre-ZIP **S9** (MW: 2703.8). *UPLC Conditions*: C18 column at 40 °C (254 nm) using water + 0.1% formic acid (A) and CH_3CN + 0.1% formic acid (B) with flow rate of 0.6 ml/min; Gradient of 0-2 minutes 5% -100% B + 1 minute 100% B.

*Full characterization of Pre-ZIP **S9**.*

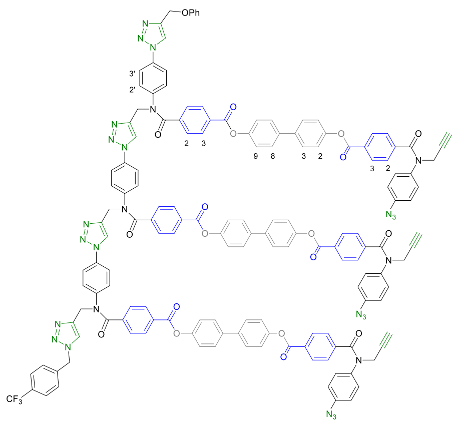

^{1}H NMR (400 MHz, CDCl$_3$): δ_H = 8.22 (s, 2H, CH$_{triaz}$), 8.04 (m, 12H, 3-H and 3-H, 1-mer), 8.00 (s, 1H, CH$_{triaz}$, PhO cap), 7.75 (s, 1H, CH$_{triaz}$, CF$_3$ cap), 7.66 (m, 8H, 3'-H and 3-H, CF$_3$ cap), 7.58 (m, 12H, 3-H and 8-H, biph), 7.45 (m, 12H, 2-H and 2-H, 1-mer), 7.38 (m, 8H, 2'-H and 2-H, CF$_3$ cap), 7.29 (m, 2H, 3-H, PhO cap), 7.23 (m, 12H, 2-H and 9-H, biph), 7.14 (m, 6H, 2'-H, 1-mer), 6.98 (m, 3H, 2-H and 4-H, PhO cap), 6.93 (m, 6H, 3'-H, 1-mer), 5.60 (s, 2H, N-CH$_2$, CF$_3$ cap), 5.27 (s, 2H, O-CH$_2$), 5.24 (s, 2H, N-CH$_2$, internal), 5.23 (s, 2H, N-CH$_2$, internal), 5.17 (s, 2H, N-CH$_2$, internal, next to CF$_3$ cap), 4.68 (m, 6H, N-CH$_2$, 1-mer), 2.29 (m, 3H, CH, alkyne).

^{13}C NMR (100.6 MHz, CDCl$_3$): δ_C = 169.4, 169.3 and 169.3 (CO, amide), 164.5, 164.4 and 164.3 (CO, ester), 158.2 (1-C, PhO cap), 150.4 and 150.3 (1-C and 10-C, biph), 145.5 (C$_{triaz}$, PhO cap), 144.4, 144.4 and 144.2 (C$_{triaz}$), 143.7 (1'-C), 140.1, 139.9 and 139.7 (1-C), 139.4 (1'-C, 1-mer), 138.9 (4'-C, 1-mer), 138.4, 138.4, 138.4 and 138.3 (1-C, CF$_3$ cap; 4-C and 7-C, biph), 135.6, 135.5 and 135.5 (4'-C), 131.1, 131.0 and 130.7 (4-C; 4-C, 1-mer; 4-C, CF$_3$ cap), 130.1 (3-C and 3-C, 1-mer), 129.9, 129.8, 129.4, 129.0, 128.9, 128.9, 128.9, 128.4 and 128.4 (C$_{arom}$), 126.3 (q, J = 4.0 Hz, 3-C, CF$_3$ cap), 122.2, 122.2 and 122.0 (9-C, biph; CH$_{triaz}$), 121.6, 121.5, 121.4 and 121.3 (3-C, 1-mer; 3'-C), 120.8 (CH$_{triaz}$, PhO cap), 120.1 (3'-C, 1-mer), 114.9 (2-C, PhO cap), 78.6 (C, alkyne), 73.0 (CH, alkyne), 62.0 (O-CH$_2$), 53.7 (N-CH$_2$, CF$_3$ cap), 46.4, 46.4 and 46.4 (N-CH$_2$), 39.9 and 39.8 (N-CH$_2$, 1-mer).

^{19}F NMR (376 MHz, CDCl$_3$): δ_F = -63.7.

HRMS (ES+): calcd for $C_{155}H_{105}F_3N_{27}O_{19}$ 1353.4016 $[M+2H]^{2+}$, found 1353.4032 $[M+2H]^{2+}$.

FT-IR (ATR): ν_{max} 2921, 2851, 2125, 2096, 1736, 1649, 1505, 1263, 1199, 1018 and 729 cm^{-1}.

^{1}H-NMR (400 MHz, $CDCl_3$) pre-ZIP S9.

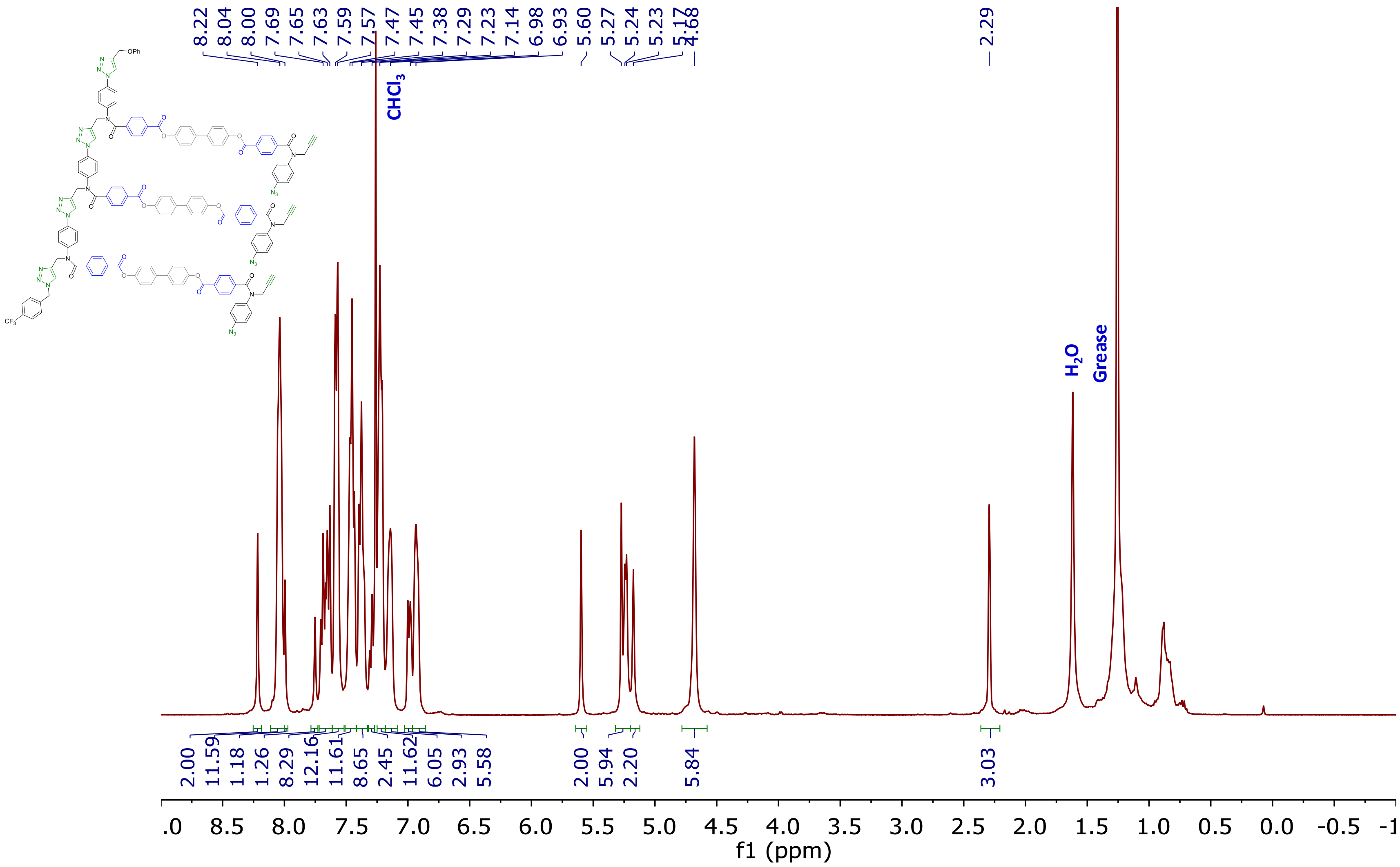

^{13}C-NMR (100.6 MHz, $CDCl_3$) pre-ZIP S9.

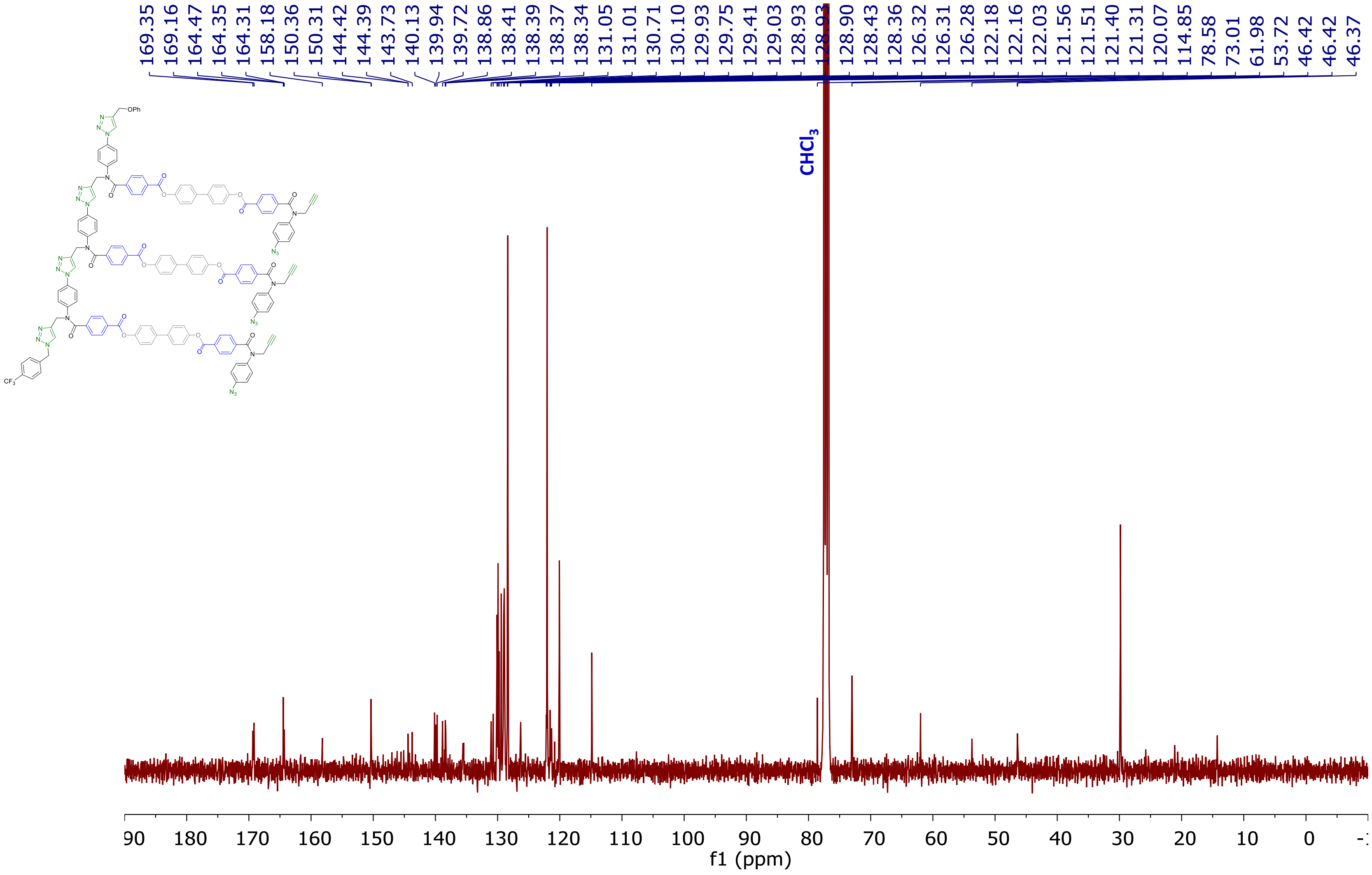

^{19}F NMR (376 MHz, $CDCl_3$) pre-ZIP S9.

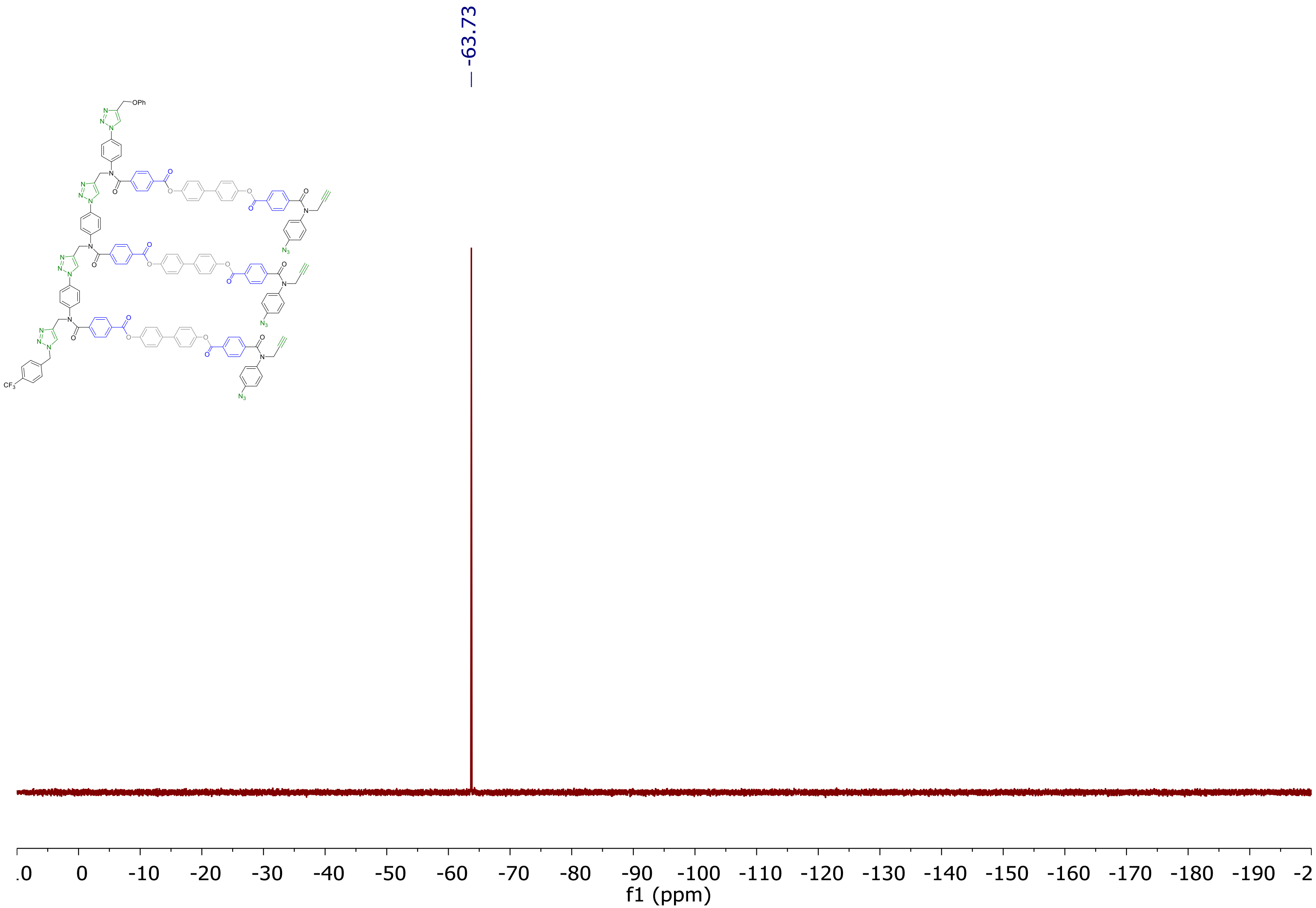

➢ **Experiment 2.**

Reagents and solvent: Template **1** (0.007 g, 0.006 mmol), 1-mer **4** (0.007 g, 0.014 mmol), 1-mer **7** (0.003 g, 0.006 mmol), EDC (0.006 g, 0.029 mmol), DMAP (0.004 g, 0.029 mmol) and CH_2Cl_2 (2 mL).

PreZIP intermediates: 0.012 g as a white foam (78%). Fig. S3 shows the UPLC traces of the starting template, reaction crude and pure pre-ZIP intermediates. The ratio of phenol and benzoic acid monomers attached to the template, which directly relates to the mutation rate, was determined using ^{1}H-NMR, as shown in the next section for all the experiments.

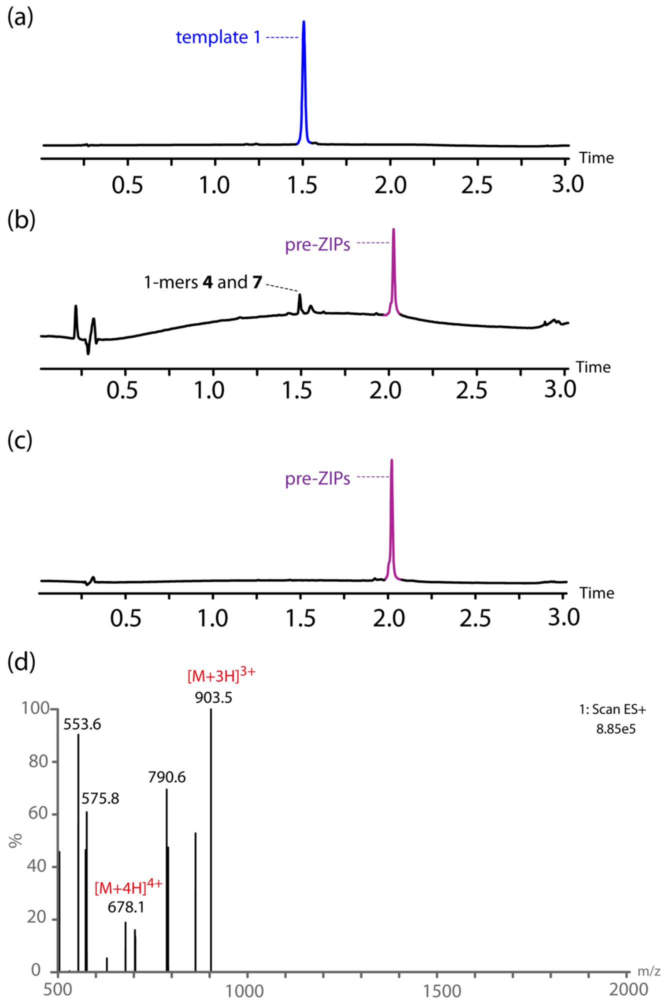

Fig S3. UPLC traces of: (a) starting template **1**. (b) Reaction crude for the attach step. (c) Pure pre-ZIP intermediates from experiment 2. (d) MS spectrum of pure pre-ZIP intermediates (MW: 2703.8). *UPLC Conditions*: C18 column at 40 °C (254 nm) using water + 0.1% formic acid (A) and CH_3CN + 0.1% formic acid (B) with flow rate of 0.6 ml/min; Gradient of 0-2 minutes 5% -100% B + 1 minute 100% B.

- **Experiment 3.**

Reagents and solvent: Template **1** (0.009 g, 0.007 mmol), 1-mer **4** (0.006 g, 0.011 mmol), 1-mer **7** (0.006 g, 0.011 mmol), EDC (0.007 g, 0.035 mmol), DMAP (0.004 g, 0.035 mmol) and CH_2Cl_2 (3 mL).

PreZIP intermediates: 0.009 g as a white foam (49%). Fig. S4 shows the UPLC traces of the starting template, reaction crude and pure pre-ZIP intermediates. The ratio of phenol and benzoic acid monomers attached to the template, which directly relates to the mutation rate, was determined using ^{1}H-NMR, as shown in the next section for all the experiments.

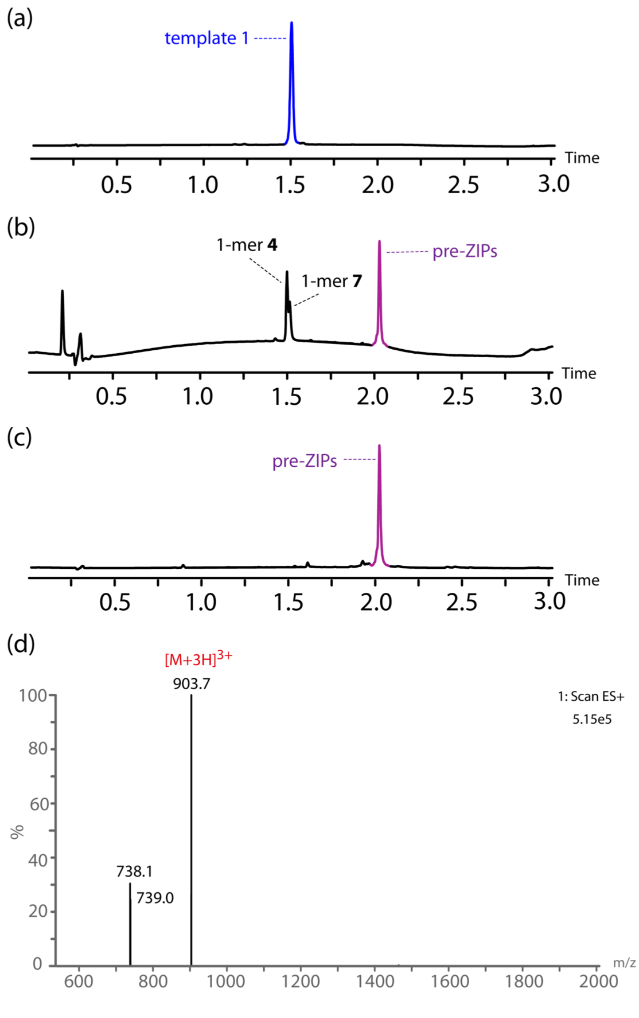

Fig S4. UPLC traces of: (a) starting template **1**. (b) Reaction crude for the attach step. (c) Pure pre-ZIP intermediates from experiment 3. (d) MS spectrum of pure pre-ZIP intermediates (MW: 2703.8). *UPLC Conditions*: C18 column at 40 °C (254 nm) using water + 0.1% formic acid (A) and CH_3CN + 0.1% formic acid (B) with flow rate of 0.6 ml/min; Gradient of 0-2 minutes 5% -100% B + 1 minute 100% B.

➢ **Experiment 4.**

Reagents and solvent: Template **1** (0.008 g, 0.006 mmol), 1-mer **4** (0.003 g, 0.007 mmol), 1-mer **7** (0.008 g, 0.016 mmol), EDC (0.007 g, 0.034 mmol), DMAP (0.004 g, 0.034 mmol) and CH_2Cl_2 (2.5 mL).

PreZIP intermediates: 0.022 g as a white foam (72%). Fig. S5 shows the UPLC traces of the starting template, reaction crude and pure pre-ZIP intermediates. The ratio of phenol and benzoic acid monomers attached to the template, which directly relates to the mutation rate, was determined using ^{1}H-NMR, as shown in the next section for all the experiments.

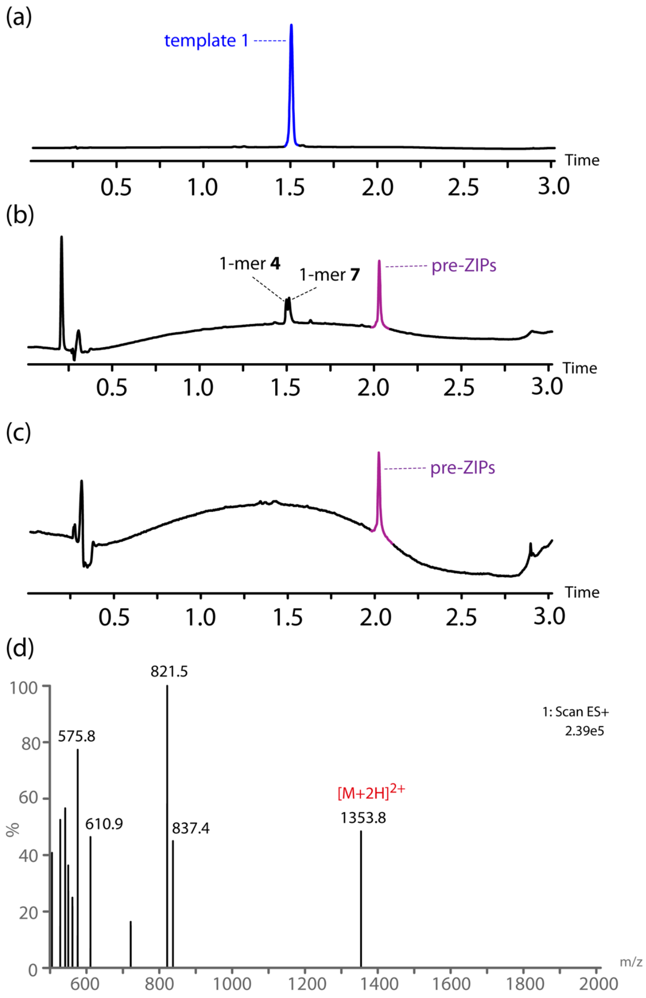

Fig S5. UPLC traces of: (a) starting template **1**. (b) Reaction crude for the attach step. (c) Pure pre-ZIP intermediates from experiment 4. (d) MS spectrum of pure pre-ZIP intermediates (MW: 2703.8). *UPLC Conditions*: C18 column at 40 °C (254 nm) using water + 0.1% formic acid (A) and CH_3CN + 0.1% formic acid (B) with flow rate of 0.6 ml/min; Gradient of 0-2 minutes 5% -100% B + 1 minute 100% B.

- **Experiment 5.**

Reagents and solvent: Template **1** (0.008 g, 0.006 mmol), 1-mer **7** (0.010 g, 0.020 mmol), EDC (0.006 g, 0.031 mmol), DMAP (0.004 g, 0.031 mmol) and CH_2Cl_2 (2 mL).

PreZIP intermediate: pre-ZIP **S10** (0.015 g, 89%) as a white foam. Fig. S6 shows the UPLC traces of the starting template, reaction crude and pure pre-ZIP **S10**. Full characterization of pre-ZIP **S10** is provided.

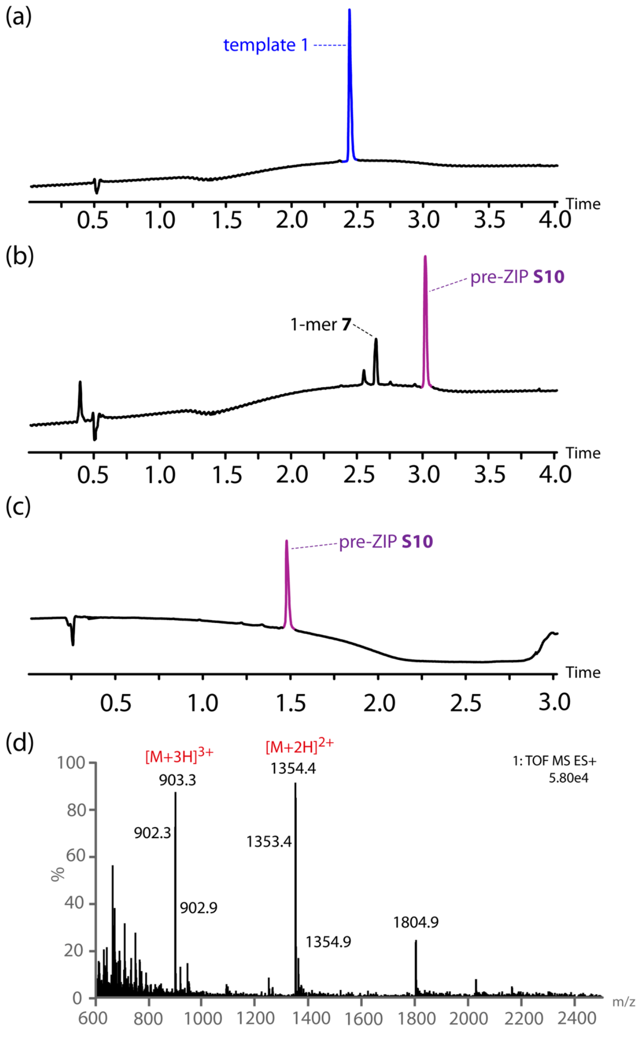

Fig S6. UPLC traces of: (a) starting template **1**. (b) Reaction crude for the attach step. (c) Pure pre-ZIP intermediate **S10**. (d) MS spectrum of pure pre-ZIP **S10** (MW: 2703.8). *UPLC Conditions*: C18 column at 40 ^{o}C (254 nm) using water + 0.1% formic acid (A) and CH_3CN + 0.1% formic acid (B) with flow rate of 0.6 ml/min; Gradient of 0-3 minutes 5% -100% B + 1 minute 100% B for (a) and (b); Gradient of 0-2 minutes 65% -100% B + 1 minute 100% B for (c).

*Full characterization of Pre-ZIP **S10***:

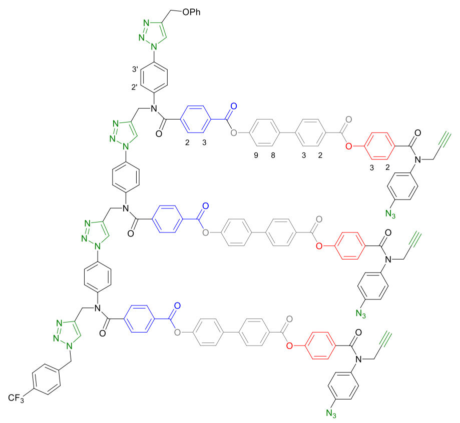

^{1}H NMR (400 MHz, $CDCl_3$): δ_H = 8.22 (s, 2H, CH_{triaz}), 8.19 (m, 6H, 2-H, biph), 8.04 (m, 6H, 3-H), 8.00 (s, 1H, CH_{triaz}, PhO cap), 7.75 (s, 1H, CH_{triaz}, CF_3 cap), 7.65 (m, 20H, 3-H and 8-H, biph; 3'-H; 3-H, CF_3 cap), 7.45 (m, 12H, 2-H and 2-H, 1-mer), 7.38 (m, 8H, 2'-H and 2-H, CF_3 cap), 7.26 (m partially overlapped, 8H, 3-H, PhO cap; 9-H, biph), 7.15 (m, 6H, 2'-H, 1-mer), 7.09 (m, 6H, 3-H, 1-mer), 6.98 (m, 3H, 2-H and 4-H, PhO cap), 6.95 (m, 6H, 3'-H, 1-mer), 5.60 (s, 2H, N-CH_2, CF_3 cap), 5.27 (s, 2H, O-CH_2), 5.24 (s, 2H, N-CH_2, internal), 5.23 (s, 2H, N-CH_2, internal), 5.18 (s, 2H, N-CH_2, internal, next to CF_3 cap), 4.66 (m, 6H, N-CH_2, 1-mer), 2.27 (m, 3H, CH, alkyne).

^{13}C NMR (125 MHz, $CDCl_3$): δ_C = 169.4, 169.3, 169.3 and 169.2 (CO, amide), 164.5, 164.5, 164.3, 164.3 and 164.2 (CO, ester), 158.2 (1-C, PhO cap), 152.3 and 152.3 (4-C, 1-mer), 151.0 and 151.0 (10-C, biph), 145.6 and 145.6 (C_{triaz}, PhO cap; 4-C, biph) 144.4, 144.4 and 144.2 (C_{triaz}), 143.8 and 143.8 (1'-C), 140.2, 140.0 and 140.0 (1-C), 139.5 and 139.4 (1'-C, 1-mer), 138.5 (1-C, CF_3 cap; 4'-C, 1-mer), 137.8 (7-C, biph), 135.6, 135.5 and 135.5 (4'-C), 132.5 (1-C, 1-mer), 131.3 (q, J = 32.5 Hz, 4-C, CF_3 cap), 130.9 and 130.9 (2-C, biph; 4-C), 130.6 (2-C, 1-mer), 130.1 (3-C), 129.8, 129.3, 129.1, 129.1, 128.9, 128.9, 128.6, 128.4 and 128.1 (C_{arom}), 127.4 (3-C, biph), 126.3 (q, J = 4.0 Hz, 3-C, CF_3 cap), 124.2 (CH_{triaz}, CF_3 cap), 122.3, 122.3 and 122.2 (9-C, biph; CH_{triaz}), 121.6, 121.5, 121.4, 121.4 and 121.3 (3-C, 1-mer; 3'-C), 120.8 (CH_{triaz}, PhO cap), 120.1 (3'-C, 1-mer), 114.8 (2-C, PhO cap), 78.9 (C, alkyne), 72.8 (CH, alkyne), 62.0 (O-CH_2), 53.7 (N-CH_2, CF_3 cap), 46.4 and 46.4 (N-CH_2), 40.2 (N-CH_2, 1-mer).

^{19}F NMR (376 MHz, $CDCl_3$): δ_F = -63.7.

HRMS (ES+): calcd for $C_{155}H_{105}F_3N_{27}O_{19}$ 1353.4016 $[M+2H]^{2+}$, found 1353.3925 $[M+2H]^{2+}$.

FT-IR (ATR): ν_{max} 2920, 2851, 2125, 2094, 1736, 1648, 1505, 1261, 1206, 1167 and 1069 cm^{-1}.

^{1}H-NMR (400 MHz, $CDCl_3$) Pre-ZIP S10.

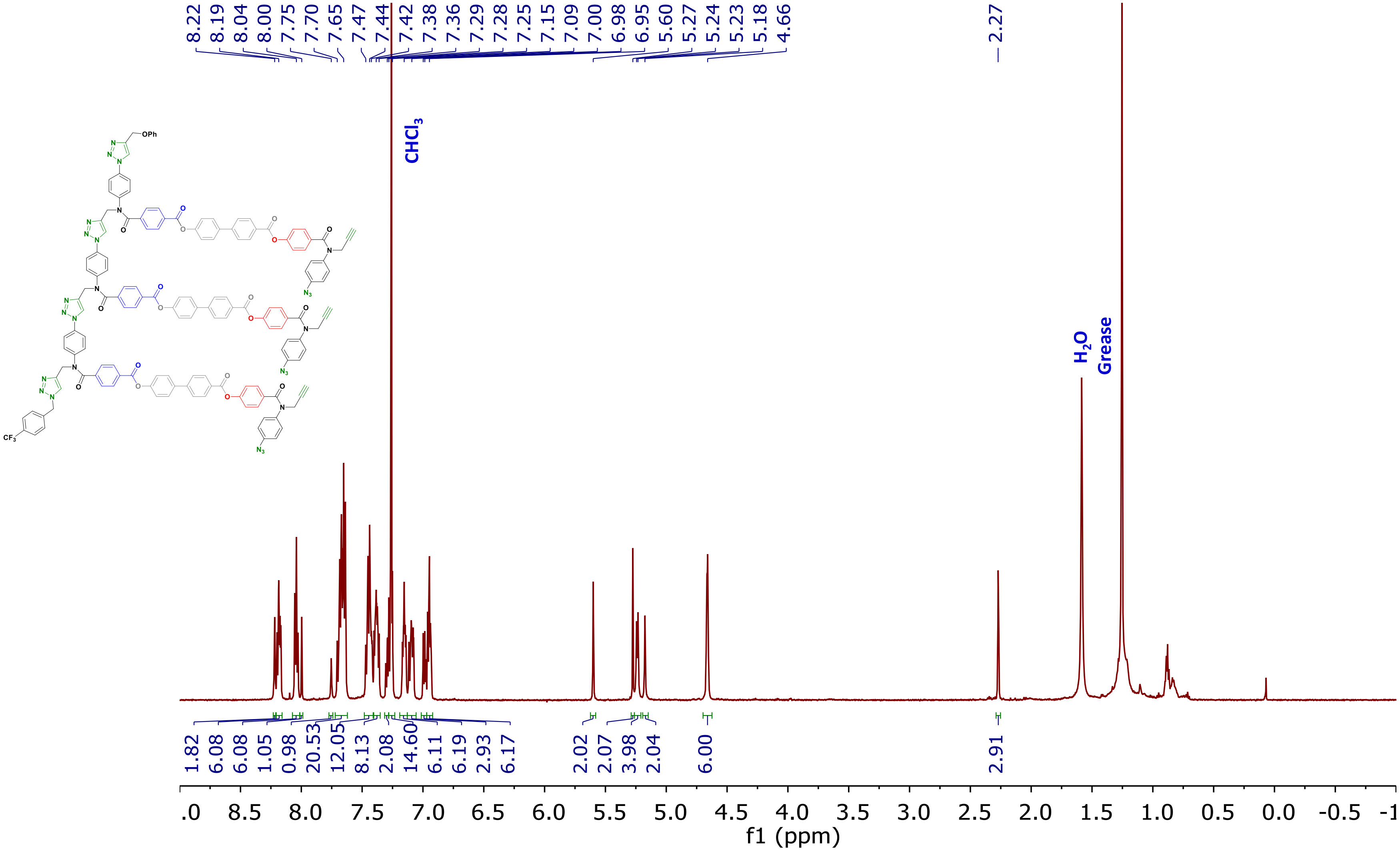

^{13}C-NMR (125 MHz, $CDCl_3$) Pre-ZIP S10.

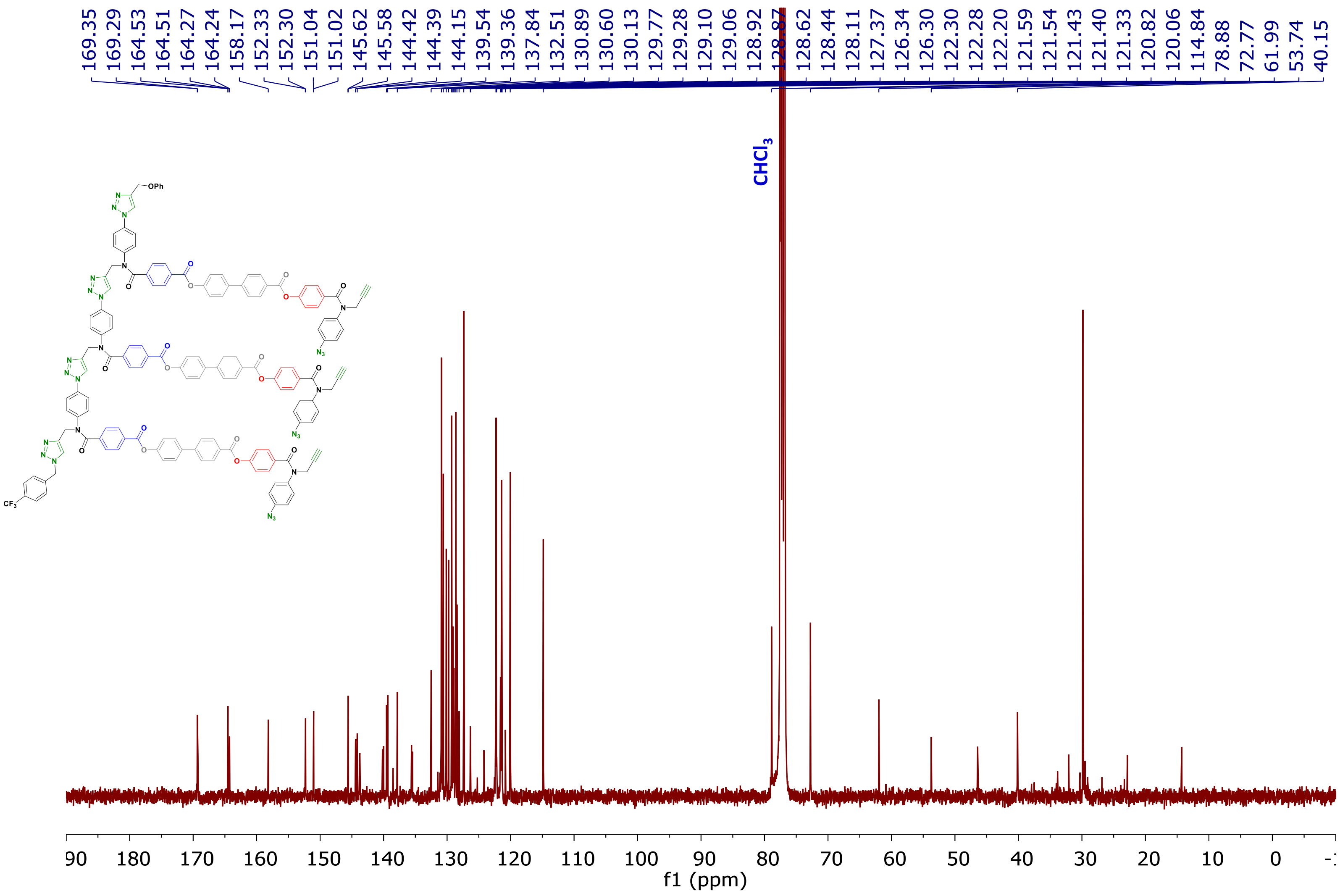

^{19}F NMR (376 MHz, $CDCl_3$) Pre-ZIP S10.

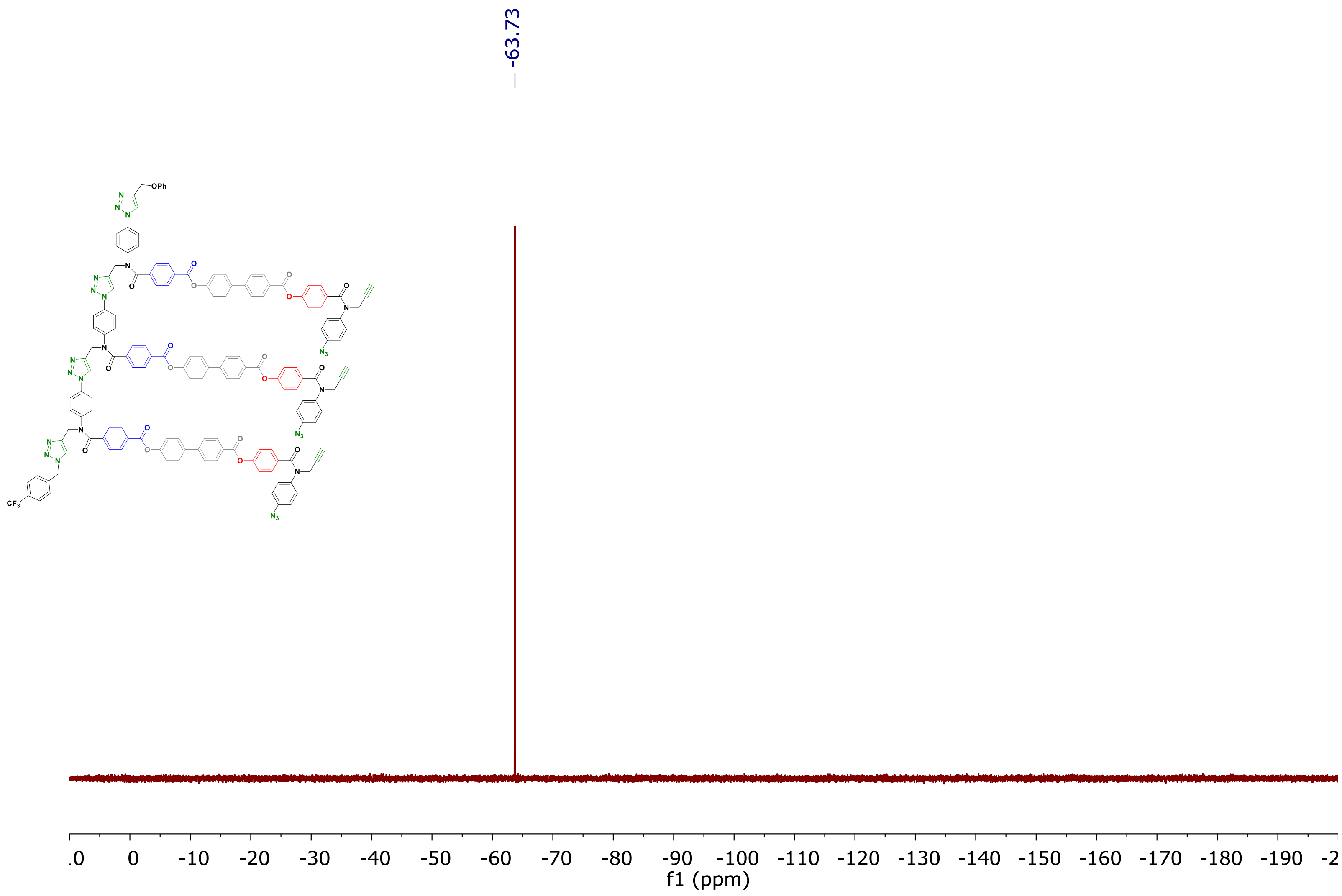

Determination of mutator population in preZIP intermediates

^{1}H NMR was used to quantify the population of mutator in the preZIP intermediates from the 1-mer attach experiments 1-5. Fig. S7 shows the stacked ^{1}H NMR for these preZIP intermediates, with expansions of the regions corresponding to the alkyne CH, methylene groups and the aromatic protons. Some signals can be clearly assigned to the benzoic acid (blue) and phenol (red) 1-mer residues attached to the template.

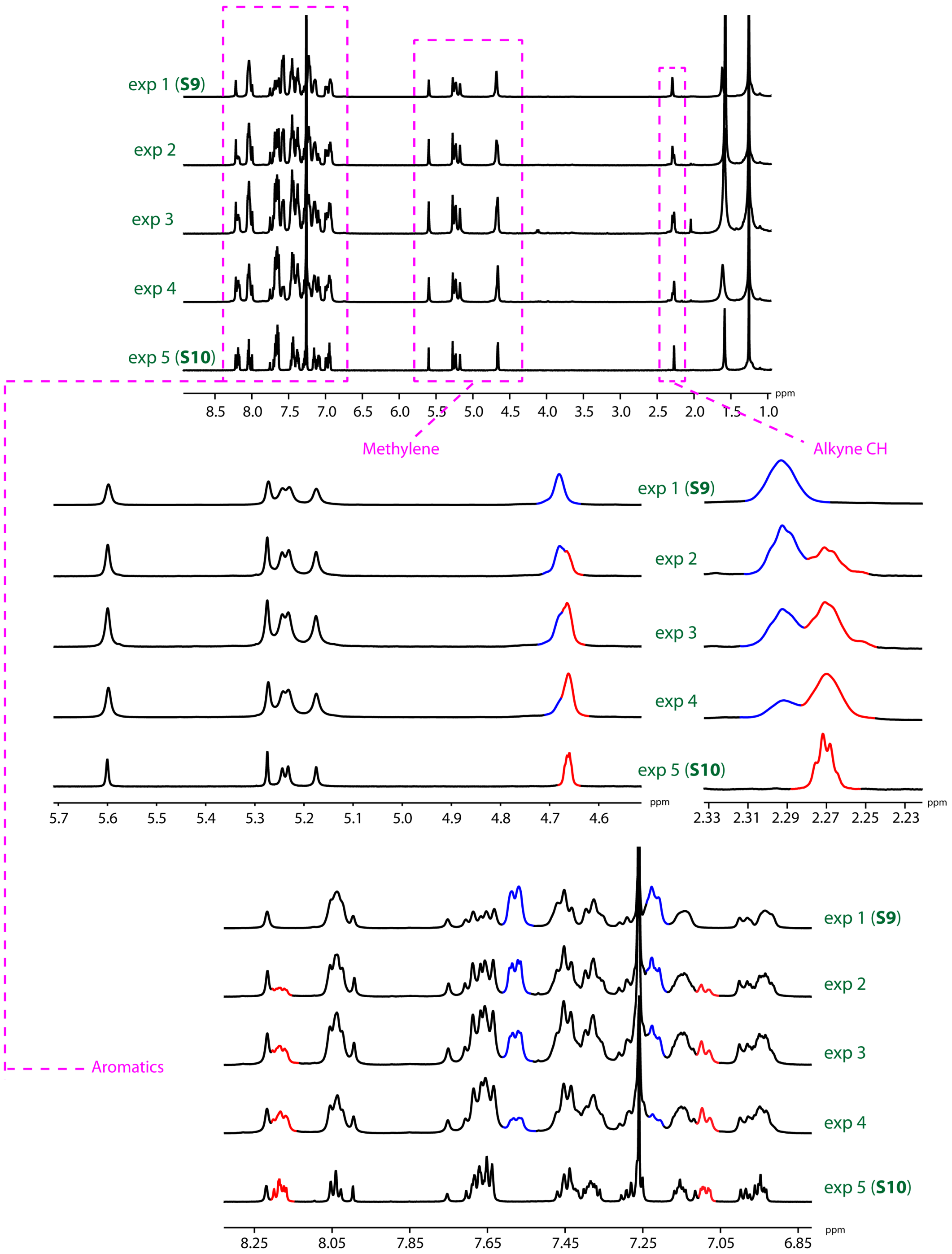

Fig. S7. Stacked ^{1}H NMR for the preZIP intermediates obtained in the monomer attach experiments 1-5 (400 MHz, $CDCl_3$, 298 K). Expansions of the regions corresponding to the alkyne CH, methylene groups and the aromatic protons are shown. Signals assigned to the benzoic acid 1-mer residues are shown in and those for the phenol 1-mers in red.

The alkyne protons for the preZIP intermediates from experiments 2-4 were used to quantify the population of mutator (phenol 1-mer). Deconvolution of these peaks were performed using the Global Spectral Deconvolution (GSD) algorithm provided by MestreNova 10. Fig. S8 shows the fitting model, individual functions and residuals obtained in experiments 2-4. Table S1 shows the areas of the individual curves and the population of mutator extracted from the fitted NMR curves, which are the values used in the *x* axis in Fig. 8 in the text.

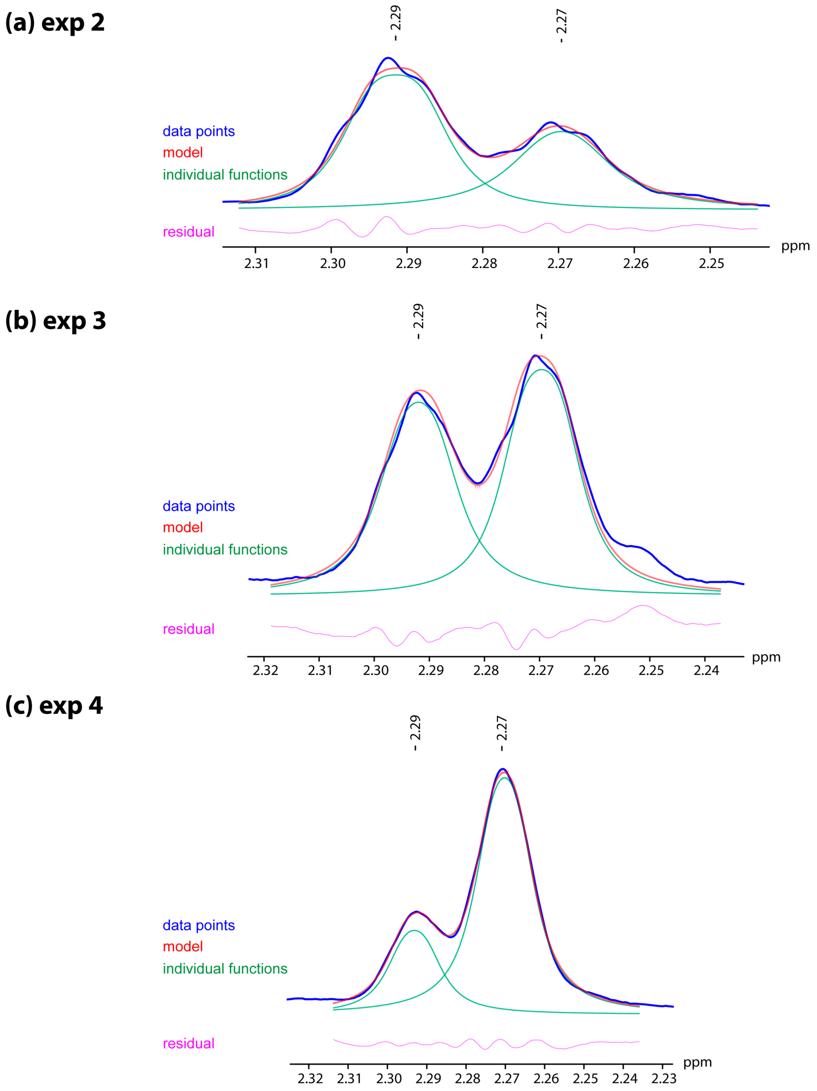

Fig. S8. Deconvolution of the alkyne CH peaks of preZIP intermediates from experiments 2-4 (a-c) using the Global Spectral Deconvolution (GSD) algorithm provided by MestreNova 10. Experimental signal is shown in blue, fitting model in red, individual functions in green and residuals in pink.

Table S1. Areas of the fitted curves shown in green in Fig. S8. The mutator population corresponds to $\chi_{mutator} = A_{2.27ppm} / (A_{2.29ppm} + A_{2.27\ ppm})$.

Attach experiment no.	Area alkyne CH peak for benzoic acid 1-mer (2.29 ppm)	Area alkyne CH peak for phenol 1-mer (2.27 ppm)	$\chi_{mutator}$
**1**	100	0	**0**
**2**	1577275.3	956123.6	**0.38**
**3**	4893437.4	5688666.4	**0.54**
**4**	1445689.2	4396242.6	**0.75**
**5**	0	100	**1**

Step 2: ZIP.

Scheme S7 shows the intramolecular CuAAC (ZIP) reaction of the preZIPs intermediates from the five different experiments carried out in the previous section.

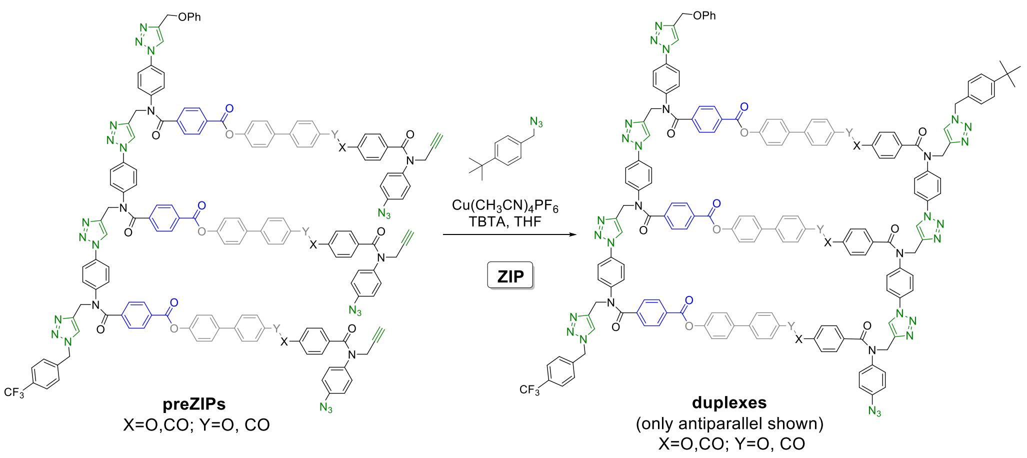

Scheme S7.

➢ **General procedure for ZIP reaction.**

A solution of 1-(azidomethyl)-4-*tert*-butylbenzene[S7] in dry and degassed THF (1 mL) was added to a solution of pre-ZIP intermediates in dry and degassed THF under N_2 atmosphere. $Cu(CH_3CN)_4PF_6$ and TBTA were added to the solution and the reaction stirred at room temperature for 2 days. After evaporation of the solvent, the crude was precipitated with Pet. Ether and centrifuged (repeated three times) in order to remove the excess of capping azide. The obtained solid was used in the next step without further purification.

➢ **Experiment 1.**

Reagents and solvent: preZIP **S9** (0.013 g, 0.005 mmol), 1-(azidomethyl)-4-*tert*-butylbenzene (0.069 g, 0.363 mmol), $Cu(CH_3CN)_4PF_6$ (0.011 g, 0.029 mmol), TBTA (0.013 g, 0.029 mmol) and THF (320 mL).

Fig. S9 shows the UPLC traces of the starting preZIP intermediate **S9** and reaction crude.

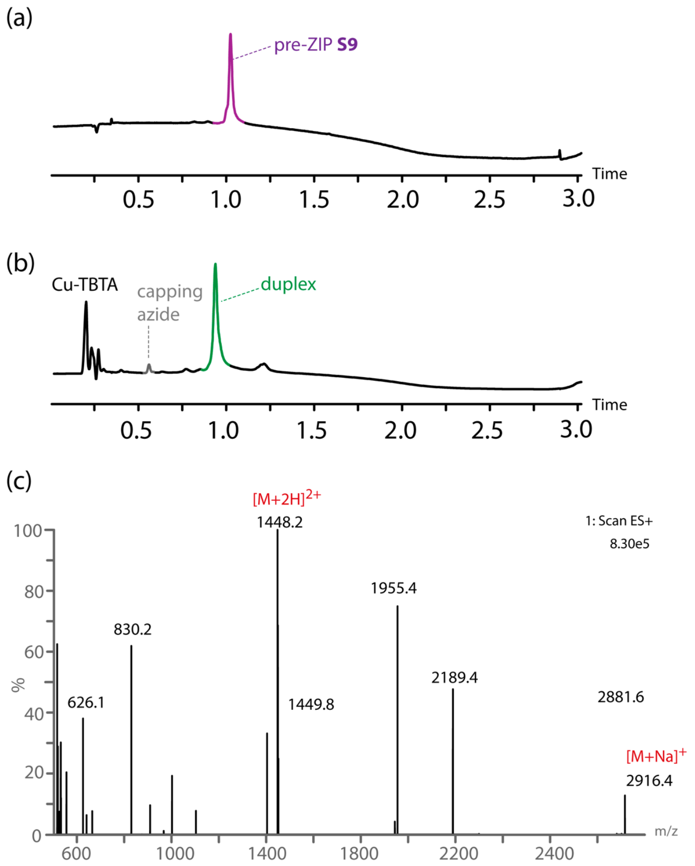

Fig S9. UPLC traces of: (a) starting preZIP **S9**. (b) Reaction crude for the ZIP step. (c) MS spectrum of duplex intermediate (MW: 2892.9). *UPLC Conditions*: C8 column at 40 ºC (254 nm) using water + 0.1% formic acid (A) and CH_3CN + 0.1% formic acid (B) with flow rate of 0.6 ml/min; Gradient of 0-2 minutes 65% -100% B + 1 minute 100% B.

➢ **Experiment 2.**

Reagents and solvent: preZIPs from the attach experiment 2 (0.006 g, 0.002 mmol), 1-(azidomethyl)-4-*tert*-butylbenzene (0.029 g, 0.155 mmol), $Cu(CH_3CN)_4PF_6$ (0.005 g, 0.012 mmol), TBTA (0.007 g, 0.012 mmol) and THF (138 mL).

Fig. S10 shows the UPLC traces of the starting preZIP intermediates and reaction crude.

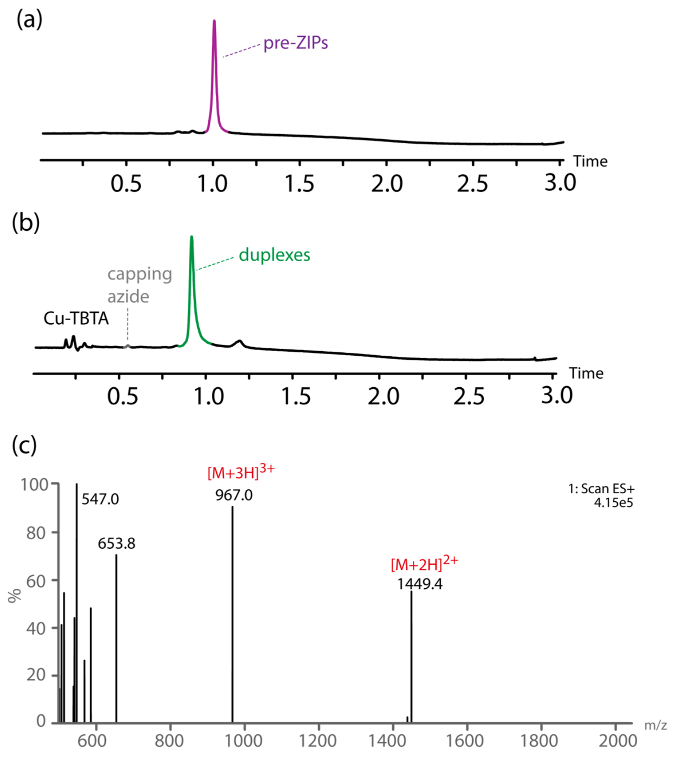

Fig S10. UPLC traces of: (a) starting preZIP intermediates. (b) Reaction crude for the ZIP step. (c) MS spectrum of duplex intermediate (MW: 2892.9). *UPLC Conditions*: C8 column at 40 ºC (254 nm) using water + 0.1% formic acid (A) and CH_3CN + 0.1% formic acid (B) with flow rate of 0.6 ml/min; Gradient of 0-2 minutes 65% -100% B + 1 minute 100% B.

➢ **Experiment 3.**

Reagents and solvent: preZIPs from the attach experiment 3 (0.009 g, 0.003 mmol), 1-(azidomethyl)-4-*tert*-butylbenzene (0.047 g, 0.250 mmol), $Cu(CH_3CN)_4PF_6$ (0.008 g, 0.020 mmol), TBTA (0.011 g, 0.020 mmol) and THF (222 mL).

Fig. S11 shows the UPLC traces of the starting preZIP intermediates and reaction crude.

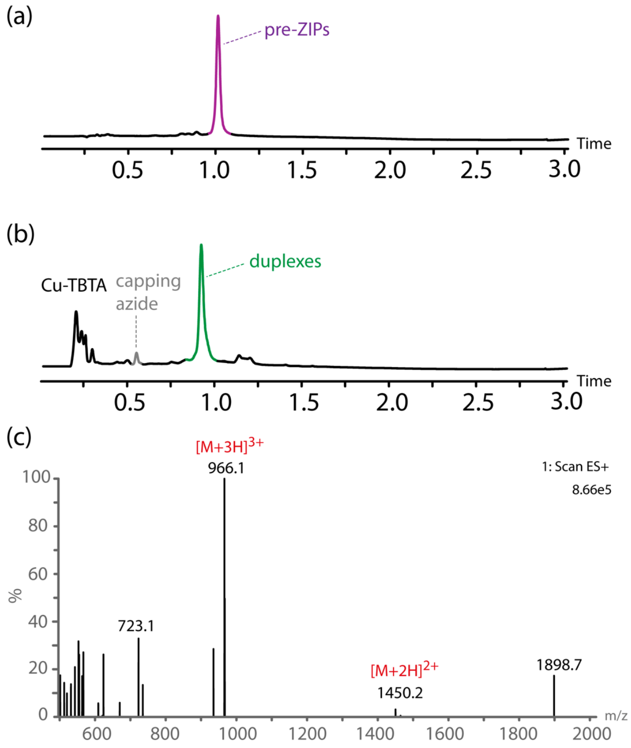

Fig S11. UPLC traces of: (a) starting preZIP intermediates. (b) Reaction crude for the ZIP step. (c) MS spectrum of duplex intermediate (MW: 2892.9). *UPLC Conditions*: C8 column at 40 °C (254 nm) using water + 0.1% formic acid (A) and CH_3CN + 0.1% formic acid (B) with flow rate of 0.6 ml/min; Gradient of 0-2 minutes 65% -100% B + 1 minute 100% B.

- **Experiment 4.**

Reagents and solvent: preZIPs from the attach experiment 4 (0.007 g, 0.003 mmol), 1-(azidomethyl)-4-*tert*-butylbenzene (0.038 g, 0.199 mmol), $Cu(CH_3CN)_4PF_6$ (0.006 g, 0.016 mmol), TBTA (0.009 g, 0.016 mmol) and THF (177 mL).

Fig. S12 shows the UPLC traces of the starting preZIP intermediates and reaction crude.

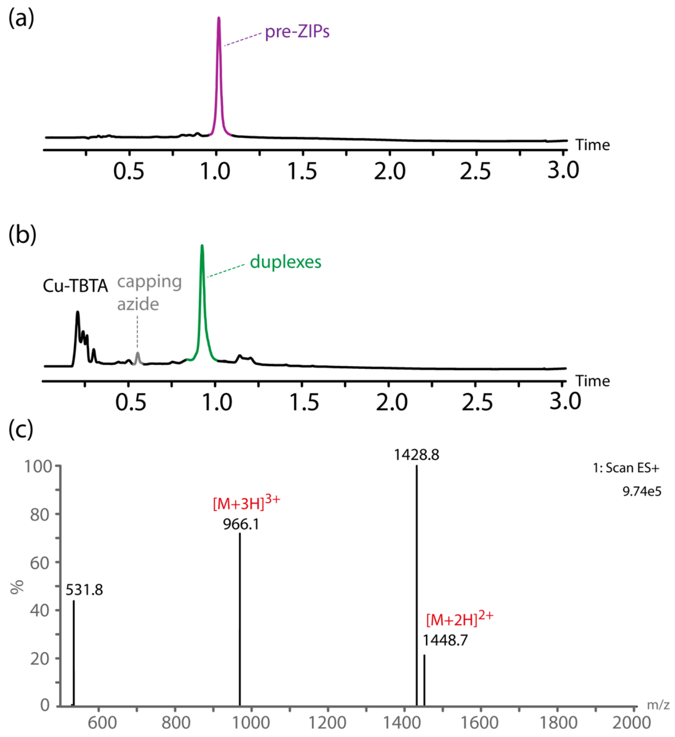

Fig S12. UPLC traces of: (a) starting preZIP intermediates. (b) Reaction crude for the ZIP step. (c) MS spectrum of duplex intermediate (MW: 2892.9). *UPLC Conditions*: C8 column at 40 ºC (254 nm) using water + 0.1% formic acid (A) and CH_3CN + 0.1% formic acid (B) with flow rate of 0.6 ml/min; Gradient of 0-2 minutes 65% -100% B + 1 minute 100% B.

- **Experiment 5.**

Reagents and solvent: preZIP **S10** (0.010 g, 0.004 mmol), 1-(azidomethyl)-4-*tert*-butylbenzene (0.052 g, 0.275 mmol), $Cu(CH_3CN)_4PF_6$ (0.008 g, 0.022 mmol), TBTA (0.012 g, 0.022 mmol) and THF (246 mL).

Fig. S13 shows the UPLC traces of the starting preZIP intermediate **S10** and reaction crude.

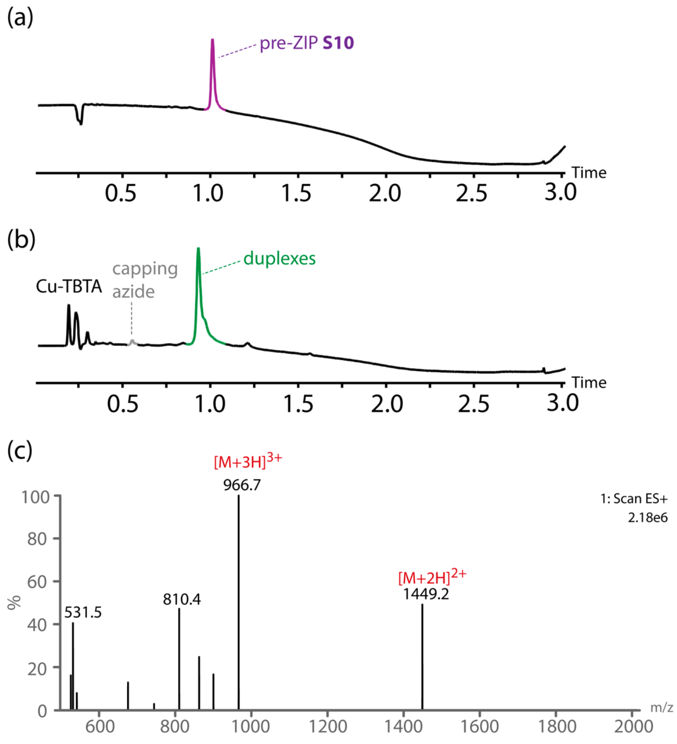

Fig S13. UPLC traces of: (a) starting preZIP **S10**. (b) Reaction crude for the ZIP step. (c) MS spectrum of duplex intermediate (MW: 2892.9). *UPLC Conditions*: C8 column at 40 ºC (254 nm) using water + 0.1% formic acid (A) and CH_3CN + 0.1% formic acid (B) with flow rate of 0.6 ml/min; Gradient of 0-2 minutes 65% -100% B + 1 minute 100% B.

Step 3: Capping.

Scheme S8 shows the CuAAC capping with phenyl propargyl ether of the duplexes from the five different experiments carried out in the previous section.

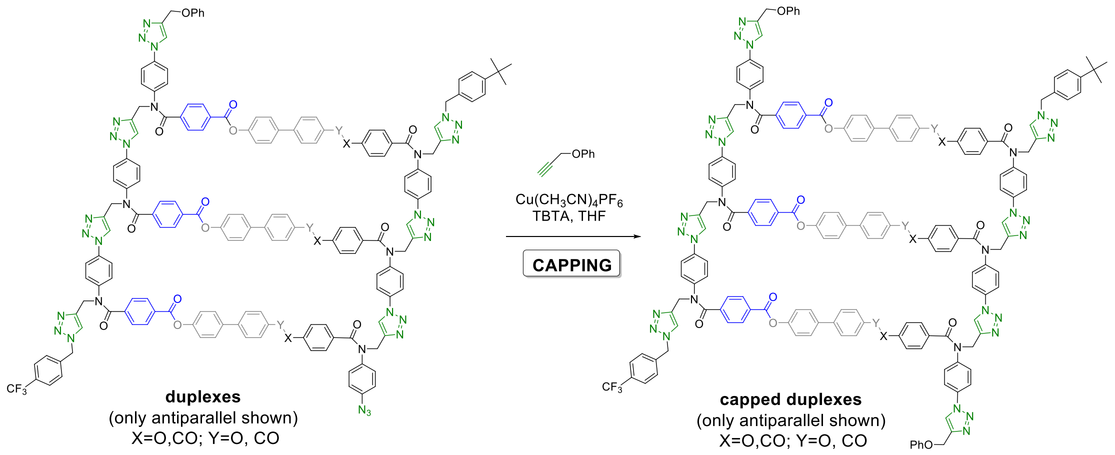

Scheme S8.

➢ **General procedure for capping reaction.**

Phenyl propargyl ether was added to a solution of the corresponding duplex mixtures in dry and degassed THF. $Cu(CH_3CN)_4PF_6$ and TBTA were added to the previous solution and the reaction stirred overnight at room temperature. After evaporation of the solvent, the crude was precipitated with Pet. Ether and centrifuged (repeated three times) in order to remove the excess of capping alkyne. The obtained solid was used in the next step without further purification.

Experiment 1.

Reagents and solvent: duplex from ZIP experiment 1 (0.005 mmol), phenyl propargyl ether (0.060 mL, 0.484 mmol), $Cu(CH_3CN)_4PF_6$ (0.002 g, 0.005 mmol), TBTA (0.003 g, 0.005 mmol) and THF (2 mL).

Fig. S14 shows the UPLC traces of the crude reaction mixtures for the ZIP and capping steps.

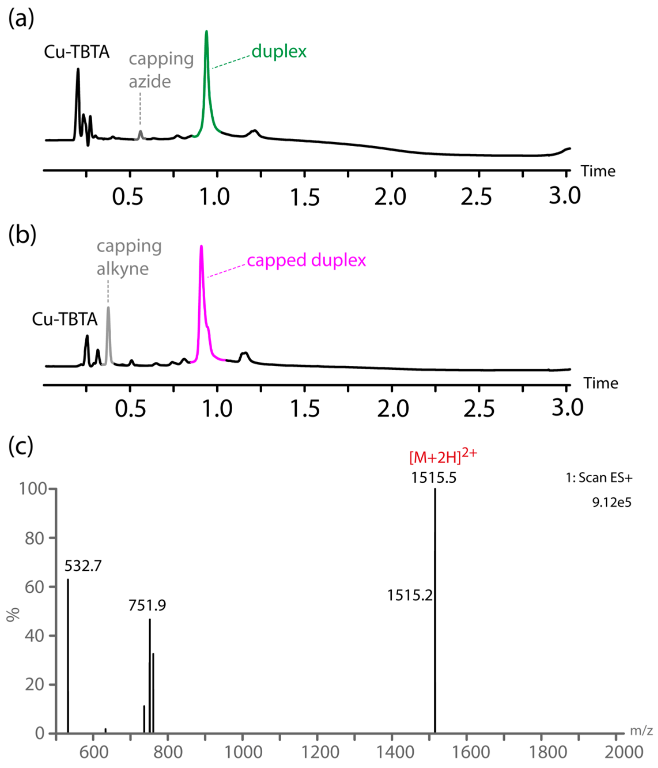

Fig S14. UPLC traces of: (a) Reaction crude for the ZIP step from experiment 1. (b) Reaction crude for the capping step. (c) MS spectrum of capped duplex intermediate (MW: 3024.9). *UPLC Conditions*: C8 column at 40 °C (254 nm) using water + 0.1% formic acid (A) and CH_3CN + 0.1% formic acid (B) with flow rate of 0.6 ml/min; Gradient of 0-2 minutes 65% -100% B + 1 minute 100% B.

Experiment 2.

Reagents and solvent: duplex from ZIP experiment 2 (0.002 mmol), phenyl propargyl ether (0.027 mL, 0.207 mmol), $Cu(CH_3CN)_4PF_6$ (0.001 g, 0.003 mmol), TBTA (0.001 g, 0.003 mmol) and THF (3 mL).

Fig. S15 shows the UPLC traces of the crude reaction mixtures for the ZIP and capping steps.

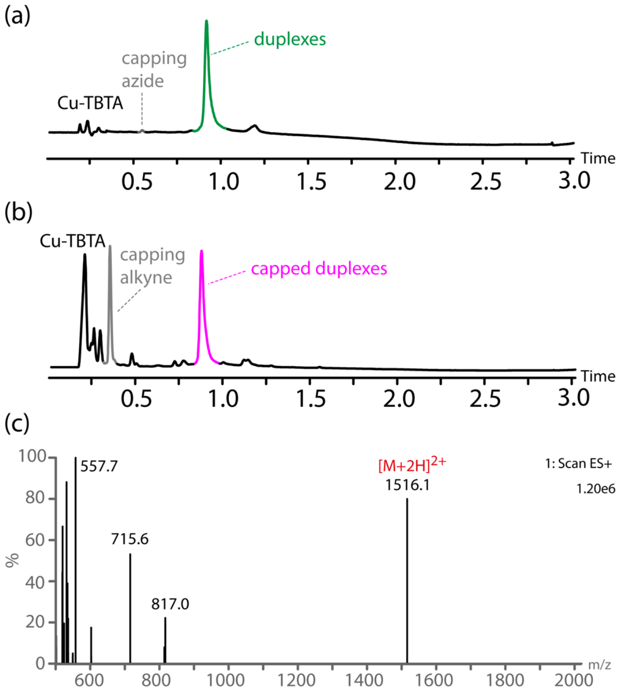

Fig S15. UPLC traces of: (a) Reaction crude for the ZIP step from experiment 2. (b) Reaction crude for the capping step. (c) MS spectrum of capped duplex intermediate (MW: 3024.9). *UPLC Conditions*: C8 column at 40 °C (254 nm) using water + 0.1% formic acid (A) and CH_3CN + 0.1% formic acid (B) with flow rate of 0.6 ml/min; Gradient of 0-2 minutes 65% -100% B + 1 minute 100% B.

Experiment 3.

Reagents and solvent: duplex from ZIP experiment 3 (0.003 mmol), phenyl propargyl ether (0.042 mL, 0.330 mmol), $Cu(CH_3CN)_4PF_6$ (0.001 g, 0.003 mmol), TBTA (0.001 g, 0.003 mmol) and THF (5 mL).

Fig. S16 shows the UPLC traces of the crude reaction mixtures for the ZIP and capping steps.

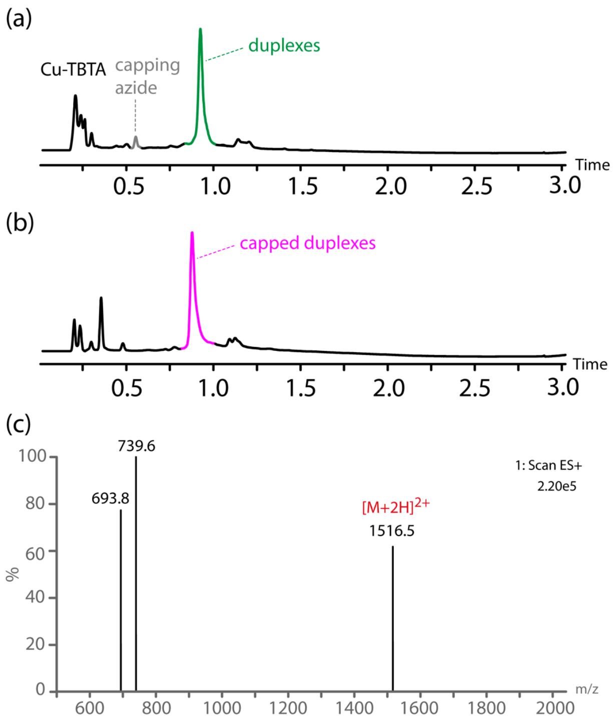

Fig S16. UPLC traces of: (a) Reaction crude for the ZIP step from experiment 3. (b) Reaction crude for the capping step. (c) MS spectrum of capped duplex intermediate (MW: 3024.9). *UPLC Conditions*: C8 column at 40 °C (254 nm) using water + 0.1% formic acid (A) and CH_3CN + 0.1% formic acid (B) with flow rate of 0.6 ml/min; Gradient of 0-2 minutes 65% -100% B + 1 minute 100% B.

Experiment 4.

Reagents and solvent: duplex from ZIP experiment 4 (0.003 mmol), phenyl propargyl ether (0.034 mL, 0.266 mmol), $Cu(CH_3CN)_4PF_6$ (0.001 g, 0.003 mmol), TBTA (0.001 g, 0.003 mmol) and THF (3 mL).

Fig. S17 shows the UPLC traces of the crude reaction mixtures for the ZIP and capping steps.

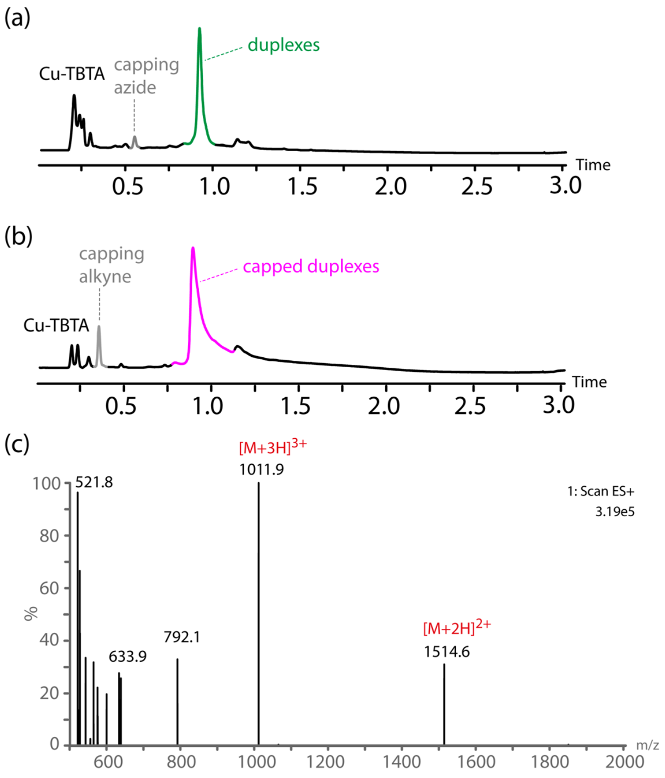

Fig S17. UPLC traces of: (a) Reaction crude for the ZIP step from experiment 4. (b) Reaction crude for the capping step. (c) MS spectrum of capped duplex intermediate (MW: 3024.9). *UPLC Conditions*: C8 column at 40 °C (254 nm) using water + 0.1% formic acid (A) and CH_3CN + 0.1% formic acid (B) with flow rate of 0.6 ml/min; Gradient of 0-2 minutes 65% -100% B + 1 minute 100% B.

Experiment 5.

Reagents and solvent: duplex from ZIP experiment 5 (0.004 mmol), phenyl propargyl ether (0.047 mL, 0.370 mmol), $Cu(CH_3CN)_4PF_6$ (0.001 g, 0.004 mmol), TBTA (0.002 g, 0.004 mmol) and THF (5 mL).

Fig. S18 shows the UPLC traces of the crude reaction mixtures for the ZIP and capping steps.

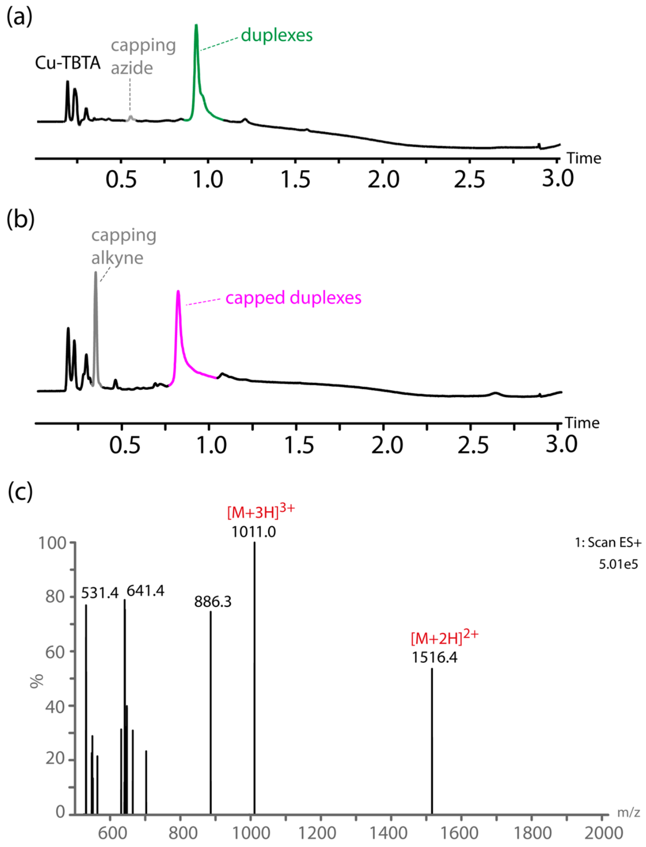

Fig S18. UPLC traces of: (a) Reaction crude for the ZIP step from experiment 5. (b) Reaction crude for the capping step. (c) MS spectrum of capped duplex intermediate (MW: 3024.9). *UPLC Conditions*: C8 column at 40 °C (254 nm) using water + 0.1% formic acid (A) and CH_3CN + 0.1% formic acid (B) with flow rate of 0.6 ml/min; Gradient of 0-2 minutes 65% -100% B + 1 minute 100% B.

Step 4: Hydrolysis.

Scheme S9 shows the cleavage step of the capped duplexes from the five different experiments carried out in the previous section. Basic hydrolysis of the ester bonds regenerates the template **1** along with products **8**-**15**, whose proportion is related to the mutation rate, and the traceless biphenyl linker.

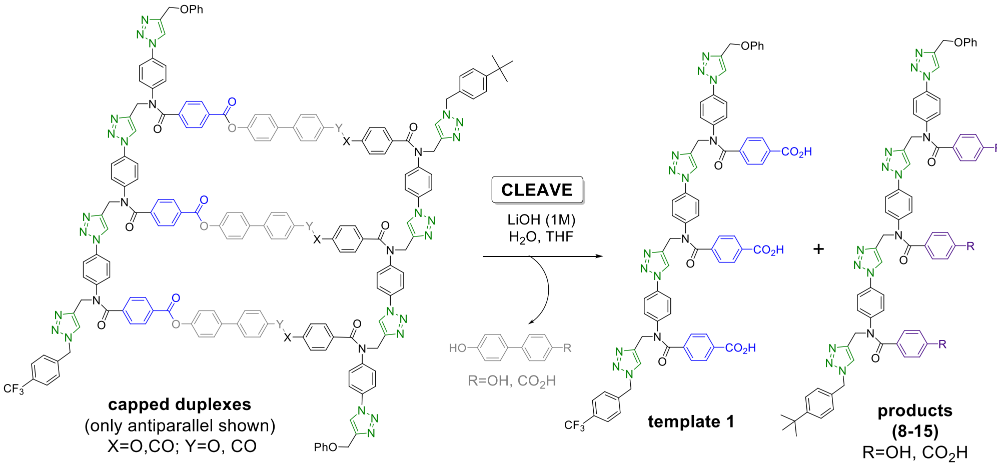

Scheme S9.

➢ **General procedure for cleavage.**

The crude reaction mixture from previous step was dissolved in THF:H_2O 3:1 and 1 M LiOH soln. was added. The solution was left to react overnight at 5 °C. Then, the crude was diluted with H_2O and acidified with 0.1 M HCl soln. to pH 2-3. The aqueous phase was extracted with EtOAc (3x) and the combined organic phase was washed with 0.01M EDTA soln. (2x), H_2O (1x), brine (1x), dried over $MgSO_4$ and evaporated to dryness.

➢ **Experiment 1.**

Reagents and solvent: capped duplex from capping experiment 1 (0.005 mmol), 1 M LiOH soln. (0.20 mL, 0.20 mmol), THF:H_2O 3:1 (4 mL). After work-up, the obtained residue was purified by flash column chromatography on silica gel using a gradient from 0% to 20% of MeOH (containing 0.01% of aq. HCl) in CH_2Cl_2.

Fig. S19 shows the UPLC traces of the reaction crude and after purification along with the MS spectra of the recovered template (**1**) and templated product AAA (**8**).

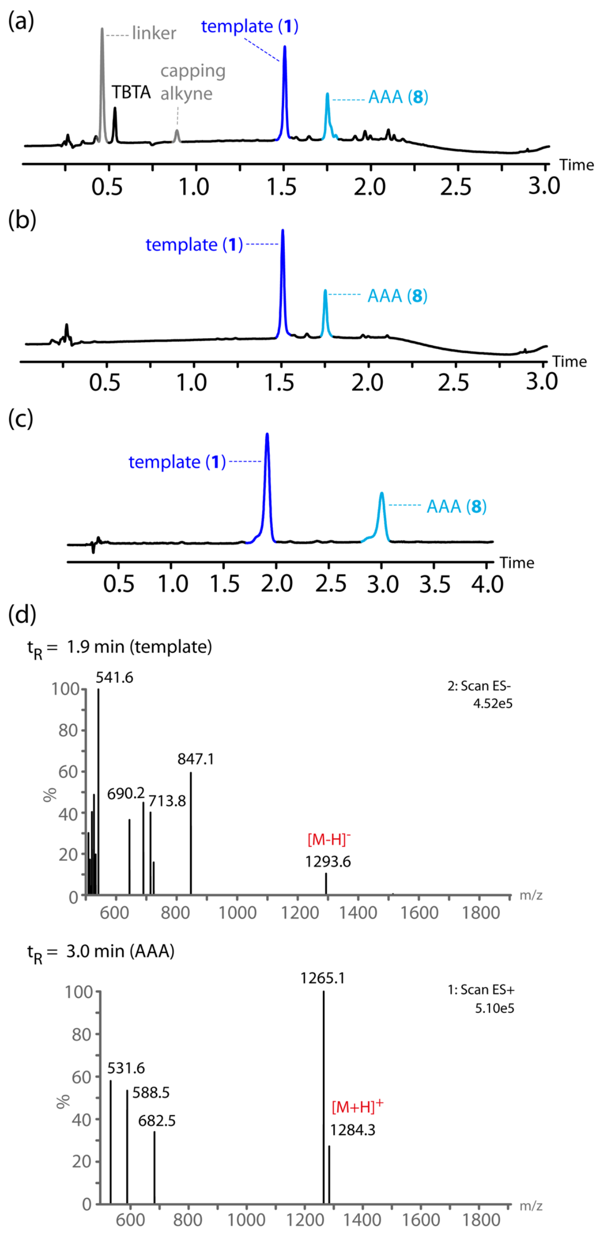

Fig S19. UPLC traces of: (a) Reaction crude for the cleavage step for experiment 1. (b) After chromatography purification. (c) After chromatography purification using a different UPLC gradient (see details below). (d) MS spectra of the recovered template **1** (t_R = 1.9 min in the chromatogram shown in (c); MW = 1294.2) and the templated product AAA **8** (t_R = 3.0 min in the chromatogram shown in (c); MW = 1282.4). *UPLC Conditions*: C18 column at 40 °C (254 nm) using water + 0.1% formic acid (A) and CH_3CN + 0.1% formic acid (B) with flow rate of 0.6 ml/min; Gradient of 0-2 minutes 35% -65% B + 1 minute 100% B for (a) and (b). Gradient of 0-4 minutes from 45% to 50% B + 1 minute 100% B for (c).

- **Experiment 2.**

Reagents and solvent: capped duplex from capping experiment 2 (0.002 mmol), 1 M LiOH soln. (0.10 mL, 0.10 mmol), THF:H_2O 3:1 (2 mL).

Fig. S20 shows the UPLC traces of the reaction crude and the MS spectra of the recovered template (**1**) and templated products **8**-**15**.

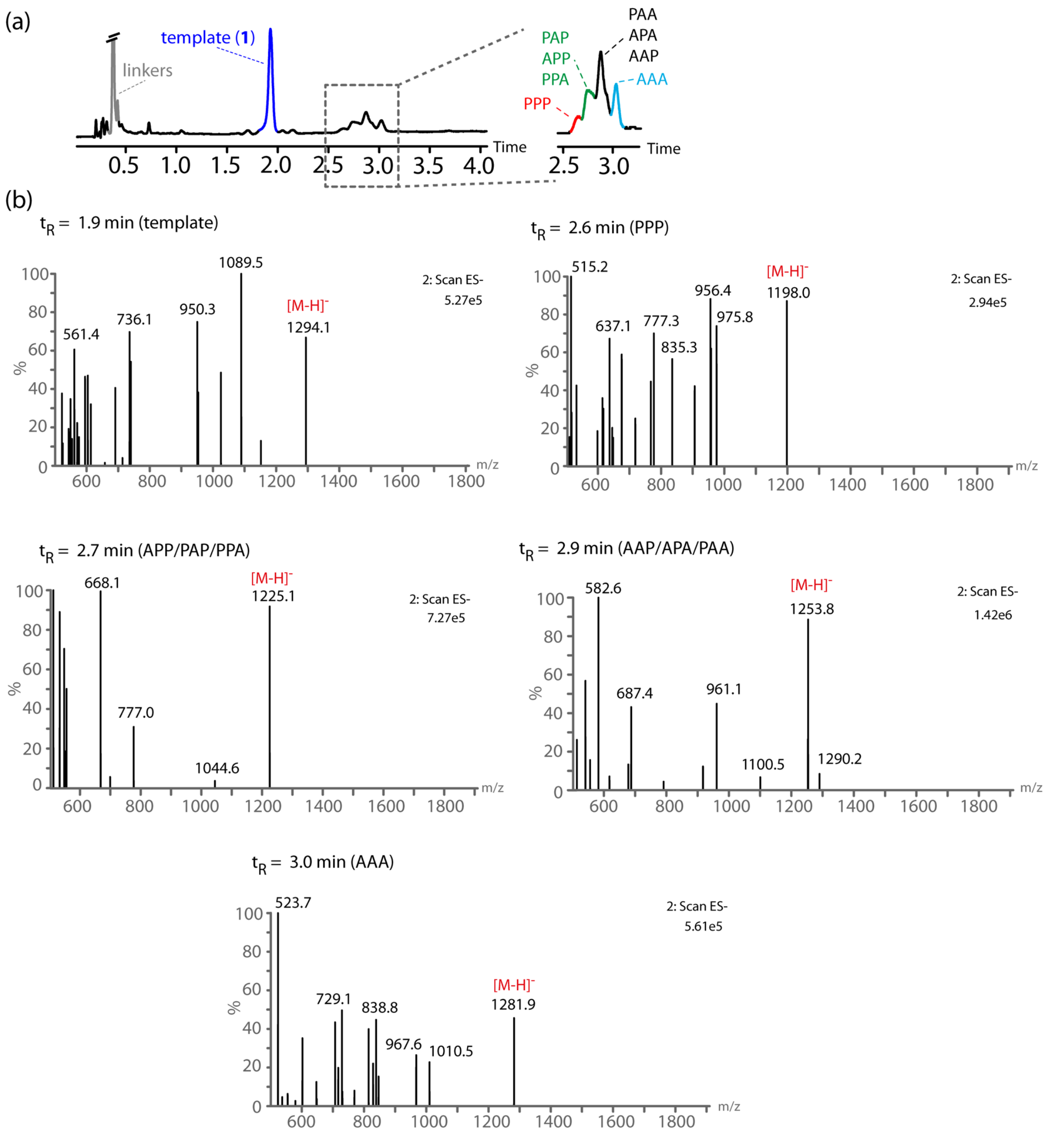

Fig S20. UPLC traces of: (a) Reaction crude for the cleavage step for experiment 2. Amplification of the region corresponding to the templated products is shown on the right. (b) MS spectra of the recovered template **1** (t_R = 1.9 min; MW = 1294.2) and the templated products PPP **15** (t_R = 2.6 min; MW = 1198.3), APP/PAP/PPA **12**-**14** (t_R = 2.7 min; MW = 1226.3), AAP/APA/PAA **9**-**11** (t_R = 2.9 min; MW = 1254.3) and AAA **8** (t_R = 3.0 min; MW = 1282.4). *UPLC Conditions*: C18 column at 40 °C (254 nm) using water + 0.1% formic acid (A) and CH_3CN + 0.1% formic acid (B) with flow rate of 0.6 ml/min; Gradient of 0-4 minutes from 45% to 50% B + 1 minute 100% B.

- **Experiment 3.**

Reagents and solvent: capped duplex from capping experiment 3 (0.003 mmol), 1 M LiOH soln. (0.10 mL, 0.10 mmol), THF:H_2O 3:1 (2 mL).

Fig. S21 shows the UPLC traces of the reaction crude and the MS spectra of the recovered template (**1**) and templated products **8**-**15**.

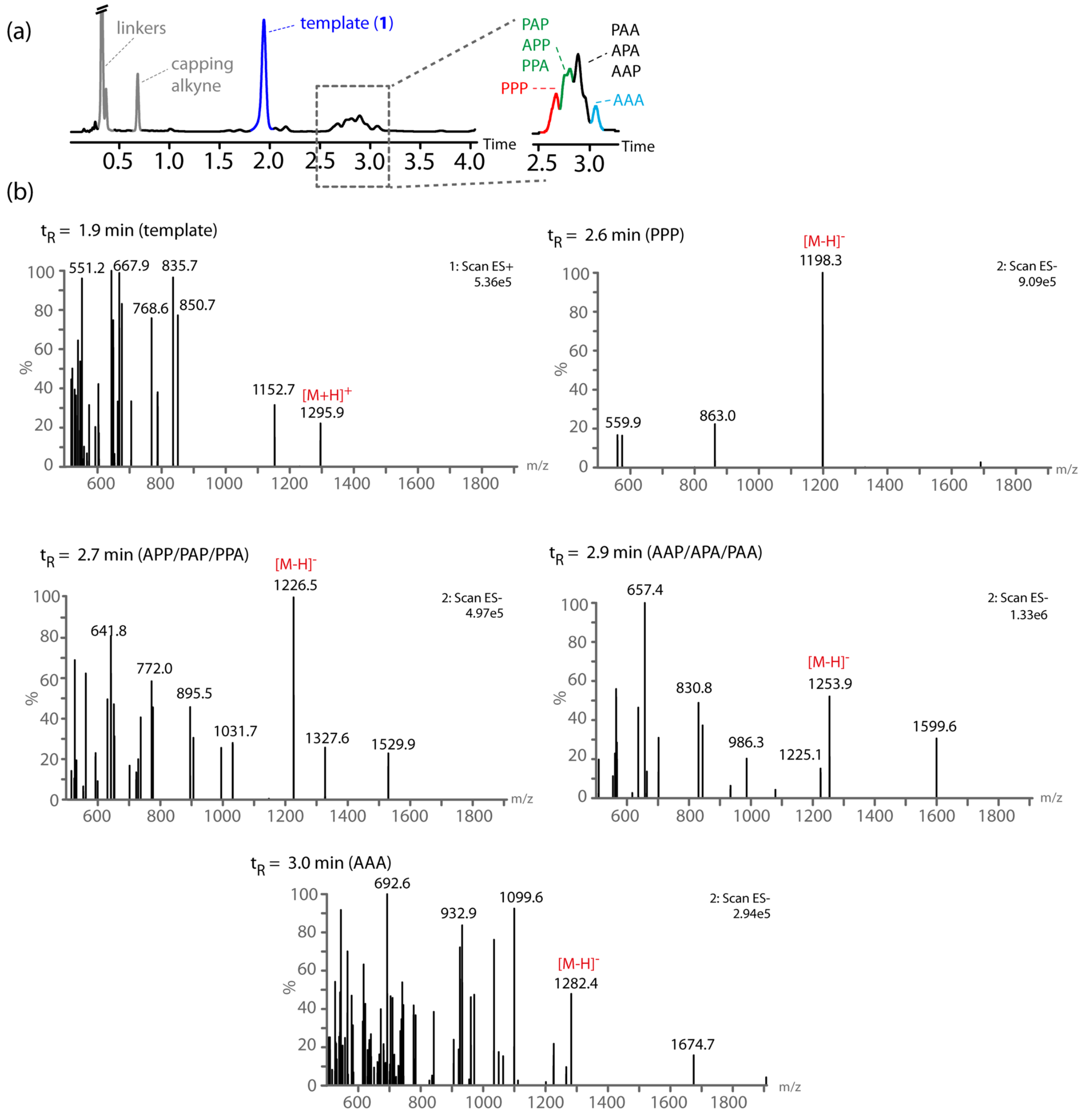

Fig S21. UPLC traces of: (a) Reaction crude for the cleavage step for experiment 3. Amplification of the region corresponding to the templated products is shown on the right. (b) MS spectra of the recovered template **1** (t_R = 1.9 min; MW = 1294.2) and the templated products PPP **15** (t_R = 2.6 min; MW = 1198.3), APP/PAP/PPA **12**-**14** (t_R = 2.7 min; MW = 1226.3), AAP/APA/PAA **9**-**11** (t_R = 2.9 min; MW = 1254.3) and AAA **8** (t_R = 3.0 min; MW = 1282.4). *UPLC Conditions*: C18 column at 40 °C (254 nm) using water + 0.1% formic acid (A) and CH_3CN + 0.1% formic acid (B) with flow rate of 0.6 ml/min; Gradient of 0-4 minutes from 45% to 50% B + 1 minute 100% B.

➢ **Experiment 4.**

Reagents and solvent: capped duplex from capping experiment 3 (0.003 mmol), 1M LiOH soln. (0.10 mL, 0.10 mmol), THF:H_2O 3:1 (2 mL).

Fig. S22 shows the UPLC traces of the reaction crude and the MS spectra of the recovered template (**1**) and templated products **8**-**15**.

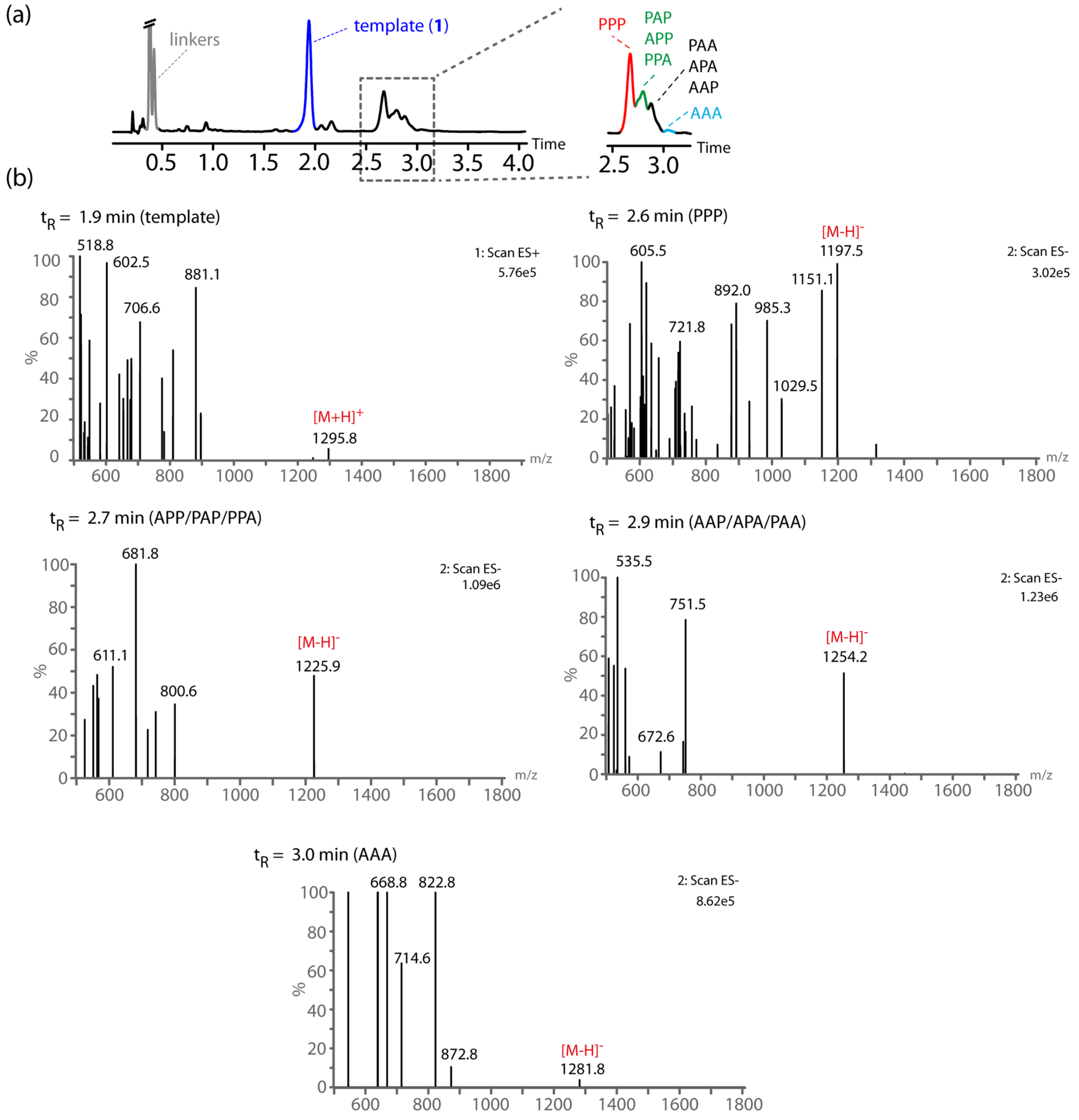

Fig S22. UPLC traces of: (a) Reaction crude for the cleavage step for experiment 4. Amplification of the region corresponding to the templated products is shown on the right. (b) MS spectra of the recovered template **1** (t_R = 1.9 min; MW = 1294.2) and the templated products PPP **15** (t_R = 2.6 min; MW = 1198.3), APP/PAP/PPA **12**-**14** (t_R = 2.7 min; MW = 1226.3), AAP/APA/PAA **9**-**11** (t_R = 2.9 min; MW = 1254.3) and AAA **8** (t_R = 3.0 min; MW = 1282.4). *UPLC Conditions*: C18 column at 40 °C (254 nm) using water + 0.1% formic acid (A) and CH_3CN + 0.1% formic acid (B) with flow rate of 0.6 ml/min; Gradient of 0-4 minutes from 45% to 50% B + 1 minute 100% B.

- **Experiment 5.**

Reagents and solvent: capped duplex from capping experiment 5 (0.004 mmol), 1M LiOH soln. (0.20 mL, 0.20 mmol), THF:H_2O 3:1 (4 mL). After work-up, the obtained residue was purified by flash column chromatography on silica gel using a gradient from 0% to 20% of MeOH (containing 0.01% of aq. HCl) in CH_2Cl_2.

Fig. S23 shows the UPLC traces of the reaction crude and after purification along with the MS spectra of the recovered template (**1**) and templated product PPP (**15**).

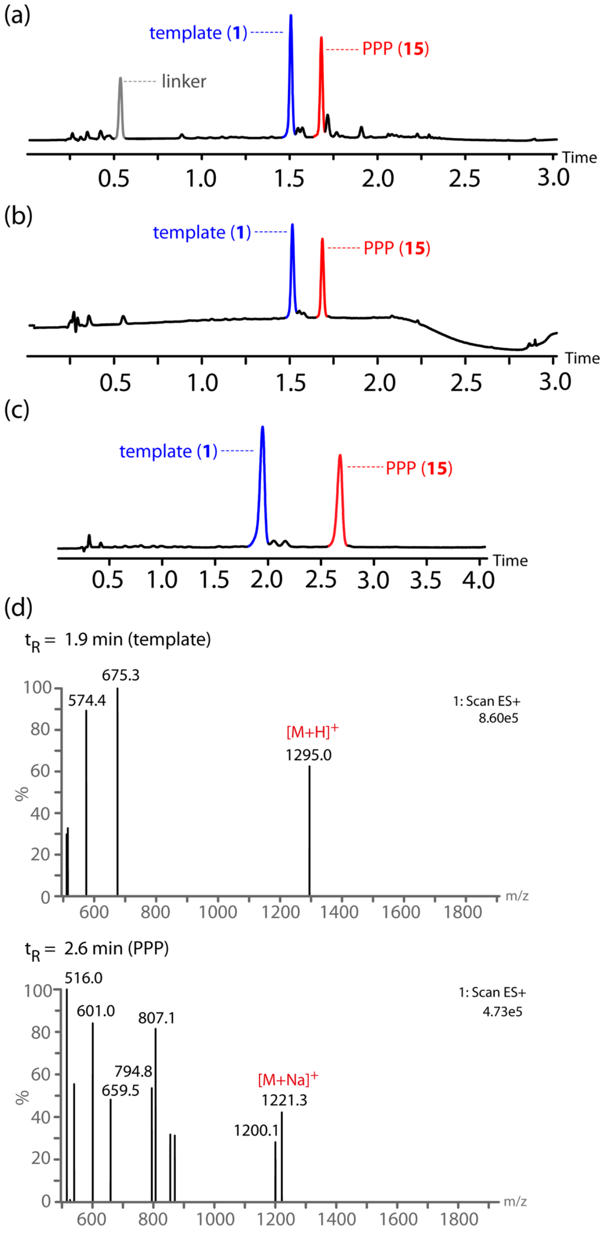

Fig S23. UPLC traces of: (a) Reaction crude for the cleavage step for experiment 5. (b) After chromatography purification. (c) After chromatography purification using a different UPLC gradient (see details below). (d) MS spectra of the recovered template **1** (t_R = 1.9 min in the chromatogram shown in (c); MW = 1294.2) and the templated product PPP **15** (t_R = 2.6 min in the chromatogram shown in (c); MW = 1198.3). *UPLC Conditions*: C18 column at 40 °C (254 nm) using water + 0.1% formic acid (A) and CH_3CN + 0.1% formic acid (B) with flow rate of 0.6 ml/min; Gradient of 0-2 minutes 35% -65% B + 1 minute 100% B for (a) and (b). Gradient of 0-4 minutes from 45% to 50% B + 1 minute 100% B for (c).

Determination of sequence population after covalent template-directed mutation

The populations of the obtained products **8**-**15** were calculated by deconvoluting the UPLC peaks using the open-source curve-fitting software Fityk (version 1.3.1).[S8] The UPLC traces were exported in CSV format from Waters MassLynx™ software (version 4.2) using Microsoft® Excel®. The peaks corresponding to the templated products **8**-**15** were fitted to a Gaussian function using the PRAXIS fitting method provided in Fityk. PAP-APP and PAA-APA pairs were fitted to single Gaussian functions as their peaks are not resolved enough. Fig. S24 shows the fitting model, individual functions and residuals obtained in experiments 2-4.

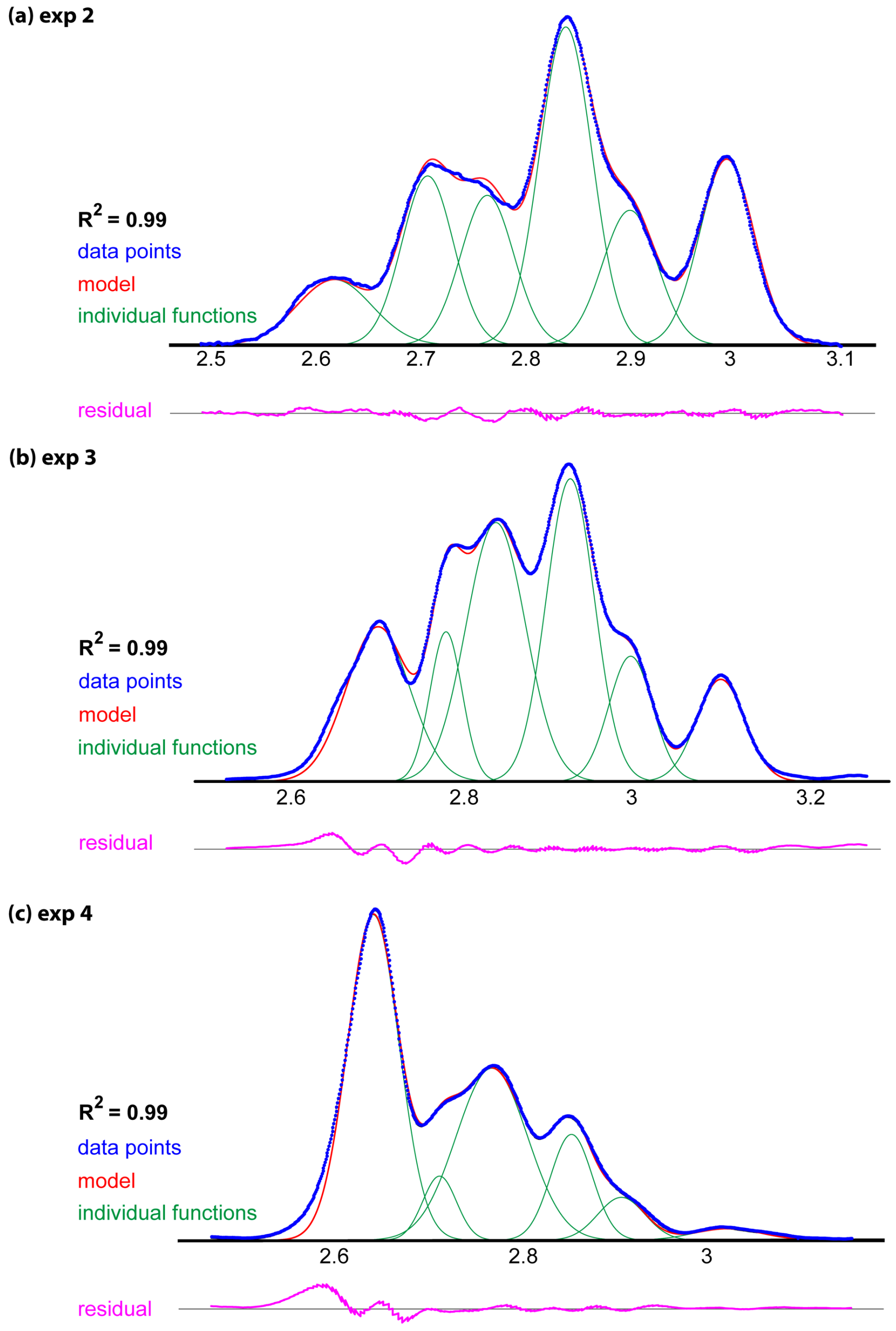

Fig. S24. Deconvolution of the UPLC peaks for the templated products from experiments 2-4 using Fityk 1.3.1. PAP-APP and PAA-APA pairs were fitted to single gaussian functions. Experimental signal is shown in blue, fitting model in red, individual functions in green and residuals in pink.

Tables S2-4 show the areas of the individual curves and the population of each sequence extracted from the fitted UPLC curves for experiments 2-4, which are the values used in the *y* axis in Fig. 8 in the text (for experiments 1 and 5, the single templated product obtained is considered as 100%).

Table S2. Areas of the fitted curves shown in green in Fig. S24 for experiment 2.

	PPP	PPA	PAP/APP	PAA/APA	AAP	AAA
**t_R**	2.6257	2.71461	2.77145	2.84628	2.90734	2.99975
**absolute area**	156.531	278.822	253.786	532.01	235.688	322.738
**population (%)**	8.8	15.7	14.3	29.9	13.2	18.1
**population per sequence family (%)**	**8.8**	**29.9**		**43.1**		**18.1**

Table S3. Areas of the fitted curves shown in green in Fig. S24 for experiment 3.

	PPP	PPA	PAP/APP	PAA/APA	AAP	AAA
**t_R**	2.70492	2.78346	2.841	2.92695	2.99661	3.10064
**absolute area**	2283.63	1115.44	3578.06	3386.26	1272.94	1145.19
**population (%)**	17.9	8.7	28.0	26.5	10.0	9.0
**population per sequence family (%)**	**17.9**	**36.7**		**36.5**		**9.0**

Table S4. Areas of the fitted curves shown in green in Fig. S24 for experiment 4.

	PPP	PPA	PAP/APP	PAA/APA	AAP	AAA
**t_R**	2.65151	2.7216	2.77688	2.86114	2.91394	3.02657
**absolute area**	4445.78	575.158	3253.2	1173.85	537.166	210.923
**population (%)**	43.6	5.6	31.9	11.5	5.3	2.1
**population per sequence family (%)**	**43.6**	**37.5**		**16.8**		**2.1**

References

[S1] D. Núñez-Villanueva, M. Ciaccia, G. Iadevaia, E. Sanna, C. A. Hunter. *Chem. Sci.* **2019**, *10*, 5258.

[S2] E. R. Zubarev, M. U. Pralle, E. D. Sone, S. I. Stupp. *J. Am. Chem. Soc.* **2001**, *123*, 4105.

[S3] P. Gopalan, X. Li, M. Li, C. K. Ober, C. P. Gonzales, C. J. Hawker. *J. Polym. Sci. Part A: Polym. Chem.* **2003**, *41*, 3640.

[S4] D. Núñez-Villanueva, C. A. Hunter. *Org. Biomol. Chem.* **2019**, *17*, 9660.

[S5] M. Ciaccia, D. Núñez-Villanueva, C. A. Hunter. *J. Am. Chem. Soc.* **2019**, *141*, 10862.

[S6] D. Núñez-Villanueva, M. Ciaccia, C. A. Hunter. *RSC Adv.* **2019**, *9*, 29566.

[S7] Y.-S. Lee, S. M. Park, H. M. Kim, S.-K. Park, K. Lee, C. W. Lee, B. H. Kim. *Bioorg. Med. Chem. Lett.* **2009**, *19*, 4688.

[S8] https://fityk.nieto.pl/.
